# Heteroaryl Bishydrazono Nitroimidazoles: A Unique Structural Skeleton with Potent Multitargeting Antibacterial Activity

**DOI:** 10.3390/ijms262411836

**Published:** 2025-12-08

**Authors:** Zhen-Zhen Li, Cheng-He Zhou, Yi-Jin Liu

**Affiliations:** 1 State Key Laboratory of Medicinal Chemical Biology, Frontiers Science Center for New Organic Matter, Frontiers Science Center for Cell Responses, College of Pharmacy, Nankai University, Tianjin 300071, China; 2 Key Laboratory of Applied Chemistry of Chongqing Municipality, Institute of Bioorganic & Medicinal Chemistry, School of Chemistry and Chemical Engineering, Southwest University, Chongqing 400715, China

**Keywords:** imidazole, nitrofurantoin, hydrazone, antibacterial, drug resistance, membrane

## Abstract

The emergence of bacterial infections as a critical public health challenge underscores the urgent need for innovative therapeutic strategies. We designed and synthesized a series of novel heteroaryl bishydrozono nitroimidazoles and their analogs by strategically hybridizing multiple molecular components through diverse linkers. Among the newly synthesized compounds, compound **4** displayed a broad-spectrum antibacterial profile with low cytotoxicity and hemolysis. It could inhibit the proliferation of methicillin-resistant *Staphylococcus aureus* and reduce its metabolic activity with low bacterial resistance. Further investigations revealed that the highly active compound **4** could not only disrupt cell membrane integrity and induce excessive reactive oxygen species within bacterial membranes, but also intercalate into DNA to form a supramolecular **4**-DNA gyrase complex and cause cell death. These results demonstrated that compound **4** should have large potential as a promising candidate in the ongoing battle against resistant bacterial infections.

## 1. Introduction

Bacterial infections caused by opportunistic and pathogenic microorganisms have emerged as a critical public health challenge. The overconsumption and misuse of antibiotics have unfortunately accelerated the proliferation of multi-drug-resistant microbes, exacerbating this global health concern [1,2,3]. Methicillin-resistant *Staphylococcus aureus* (MRSA) represents a quintessential example of bacterial adaptation and antibiotic resistance [4,5]. Since its first identification in the early 1960s, this bacterium has developed resistance to multiple antibiotics, presenting a complex medical challenge. MRSA infections range from mild skin conditions to more severe and potentially life-threatening systemic infections [6,7]. The increasing prevalence and adaptability of this pathogen underscore the urgent need for innovative therapeutic strategies capable of effectively preventing and treating bacterial infections while mitigating the risk of further drug resistance [8,9,10].

**Figure 1:**
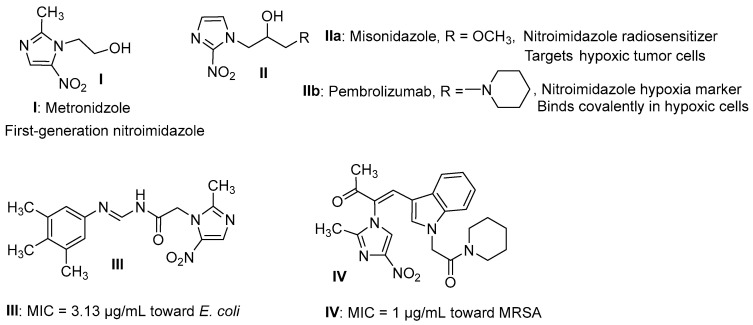
Selected examples of bioactive nitroimidazole derivatives reported in the literature [21,23,27].

**Figure 2:**
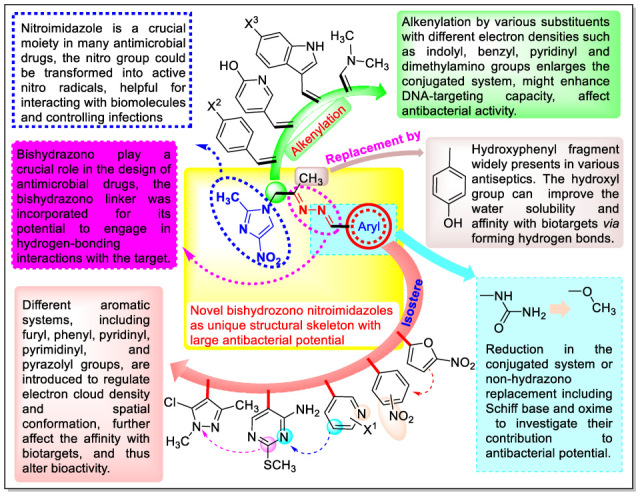
Design of novel heteroaryl bishydrozono nitroimidazoles as unique structural skeletons with large antibacterial potential.

The imidazole ring exists in many biomolecules such as histamine and histidine, as well as various clinical drugs, which have received much attention, especially for nitroimidazole [11,12]. The combination of imidazole and the nitro group could not only slightly expand the conjugation to change the charge distribution, but also enhance the occurrence of various noncovalent interactions, like hydrogen bonds, π-π stacking, and hydrophobic interactions [13,14]. The antimicrobial activity of nitroimidazole stems from its enzymatic reduction in low-redox microbial cells, which generates cytotoxic nitro radicals that damage bacterial DNA and other biomolecules [15,16,17]. Concurrently, nitroimidazole reduction can also deplete NADPH, leading to the accumulation of reactive oxygen species (ROS) and resulting in oxidative damage [18,19,20]. Clinically available nitroimidazole-based drugs like metronidazole and tinidazole are vital in anti-infective therapy [21]; however, their narrow spectrum and emerging resistance limit their utility: drugs like metronidazole have a short plasma half-life (~6–8 h), require multiple daily doses, and demonstrate poor penetration into hypoxic sites like abscesses and biofilms. Further drawbacks encompass toxicity risks—such as peripheral neuropathy and a disulfiram-like reaction with alcohol—as well as increasing resistance caused by reduced nitroreductase activation and enhanced microbial DNA repair. These limitations necessitate the development of new, efficient, broad-spectrum nitroimidazole-based agents to address serious bacterial infections [22,23,24]. Numerous studies have demonstrated that incorporating bulky structures, such as nitrofuran [25,26], indole [27], coumarin [28,29], quinolone [30], carbazole [31], and naphthalimide [32], can significantly enhance antibacterial activity while simultaneously broadening the antibacterial spectrum. Therefore, we have overwhelming interest in coupling the bulky structures with the unique nitroimidazole. Figure 1 presents a selection of pioneering nitroimidazole derivatives that have shaped the field [21,23,27]. The prototype metronidazole [**I**] established the class’s activity against anaerobic pathogens. Subsequent efforts to expand therapeutic utility yielded misonidazole [**IIa**], which exploits nitroimidazole reduction for radiosensitization in tumors, and pimonidazole [**IIb**], widely used for detecting hypoxic tissues. Building upon this legacy of functional diversity and seeking to overcome inherent limitations, we report herein the rational design of a new class of nitroimidazole conjugates. The target new nitroimidazole conjugates and their analogs were designed (Figure 2) on the basis of the following considerations:

(1) Hydrazone is of great significance in the pharmaceutical field [33]. A functional bishydrazono group is formed by the connection of two hydrazone groups (C=N–N=C) through a conjugated system. These structures typically feature π-π conjugation, which significantly influences electron delocalization and molecular stability, and incorporating hydrogen-bonding groups represents a potential strategy for improving aqueous solubility, as it can increase overall polarity and hydration capacity. Due to their distinctive properties, redox activity, and transmembrane permeability, bishydrazone derivatives have demonstrated remarkable potential in the development of novel antibacterial agents [34,35,36].

(2) The hydrazone fragment is strategically inserted as a linker to couple nitroimidazole with different aromatic rings. This strategy is beneficial in enhancing the lipophilicity or aqueous solubility of compounds, and the hybridization of nitroimidazole with diverse substituted aromatic fragments such as nitrofuran [37], aminopyrimidine [38,39], and pyridine [40,41] enlarges the conjugated system, which facilitates the interaction with target biological molecules through π-π stacking, hydrophobic interaction, van der Waals force, or hydrogen bonding [42,43].

(3) α,β-Unsaturated hydrazones (C=C–C=N) are a class of unsaturated linkers that possess both conjugated alkenes and hydrazone-based bifunctional structures. These hybrid structures helpfully enlarge the antibacterial activity of nitroimidazole through multiple synergistic mechanisms such as covalent inhibition and membrane disruption [44,45]. The methylene group at the alkenyl terminus serves as a critical pharmacophore modification site, where structural variations profoundly influence antimicrobial efficacy, selectivity, and pharmacokinetic properties. Herein, we performed systematic aromatic functionalization in the methylene group [46,47].

(4) Oximes (C=N-OH) feature both imine (C=N) and hydroxyl (-OH) groups, enabling π-conjugation, nucleophilic addition, and hydrogen bonding, which modulate polarity to regulate the lipid–water partition and improve pharmacokinetics and biocompatibility [48,49]. Oximes are a beneficial category of antibiotics, including β-lactam antibiotics [50]. Capitalizing on this special structure, we designed novel nitroimidazole–oxime hybrids through the strategic conjugation of nitroimidazole scaffolds with oxime functionalities.

(5) An aromatic structure, such as phenyl and indole rings, is a common feature in endogenously bioactive molecules, and the conjugation of an electron-rich indole fragment with the nitroimidazole enone extends the π-π-conjugated molecular skeleton, potentially enhancing DNA-targeting capacity [51]. These characteristics establish them as a critical tool in modern pharmaceutical research, offering a powerful mechanism for creating more sophisticated and targeted therapeutic molecules [52]. Various substituents in the indole and benzyl groups will be investigated, as they may influence electron cloud distribution, thereby modulating physicochemical properties, altering target-binding affinity, and improving the membrane permeability of target molecules [53].

(6) The hydroxyphenyl fragment is widely present in various antiseptics, such as lysol, thymol, and so on. It has been demonstrated that the hydroxyl group can contribute to improving water solubility and enhancing affinity with biological targets via hydrogen bonds [54]. Herein, the terminal methyl group was replaced by the hydroxyphenyl group to explore its effect on antibacterial activity.

Accordingly, a series of novel heteroaryl bishydrazono nitroimidazoles and their analogs were constructed by hybridizing multiple components with nitroimidazole, and various substituents were introduced by diverse linkers to investigate the effect on antibacterial activities (Figure 2). The structures of newly synthesized compounds were confirmed by ^1^H NMR, ^13^C NMR, and HRMS spectra. We assessed the antibacterial activities of all the target compounds. The medicinal potential and mechanism of highly active compounds were evaluated through drug resistance studies, cytotoxicity assays, hemolysis tests, metabolic activity, antibiofilm assays, intracellular ROS level, DNA binding assays, and molecular docking.

## 2. Results and Discussion

### 2.1. Chemistry

**Scheme 1:**
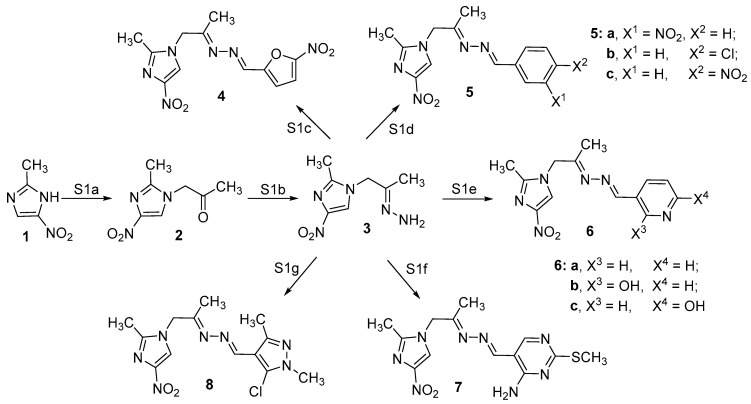
Preparation of furyl bishydrazono nitroimidazole **4**, phenyl bishydrazono nitroimidazole **5**, pyridinyl bishydrazono nitroimidazole **6**, pyrimidinyl bishydrazono nitroimidazole **7**, and pyrazolyl bishydrazono nitroimidazole **8**.

The target new heteroaryl bishydrazono nitroimidazoles and their analogs were prepared according to the synthetic route presented in the following schemes. Commercially available 2-methyl-5-nitro-1*H*-imidazole **1** was reacted with chloroacetone in the presence of potassium carbonate to afford acetonyl nitroimidazole **2** in high yield (Scheme 1). Acetonyl nitroimidazole **2** was treated with hydrazine hydrate in ethanol at room temperature to produce hydrazineyl nitroimidazole **3** in high yield in Scheme 1. To explore the effect of steric hindrance and electronic properties on antibacterial potencies, the desired furyl bishydrazono nitroimidazole **4**, phenyl bishydrazono nitroimidazoles **5a**–**c**, pyridinyl bishydrazono nitroimidazole **6**, pyrimidinyl bishydrazono nitroimidazole **7**, and pyrazolyl bishydrazono nitroimidazole **8** in Scheme 1 were developed by the corresponding aldehydes in moderate yields using acetic acid as a catalyst.

**Scheme 2:**
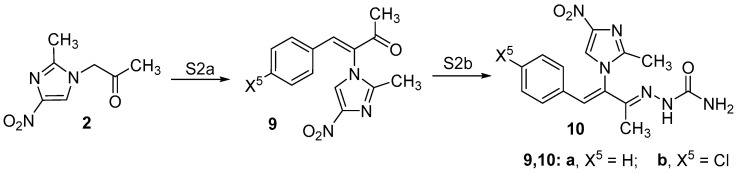
Preparation of phenyl-conjugated hydrazono nitroimidazole **10**.

**Scheme 3:**
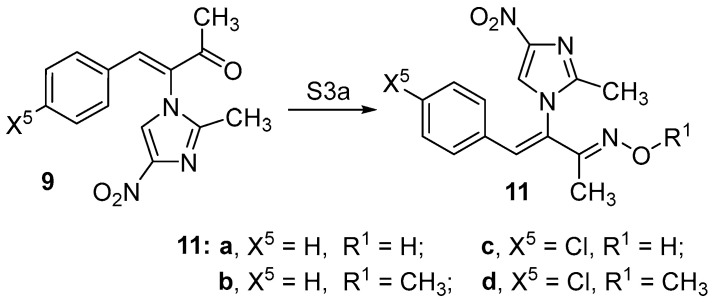
Preparation of phenyl-conjugated oxime–nitroimidazole **11**.

The bishydrazono group was changed to the α, β-unsaturated hydrazono moiety, and different aromatic structures were introduced to nitroimidazole to explore the influence of antibacterial activity. The condensation reaction of acetonyl nitroimidazole **2** with benzaldehyde or 4-chlorobenzaldehyde in refluxing toluene catalyzed by piperidine and acetic acid was used to produce phenylenone nitroimidazoles **9a**–**b**, which were further conjugated with semicarbazide in the presence of acetic acid to generate the phenyl hydrozono nitroimidazoles **10a**–**b** in moderate yields (Scheme 2). Meanwhile, a similar process was carried out through replacing semicarbazide with hydroxylamine hydrochloride or methoxyammonium chloride in refluxing ethanol, and the phenyl-conjugated oxime-nitroimidazoles **11a**–**d** (Scheme 3) were obtained in moderate yields.

Reagents and conditions: (S1a) chloroacetone, potassium carbonate, acetonitrile, 80 °C; (S1b) hydrazinium hydroxide solution, ethanol, room temperature; (S1c) 5-nitrofurfural, acetic acid, ethanol, 80 °C; (S1d) benzaldehydes, acetic acid, ethanol, 80 °C; (S1e) 3-pyridinecarboxaldehydes, acetic acid, ethanol, 80 °C; (S1f) 4-amino-2-(methylthio)pyrimidine-5-carbaldehyde, acetic acid, ethanol, 80 °C; (S1g) 5-chloro-1,3-dimethyl-1h-pyrazole-4-carbaldehyde, acetic acid, ethanol, 80 °C. The desired compounds were obtained in 45–65% yields.

Reagents and conditions: (S2a) benzaldehyde or 4-chlorobenzaldehyde, piperidine, acetic acid, toluene, 110 °C; (S2b) semicarbazide, acetic acid, ethanol, 80 °C. The desired compounds were obtained in 40–50% yields.

**Scheme 4:**
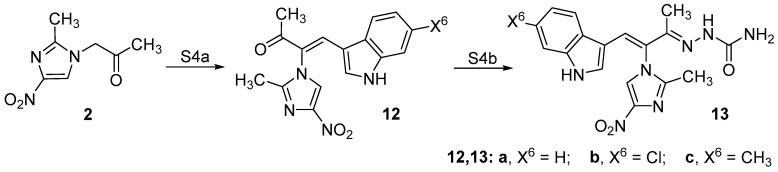
Preparation of indolyl-conjugated hydrazono nitroimidazole **13**.

**Scheme 5:**
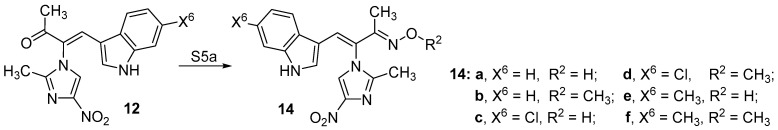
Preparation of indolyl-conjugated oxime–nitroimidazole **14**.

In order to enlarge the conjugated system, indole-3-carboxaldehydes were reacted with acetonyl nitroimidazole **2** to produce an important indolylenone nitroimidazole **12**, which was then condensed with semicarbazide to generate indolyl-conjugated hydrazono nitroimidazoles **13a**–**c** (Scheme 4), and hydroxylamine hydrochloride or methoxyammonium was reacted with indolylenone nitroimidazole **12** to afford indolyl-conjugated oxime–nitroimidazoles **14a**–**f** (Scheme 5) in moderate yields.

Reagents and conditions: (S3a) hydroxylamine hydrochloride or methoxyammonium chloride, acetic acid, ethanol, 80 °C. The desired compounds were obtained in 43–48% yields.

Reagents and conditions: (S4a) indole-3-carboxaldehyde or 6-chloroindole-3-carboxaldehyde or 6-methylindole-3-carboxaldehyde, piperidine, acetic acid, toluene, 110 °C; (S4b) semicarbazide, acetic acid, ethanol, 80 °C. The desired compounds were obtained in 51–54% yields.

Reagents and conditions: (S5a) hydroxylamine hydrochloride or methoxyammonium chloride, acetic acid, ethanol, 80 °C. The desired compounds were obtained in 47–56% yields.

**Scheme 6:**
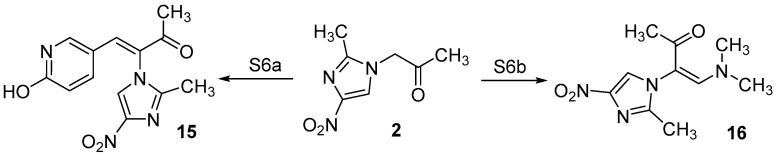
Preparation of hydroxypyridinyl enone nitroimidazole **15** and diaminoacetone nitroimidazole **16**.

In order to investigate the effect of a larger conjugated system on antibacterial activity, our next work turned toward the development of nitroimidazolylenone conjugates, because our previous work showed that the introduction of an enone moiety was helpful for improving antibacterial activity. Herein, 6-hydroxynicotinaldehyde was combined with acetonyl nitroimidazole **2** to afford hydroxypyridinyl enone nitroimidazole **15** in a moderate yield in Scheme 6. In the meantime, acetonyl nitroimidazole **2** was treated with *N*,*N*-dimethylformamide dimethyl acetal in the presence of triethylamine to give the diaminoacetone nitroimidazole **16** in a moderate yield.

**Scheme 7:**
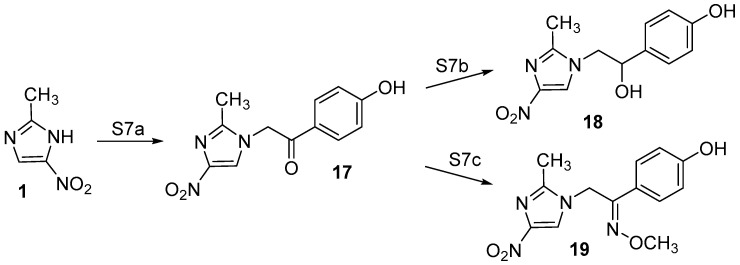
Preparation of hydroxyphenyl-conjugated nitroimidazoles **17**–**19**.

To obtain nitroimidazoles with more potent antibacterial activity, we further introduced a phenol moiety into nitroimidazole, because phenols represent a broad category of antimicrobial agents that can exert their bactericidal effects by denaturing proteins. Firstly, the substitution reaction between 2-methyl-5-nitro-1*H*-imidazole **1** and 2-bromo-4-hydroxyacetophenone with potassium carbonate as a deprotonating agent generated phenol-derived nitroimidazole **17** in a high yield (Scheme 7). Subsequently, the carbonyl group in compound **17** was reduced by sodium borohydride in methanol to produce hydroxyl nitroimidazole **18** in high yield. Finally, phenol-derived nitroimidazole **17** was reacted with methoxyammonium chloride in the presence of pyridine to afford nitroimidazole-derived phenol oxime **19** in a moderate yield.

Reagents and conditions: (S6a) 6-hydroxynicotinaldehyde, piperidine, ethanol, 80 °C; (S6b) *N*,*N*-dimethylformamide dimethyl acetal, triethylamine, ethanol, 80 °C. The desired compounds were obtained in 50–60% yields.

Reagents and conditions: (S7a) 2-bromo-4′-hydroxyacetophenone, potassium carbonate, acetonitrile, 80 °C; (S7b) sodium borohydride, methanol, 0 °C—room temperature; (S7c) methoxyammonium chloride, pyridine, ethanol, 60 °C. The desired compounds were obtained in 69–93% yields.

All the new compounds were analyzed for their structures by NMR and HRMS spectra. Moreover, the HRMS results were also consistent with the presented structures. Next, we determined the chemical structural configuration of the target compounds because the structural identification of isomers is beneficial for understanding their antimicrobial actions. We cultured and acquired a single crystal of representative phenyl-conjugated oxime-nitroimidazole **11b**, which was analyzed by X-ray diffraction (deposition number: 2505822). The result in Figure S1 and Tables S2–S8 shows that the deprotonation of 2-methyl-5-nitroimidazole in the presence of potassium carbonate resulted in the migration of the double bonds, leading to the dominance of the 2-methyl-4-nitroimidazole tautomer. Moreover, the enone moiety in the target compounds had a Z configuration, while the oxime part had an E configuration. All the results showed that the structures presented in the schemes were correct.

### 2.2. Antibacterial Activity

**Figure 3:**
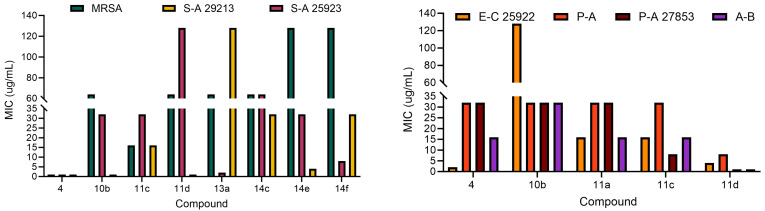
Antibacterial activity (MIC, μg/mL) for the active nitroimidazoles **4**, **10**, **11**, **13** and **14** ^a,b^. The experiment was conducted three times (*n* = 3) with consistent results. *^a^* MRSA, methicillin-resistant *Staphylococcus aureus*; *S-A* 25923, *Staphylococcus aureus* ATCC 25923; *S-A* 29213, *Staphylococcus aureus* ATCC 29213; *E-C*, *Escherichia coli*; *E-C* 25922, *Escherichia coli* ATCC 25922; *P-A*, *Pseudomonas aeruginosa*; *P-A* 27853, *Pseudomonas aeruginosa* ATCC 27853; *A-B*, *Acinetobacter baumannii*. *^b^* The number “>128” indicates that no inhibition was observed at the highest concentration tested (128 µg/mL).

The antibacterial activity of the prepared nitroimidazoles was evaluated against four Gram-positive bacteria and six Gram-negative bacteria, including drug-resistant bacteria, and clinically antibacterial norfloxacin was used as a positive control. The bacterial strains represent clinically significant pathogens and allow for direct comparison with other studies and established antibiotics. The results are given in Figure 3 and Table S1 as the minimum inhibitory concentration (MIC) determined by the broth microdilution method for microdilution plates. Three biological replicates (*n =* 3) were performed. Considering that hydrazone bridge bears two diverse nitrogen atoms and can bind to the bacterial membrane through electrostatic interaction to destroy the membrane integrity, the bishydrazineyl moiety was utilized to construct new nitroimidazoles. However, the data showed that bishydrazineyl derivative **3** displayed no antibacterial activity against most of the tested bacteria. We introduced the 5-nitrofurfural pharmacophore to compound **3**, resulting in derivative **4**, and we observed exceptional antibacterial activity of **4** against MRSA, *E-F*, *S-A* ATCC 25923, and *S-A* ATCC 29213, with MIC values ranging from 1 to 2 μg/mL. Moreover, compound **4** exhibited impressive antibacterial activity against Gram-negative bacteria, including *K-P*, *E-C*, and *E-C* ATCC 25922. The MIC values of 0.5, 2, and 2 μg/mL not only demonstrated potent activity but also surpassed the performance of the standard antibiotic norfloxacin. The broad inhibition spectrum across these diverse bacterial strains comprehensively illustrates the extensive antibacterial potential of compound **4**. Then, we continued our research by synthesizing the phenyl(bishydrazono) series **5a**–**c** and found that **5a** and **5c** had enhanced bacteriostatic activity compared to the others, emphasizing the importance of the nitro substituent. On the contrary, when we introduced multiple dihydrazone-based heterocyclic rings to obtain **6**–**8**, there was no obvious antibacterial activity against most of the tested bacteria.

Our next antibacterial evaluation was performed for the prepared series of hydrazono nitroimidazoles **10** and **13**. Figure 3 and Table S1 show that chlorophenyl hydrazono nitroimidazole **10b** exhibited high antibacterial effect toward *S-A* ATCC 29213 with a low MIC of 1 µg/mL, and this effect was 2-fold more active than that of norfloxacin. Moreover, this compound was also found to show moderate antibacterial activity (MIC = 32 µg/mL) against the tested Gram-negative bacteria, except for *K-P* and *E-C* ATCC 25922. Indolyl-conjugated hydrazono nitroimidazole **13a** could effectively suppress the growth of *S-A* ATCC 25923 at a low concentration of 2 µg/mL; meanwhile, compound **13a** exhibited moderate antibacterial activity against *K-P* and *E-C* 25922 with MIC values of 8 and 16 µg/mL, respectively. The introduction of a chlorine atom into the indole part resulted in improved antibacterial activity against most of the tested strains. Remarkably, chloroindolyl hydrazono nitroimidazole **13b** displayed moderate antibacterial potency toward MRSA, *E-F*, and *P-A* with an MIC of 16 µg/mL. Methylindolyl indolyl-conjugated hydrazono nitroimidazole **13c** exhibited good anti-*K-P* efficacy with a low MIC of 2 µg/mL, and this effect was 2-fold more active than norfloxacin.

Afterwards, we tried to introduce oximes to explore nitroimidazoles with stronger antibacterial potency based on the above antibacterial evaluation. We found that oxime–nitroimidazoles **11a**–**d** and **14a**–**f** exhibited poor antibacterial activity against the tested strains. Phenyl-conjugated oxime–nitroimidazole **11a** showed moderate antibacterial potency toward *S-A* 25923, *E-C* 25922, and *A-B* with MIC values of 16 µg/mL, while the formation of methyl oxime (**11b**) decreased antibacterial activity. Remarkably, chlorophenyl-conjugated oxime–nitroimidazole **11d** presented high antibacterial activity against *S-A* 29213 and *P-A* ATCC 27853 with low MIC values of 1 µg/mL. In particular, compound **11d** exhibited potent anti-*A-B* potency with a low MIC of 1 µg/mL, and this efficacy was 8-fold more active than norfloxacin. Indolyl-conjugated oxime-nitroimidazoles **14a** and **14b** without substituents on the indole ring showed low or no antibacterial potency against the tested strains, while the introduction of substituents on the indole ring was helpful for improving the antibacterial effect. Chloroindolyl-conjugated methyl oxime-nitroimidazole **14d** exhibited anti-MRSA activity comparable to norfloxacin (MIC = 8 µg/mL). Methylindolyl-conjugated oxime–nitroimidazole **14e** could effectively inhibit the growth of *S-A* 29213 and *P-A* 27853 at concentrations of 4 and 8 µg/mL, respectively; the antibacterial activity was enhanced when it was changed into methyl oxime. Remarkably, compound **14f** showed good anti-*K-P* potency with an MIC value of 8 µg/mL, and this effect was much higher than that of methylindolyl-conjugated oxime-nitroimidazole **14e** (MIC = 64 µg/mL).

Further bioactivity showed that hydroxypyridinyl enone nitroimidazole **15** exhibited remarkable antibacterial activity toward MRSA with a low MIC of 4 µg/mL, and this effect was 2-fold more active than that of norfloxacin. However, the formation of enaminone **16** did not improve antibacterial activity. We subsequently evaluated the phenol-based nitroimidazoles (**17**–**19**) and found them to be largely inactive against the tested bacterial strains.

### 2.3. Hemolytic Study

**Figure 4:**
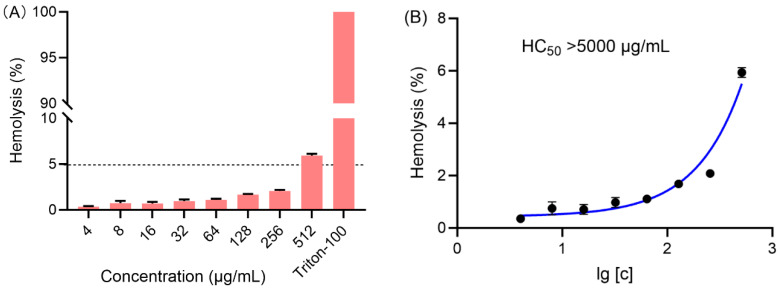
(**A**) Hemolysis of the highly active compound **4** at different concentrations. (**B**) HC_50_ (Required concentration for the hemolysis of 50%) of the highly active compound **4**. The experiment was conducted three independent replicates (*n* = 3).

Hemolysis assessment is commonly used to evaluate the toxicity of drugs to blood cells, with a low hemolysis rate serving as a crucial indicator of drug safety [55,56]. The hemolytic effect of active nitrofuryl bishydrozono nitroimidazole **4** on human red blood cells (RBCs) was tested using Triton X-100 as a positive control. The hemolysis percentage determined by the microdilution method showed that the highly active compound **4** exhibited hemolytic effects at a concentration of 512 μg·mL^−1^, with the hemolysis rate remaining below 5% at 256 μg·mL^−1^ (Figure 4). The low level of hemolytic activity indicated that the designed antimicrobial molecule **4** was unlikely to cause significant damage to red blood cells, suggesting good safety and potential for further development.

### 2.4. Proliferation Activity Study

**Figure 5:**
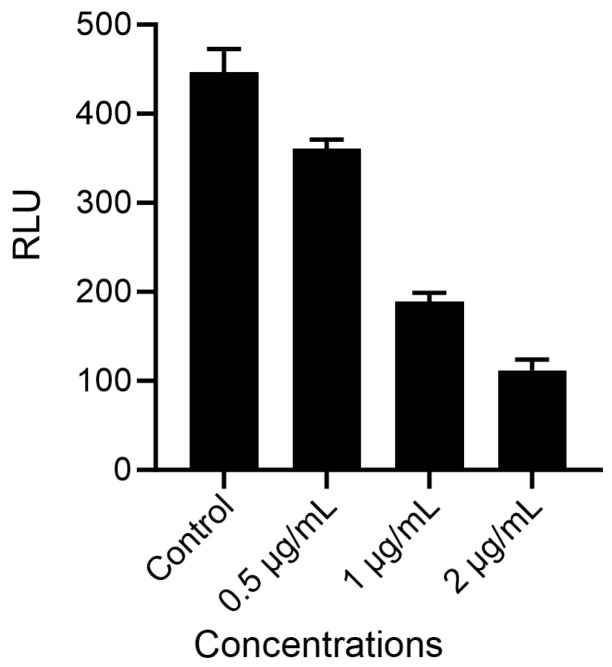
Proliferation inhibitory activity of MRSA treated with derivative **4**. The experiment was conducted with three independent replicates (*n* = 3).

After confirming the biosafety of active nitrofuryl bishydrozono nitroimidazole **4**, we further used the Alamar blue assay to estimate the proliferation inhibitory activity of compound **4** against MRSA. Metabolically active bacteria convert the indicator into red fluorescence, while damaged or inactive bacteria exhibit lower metabolic activity, resulting in weaker fluorescence signals. Thus, fluorescence intensity measured by a spectrofluorometer is proportional to the number of active bacteria. As shown in Figure 5, the untreated-MRSA group exhibited high fluorescence intensity. However, fluorescence values decreased progressively with increasing doses of compound **4** (from 0.5 µg/mL to 2 µg/mL), indicating a reduction in MRSA activity. The inhibitory efficiency was directly proportional to compound concentration. Cytotoxicity analysis previously indicated that compound **4** exhibited no significant toxicity at treating concentrations below 6.25 µg/mL (>2 µg/mL), suggesting that active molecule **4** could inhibit the proliferation and reduce the metabolic activity of MRSA (0.5 µg/mL) by interacting with it through various mechanisms.

### 2.5. Bacterial Resistance Study

**Figure 6:**
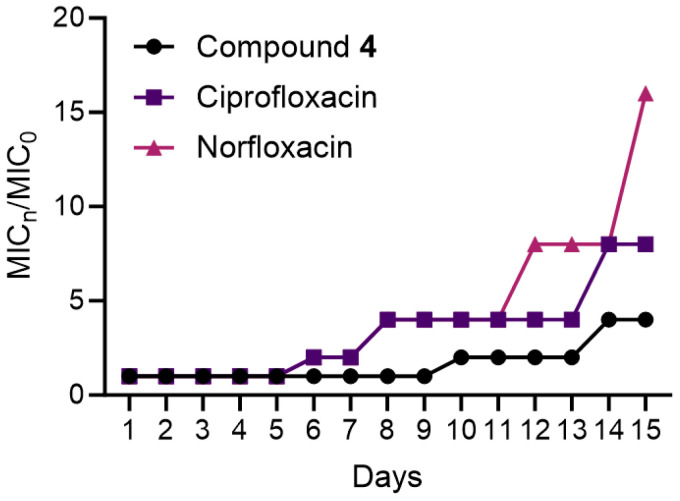
Resistant evolution of MRSA treated with compound **4**, norfloxacin, and ciprofloxacin. The experiment was conducted with three independent replicates (*n* = 3).

Bacterial resistance is a primary cause of treatment failure in bacterial infectious diseases [57,58]. The overuse of antibiotics and bacterial genetic variations have significantly increased the frequency of drug resistance, including multidrug resistance. Developing efficient and low-toxicity strategies to overcome bacterial resistance has become a major focus in the treatment of bacterial infections [59]. A subsequent experiment was conducted to evaluate the potential of nitrofuryl bishydrozono nitroimidazole **4** to reduce bacterial resistance with norfloxacin and ciprofloxacin as controls. As shown in Figure 6, a low tendency for resistance was observed with compound **4** against MRSA up to the eighth day, with mild resistance emerging on the ninth day. In contrast, MRSA began to develop resistance to norfloxacin and ciprofloxacin by the fifth day, which subsequently progressed rapidly, resulting in a 2-fold and 4-fold increase in MIC for ciprofloxacin and norfloxacin in 15 days. The results revealed that compound **4** could serve as an antibacterial candidate to resist bacterial resistance.

### 2.6. Drug Combination Effect

Drug combination is an essential strategy for treating complex diseases. It offers the potential to expand the antimicrobial spectrum, improve therapeutic efficacy, and reduce toxic reactions associated with single-drug use [60,61]. The results in Table 1 indicated that combining nitrofuryl bishydrozono nitroimidazole **4** with norfloxacin or oxacillin sodium enhanced its antibacterial properties, with FICI values of 0.5 and 0.25, respectively, suggesting a synergistic effect against MRSA. Notably, the combination of compound **4** with oxacillin sodium exhibited a stronger synergistic inhibitory effect.

### 2.7. Antibiofilm Assay

**Figure 7:**
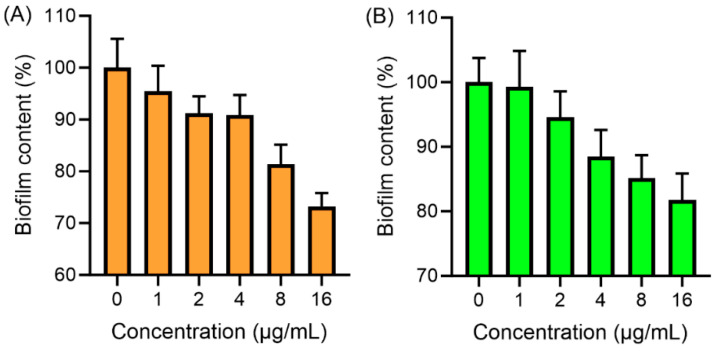
Biofilm inhibition (**A**) and eradication (**B**) of the highly active compound **4**. The experiment was conducted with three independent replicates (*n* = 3).

Bacterial biofilms promote the secretion of large amounts of extracellular polymeric substances (EPSs), which contribute to high antibiotic resistance [62,63]. Bacteria within biofilms exhibit antibiotic resistance that is several orders of magnitude higher than that of planktonic bacteria. Additionally, over 80% of severe clinical infections are associated with bacterial biofilm resistance [64]. Therefore, we evaluated the effect of active nitrofuryl bishydrozono nitroimidazole **4** on the disruption of MRSA biofilms. Figure 7 shows that active compound **4** could destroy biofilms of MRSA in a dose-dependent manner and significantly reduced the content of MRSA biofilms by 27% at 16 µg/mL, while the eradication rate of **4** against MRSA biofilms was 19%, making compound **4** a potential candidate for impeding the formation of pathogenic biofilms and reducing bacterial resistance development.

### 2.8. Membrane Permeability and Depolarization

**Figure 8:**
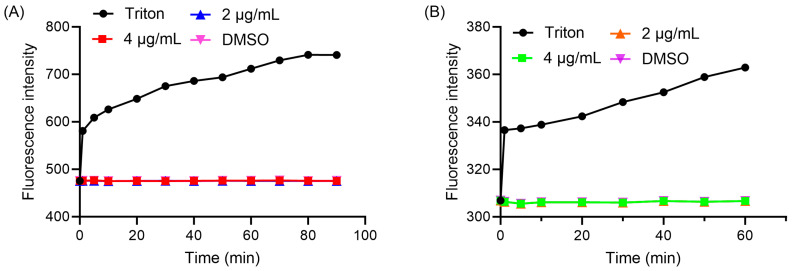
Membrane permeabilization (**A**) and depolarization (**B**) of MRSA in the presence of compound **4**. The experiment was conducted with three independent replicates (*n* = 3).

To further investigate the impact of active compound **4** on cell membranes, we examined changes in the cellular membrane morphological structure of MRSA. Cells treated with the positive control Triton X-100 showed a significant increase in fluorescence intensity within 60 min, indicating membrane rupture and depolarization [65,66,67]. In contrast, cells treated with different concentrations of compound **4** exhibited no notable changes in fluorescence intensity (around 470 nm) compared to the negative control group (Figure 8A). Using the membrane fluorescent probe DiSC3(5), we found that the cell membrane potential after stimulation of compound **4** remained stable at 300 nm over 60 min, which was lower than the positive control treatment group (360 nm) and comparable to the negative control (DMSO) (Figure 8B). These findings indicated that compound **4** could not alter membrane permeability through interaction with lipopolysaccharides or lipoproteins or induce membrane potential collapse through ion channel disruption. Instead, it was more likely to damage the cell membrane indirectly by modulating intracellular signaling pathways or metabolic processes, which distinguishes compound **4** from more aggressive membrane-targeting agents.

### 2.9. Intracellular ROS Level

**Figure 9:**
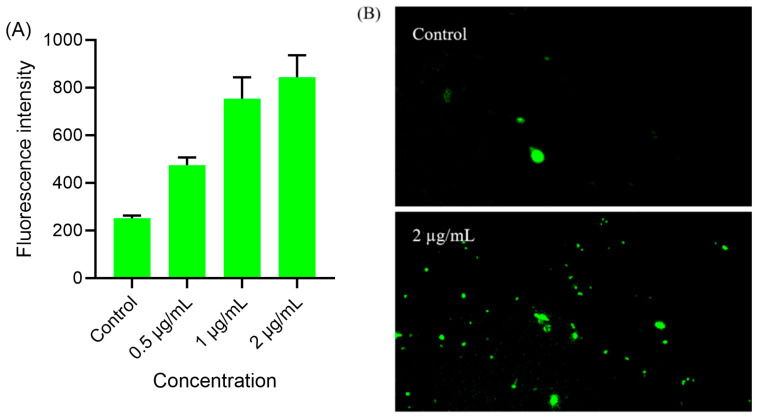
(**A**) ROS production in MRSA treated by the highly active bishydrazone nitroimidazole **4** at different concentrations. (**B**) Fluorescence micrographs of MRSA with DCFH-DA staining treated by the highly active compound **4** at 2 µg/mL for 6 h (400× magnification), with DMSO was used as a control. The experiment was conducted with three independent replicates (*n* = 3).

Reactive oxygen species are primarily generated in the cell membrane of bacteria, where excessive ROS can react with key biological macromolecules inside the cell, causing cytotoxicity and disrupting bacterial survival [68,69]. We used a fluorescence assay based on 2′,7′-dichlorofluorescin diacetate (DCFH-DA) to measure the levels of intracellular ROS. DCFH-DA is non-fluorescent but is oxidized by ROS to form the green fluorescent compound dichlorofluorescein (DCF) [70,71,72]. The intensity of green fluorescence is proportional to intracellular ROS levels. As shown in Figure 9, treatment of MRSA with varying concentrations of nitrofuryl bishydrozono nitroimidazole **4** led to a gradual increase in fluorescence density within the bacteria. At a concentration of 2 µg/mL, fluorescence density was four times higher than that of the control group, with strong green fluorescence visible under a fluorescence microscope. This suggested that compound **4** induced excessive ROS production in the bacterial membrane, leading to oxidative damage and enhancing the inhibitory effect.

### 2.10. Interaction of Compound **4** with DNA 

**Figure 10:**
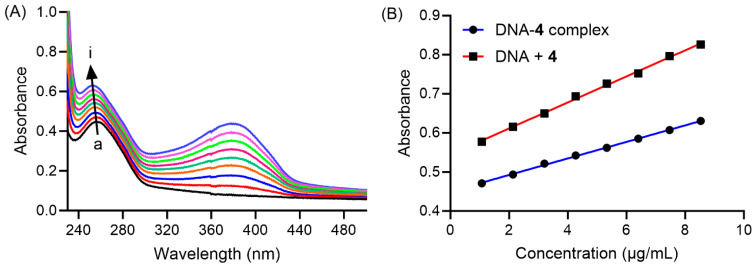
(**A**) Ultraviolet spectra (the arrow a − i) of DNA with increasing concentration (colored lines from black to blue) of highly active compound **4**. (**B**) Absorption comparison at 260 nm between the **4**−DNA complex and the sum of DNA and compound **4**. The experiment was conducted with three independent replicates (*n* = 3).

DNA is a crucial genetic material in living organisms, and studying the interactions between drugs and bacterial DNA is essential for understanding the pathogenesis of certain diseases and the therapeutic mechanisms of drugs [73,74]. UV-vis spectroscopy is the most commonly used and convenient technique to investigate the interaction mechanisms between small molecules and nucleic acids [75]. We recorded the UV−vis absorption spectra of increasing amounts of compound **4** and a fixed concentration of DNA. As shown in Figure 10, the characteristic absorption peak of DNA at 260 nm shifted to longer wavelengths with increasing concentrations of compound **4** (0 − 0.7 × 10^−13^ mM), while the DNA concentration was fixed at c (DNA) = 0.568 × 10^−13^ mM. Additionally, the absorbance of the compound **4**–DNA complex was higher than the sum of the absorbances of the free DNA and compound **4**, suggesting a chromogenic enhancement during the interaction. This enhancement was likely due to the intercalative or electrostatic binding of compound **4** to DNA, resulting in DNA denaturation and structural disruption.

**Figure 11:**
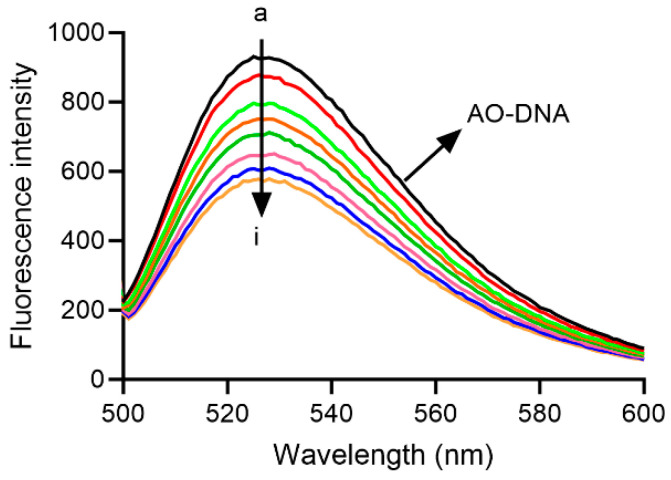
Competitive effect between the active molecule **4** and the AO-DNA system. The fluorescence intensity at 537 nm (the arrow a − i) decreased with the increasing concentration (colored lines from orange to black) of nitroimidazole **4**.

We subsequently used acridine orange (AO), a cationic dye, as a spectral probe to further confirm the interaction mode between the compound and DNA [76]. As shown in Figure 11, the fluorescence intensity at 537 nm significantly decreased with the increasing concentrations of furan nitroimidazole **4**, further confirming that it could displace the AO in the DNA-AO complex, thereby intercalating into the DNA.

As a complementary in silico analysis, molecular docking was performed to explore other potential targets; it suggested a potential affinity for DNA topoisomerase (Figure S3). However, the primary focus and more substantiated mechanism based on our experimental data is its interaction with DNA. The antibacterial selectivity of compound **4** might be primarily attributed to two key factors: disrupting the exposed bacterial nucleoid without harming chromatin-protected mammalian DNA, aided by its preferential accumulation in bacterial cells [77,78], and exploiting fundamental structural differences specifically in DNA organization and ribosome architecture (70S in bacteria and 80S in eukaryotes), thus selectively inhibiting bacterial processes with minimal impact on human cells [79,80].

## 3. Materials and Methods

### 3.1. General Methods and Reagents

All initial chemicals and reagents were commercial and used without further purification. The bacterial strains used in this study were from Southwest hospital. H1299 and Hela cells were obtained from the Shanghai Institute of Cell Biology, Chinese Academy of Sciences, Shanghai, China. The Cell Counting Kit-8 (CCK-8) was from MeilunBio (Dalian, China). Fresh human blood used in the experiment was purchased from iphasebio (Suzhou, China). The blood provided by the company is collected from qualified healthy donors, and all collection processes strictly comply with relevant ethical norms and regulations, ensuring that the donors have signed informed consent forms. Crystal violet and calf thymus DNA were from Solarbio (Beijing, China). Propidium iodide (PI), 3,3′-dipropylthiadicarbocyanine iodide [diSC3(5)], dichlorofluorescein diacetate (DCFH-DA), acridine orange (AO), and N-acetyl-L-cysteine (NAC) were from Beyotime (Shanghai, China). Resazurin sodium salt was from Sigma-Aldrich (St. Louis, MO, USA). The antibiotics (norfloxacin, ciprofloxacin, and oxacillin sodium monohydrate) were obtained from HEOWNS (Tianjin, China). A TU-2450 spectrophotometer (Puxi Analytic Instrument Ltd., Beijing, China) and an F-7000 spectrofluorometer (5J1-0004 model, Hitachi, Tokyo, Japan) were utilized to record UV spectra and fluorescence spectra, respectively. NMR, HRMS spectra and detailed steps of the experimental protocols can be found in the Supporting Information. All experiments were performed in triplicate unless otherwise stated.

### 3.2. Biological Assays

#### 3.2.1. Antibacterial Assay

The minimum inhibitory concentration (MIC) values of the prepared nitroimidazolylenone derivatives were determined using the broth microdilution method according to Clinical and Laboratory Standards Institute (CLSI) guidelines. Bacterial suspensions were standardized in sterile saline to a concentration of 10^4^ colony-forming units per milliliter (CFU/mL). Stock solutions of the test compounds and reference drugs were prepared by dissolving them in dimethyl sulfoxide (DMSO). These stock solutions were then serially diluted in Mueller–Hinton broth (Guangdong Huaikai Microbial Science & Technology Co., Ltd., Guangzhou, China) to generate a concentration range of 256, 128, 64, 32, 16, 8, 4, 2, 1, 0.5, and 0.25 μg/mL. Aliquots of 100 μL of these diluted solutions were dispensed into the wells of a 96-well plate, followed by the addition of 100 μL of bacterial suspension (10^4^ CFU/mL). The plates were then incubated at 37 °C for 24 h. The antibacterial activity was evaluated by determining the minimum inhibitory concentration (MIC), which represents the lowest concentration of a compound that prevents visible bacterial growth.

#### 3.2.2. Cytotoxicity and Hemolytic Activity

Cytotoxicity: The cytotoxicity of the active compound **4** was investigated using human large-cell lung cancer cell line H1299 and human cervical carcinoma cell line HeLa. Cells were seeded at approximately 7000 cells per well and cultured in Dulbecco’s modified Eagle’s medium (DMEM) supplemented with 10% fetal bovine serum (FBS) (Corning, New York, NY, USA) and 1% penicillin–streptomycin. The cultures were maintained at 37 °C for 24 h, with cells exposed to varying concentrations of compound **4** or a vehicle control (DMSO). Cell viability was assessed using the Cell Counting Kit-8 (CCK-8) assay. The percentage of viable cells in the compound-treated groups was calculated relative to the DMSO vehicle control groups, providing a quantitative measure of the compound’s cytotoxic potential.

Hemolytic activity: Compound **4** was dissolved in DMSO (1 mL) and diluted in normal saline to prepare the analyte liquor (512 μg/mL). The above analyte liquor (500 μL) was added to the first centrifuge tube, and a double dilution method was then employed to generate a concentration gradient of 256, 128, 64, 32, 16, 8, 4, and 2 μg/mL [normal saline (500 μL) was pre-added into every tube]. Fresh human blood was centrifuged at 5000 rpm for 5 min and washed three times with normal saline to isolate erythrocytes. The red blood cells were then diluted to a 5% suspension in the same medium. Aliquots of 500 μL of the erythrocyte suspension were transferred to individual tubes, with the final volume adjusted to 1 mL. Normal saline served as the negative control, while 0.1% Triton X-100 in normal saline was used as the positive control. These tubes were incubated for different durations (1, 3 and 6 h) at 37 °C and then centrifuged at 5000 rpm for 5 min. The hemolysis photos for the 3 and 6 h treatments were taken in the sunlight. Then, 100 μL of liquid supernatant was transferred into a 96-well plate and the OD_540_ values were determined on a microplate reader. The bottom cell suspension (5 μL) treated with the active compound **4** for 3 h was drop-casted on a glass slide and observed under a microscope. The hemolysis rate was calculated by the following equation:

(1) $$\mathrm{Hemolysis}\;\mathrm{ratio}\;\left(\%\right)=\frac{\mathrm{ODsample}-\mathrm{ODsaline}}{\mathrm{ODtriton}-\mathrm{ODsaline}}\;\times \;100\%$$

The process was repeated three times.

#### 3.2.3. Proliferation Activity

Pre-cultured MRSA were exposed to different concentrations (1/2, 1 and 2 μg/mL) of the active compound **4** for 6 h at 37 °C. The treated bacteria were centrifuged at 8000 rpm for 5 min, washed, and resuspended with a mixture buffer composed of 5 mM HEPES and 5 mM glucose (1:1 *v*/*v*). An aqueous solution of resazurin sodium salt (50 μg/mL, 10 μL) was added to each well, and the plate was incubated at 37 °C for 15 min in dark. Metabolic activity was assessed by measuring fluorescent intensity at 570 nm (OD_570_) using a microplate reader. The results were expressed as the percentage of metabolic activity relative to untreated bacteria. The picture of bacterial viabilities in resazurin sodium salt was taken in the sunlight after the dyed liquid was transferred into a centrifuge tube.

#### 3.2.4. Resistance Study

Bacterial suspensions containing a sub-minimum inhibitory concentration (sub-MIC) of derivative **4** and reference drugs were collected and sub-cultured following the initial MIC determination. The MIC values of compound **4** and reference drugs were determined against each successive passage of MRSA over a 15-day period. The final MRSA passage was generated through continuous treatment with norfloxacin for 15 generations. Subsequently, the antibacterial activity of compound **4**, norfloxacin, and ciprofloxacin was assessed using the previously described methodology.

#### 3.2.5. Drug Combination

The FICI was determined through the standard checkerboard assay in a 96-well plate. Oxacillin sodium monohydrate was serially diluted along the abscissa in Mueller–Hinton broth, while the active compound **4** was diluted along the ordinate to create a 10 × 7 matrix. The bacterial culture was prepared in Mueller–Hinton broth as previously described for the evaluation of antimicrobial activities. The plate was incubated for 18 h at 37 °C after the bacterial suspension was inoculated into each well.

#### 3.2.6. Membrane-Disturbing Activity

Membrane permeability assay: Pre-cultured MRSA were harvested by centrifugation at 8000 rpm for 5 min, then washed and resuspended in a mixture buffer consisting of equal volumes of 5 mM HEPES and 5 mM glucose. The active compound **4** at a concentration of 1× minimum inhibitory concentration (MIC) was added to the MRSA suspension (2 mL), which also contained propidium iodide (PI, 50 μM, 200 μL). The suspension was then incubated at 37 °C for 90 min. The biomass was collected by centrifugation at 8000 rpm for 5 min, washed with phosphate-buffered saline (PBS) to remove unbound PI, and resuspended again in the original mixture buffer. Fluorescence spectroscopy was conducted using a fluorescence spectrometer, with an excitation wavelength of 535 nm and an emission wavelength of 617 nm. Dimethyl sulfoxide (DMSO) and 1% Triton X-100 served as negative and positive controls, respectively. The uptake of propidium iodide was monitored at 10 min intervals over a 90 min period.

Depolarization of cytoplasmic membrane: MRSA were harvested by centrifugation at 8000 rpm for 5 min and washed with a buffer mixture of 5 mM HEPES and 5 mM glucose (1:1 *v*/*v*), and the OD_600_ of the resuspended particles was adjusted to approximately 0.1. The bacterial suspension was supplemented with 3,3′-dipropylthiadicarbocyanine iodide [diSC3(5)] dye (0.4 mM) and incubated in darkness at 37 °C for 30 min. Subsequently, a potassium chloride (KCl) solution (100 mM) was added to equilibrate the membrane potential. After a 10 min equilibration period, the MRSA strain was treated with compound **4** at 1 μg/mL. Fluorescence spectroscopy was conducted with an excitation wavelength of 622 nm and an emission wavelength of 670 nm. Measurements were recorded periodically over a 10 min interval with DMSO and 1% Triton X-100 serving as negative and positive controls, respectively.

#### 3.2.7. Biofilm Inhibition and Eradication

The pre-cultured MRSA were washed and resuspend in Mueller–Hinton broth. The bacterial suspension was co-incubated with compound **4** at concentrations of 1, 2, 4, 8, and 16 μg/mL for 24 h. The formed biofilms were collected by centrifugation at 8000 rpm for 5 min and washed three times with phosphate-buffered saline (PBS) to remove unbound bacterial cells. Crystal violet solution (100 μL per well) was then added, and the plate was incubated for 30 min at 37 °C in the dark. The biomass was subsequently centrifuged at 500 rpm for 10 min, repeatedly washed with PBS to remove excess crystal violet, and then solubilized in anhydrous ethanol (200 μL). The OD_595_ values were read on a microplate reader, and the inhibition (%) of biofilm formation was calculated in comparison to the untreated group. The assessment was conducted in triplicate and the data were present as average values. To assess biofilm eradication, MRSA were initially cultured for 24 h to establish mature adherent biofilms. The biofilm inhibition protocol was then repeated on these pre-formed biofilms. Representative images of the MRSA biofilms stained with crystal violet were captured under natural sunlight.

#### 3.2.8. Intracellular Oxidative Stress

Determination of ROS accumulation: MRSA were treated with increasing concentrations (1/2, 1 and 2 μg/mL) of compound **4** for 6 h at 37 °C, and 5 mM *N*-acetyl-*L*-cysteine (NAC) was added to the bacterial suspension in the presence of compound **4** at 1 μg/mL as the positive group. Afterwards, biomass was collected (8000 rpm, 5 min), washed with PBS three times, and resuspended in the same buffer. The further cultivation lasted for 30 min after adding the probe dichlorofluorescein diacetate (DCFH-DA, 100 μM). The fluorescence was measured with an excitation wavelength of 485 nm and emission wavelength of 528 nm after removing the unbound probe, and the fluorescence intensity was given as the means from three parallel experiments.

DCFH-DA staining: MRSA (about 1 × 10^5^ CFU/mL, 500 μL) was exposed to compound **4** at 1 μg/mL and DCFH-DA (100 μM, 100 μL) for 6 h at 37 °C. After removing excess stains (8000 rpm, 5 min), stained cells were fixed in 2.5% glutaraldehyde and resuspended in PBS (100 μL). Then, the suspension (5 μL) was drop-casted on a clean glass slide, airdried, and observed under a fluorescence microscope equipped with a blue laser.

#### 3.2.9. Interaction with DNA

Uv-vis absorption spectrum of DNA: The interaction between compound **4** and DNA was conducted with calf thymus DNA as a substrate. The UV absorption spectra of DNA at a fixed concentration of 1.56 × 10^−4^ mol/L with continuously increasing concentrations of compound **4** were recorded under each gradient with a UV-vis spectrophotometer (F-7000 spectrofluorometer (5J1-0004 model, Hitachi, Tokyo, Japan)). In the meantime, the absorption spectrum under a fixed concentration of compound **4** was monitored under each gradient with the addition of DNA.

Competition assay between active nitroimidazole 4 and AO with DNA: DNA was preincubated with acridine orange (AO) overnight at 4 °C. Active nitroimidazole **4** solutions in DMSO (1 mL) were incrementally added to a cuvette containing the AO-DNA system (3 mL). Fluorescence spectra were recorded after a 5 min equilibration period for each addition, using a fluorescence photometer set to an excitation wavelength of 490 nm and an emission wavelength of 530 nm.

#### 3.2.10. Molecular Docking

The crystal structure of DNA gyrase (PDB code: 2XCS) in pdb format was downloaded from Protein Data Bank. AutoDock 4.2.6 was used to perform docking with the ligand molecules. PyMOL 3.0 (3D) and Discovery Studio 4.5 Client (2D) were used to visualize the molecular interactions.

#### 3.2.11. X-Ray Crystallography

Single crystals of compound **11b** suitable for X-ray analysis were obtained by slow evaporation of a solution in dichloromethane. The single-crystal X-ray diffraction analysis was performed at Zhengzhou University. A suitable crystal was selected and placed on a Xcalibur, Eos, Gemini diffractometer. The crystal was kept at 293(2) K during data collection. Using Olex2, the structure was solved with the ShelXS structure solution program using Direct Methods and refined with the ShelXL refinement package using Least Squares minimization.

## 4. Conclusions

We successfully constructed a series of novel heteroaryl bishydrazono nitroimidazoles and their analogs using a fragment-based drug design strategy. Preliminary antimicrobial screening identified that some target nitroimidazole conjugates exhibited notable antibacterial properties. In particular, nitrofuryl bishydrozono nitroimidazole **4** distinguished itself by displaying a broad antibacterial spectrum. The highly active compound **4** exhibited a remarkable combination of therapeutic attributes, characterized by low cytotoxicity (Figure S2) and hemolysis. Moreover, compound **4** could effectively inhibit the proliferation of methicillin-resistant *Staphylococcus aureus* and reduce its metabolic activity while maintaining a notably low bacterial resistance potential. When combined with oxacillin sodium, derivative **4** displayed a potent synergistic inhibitory effect that further enhanced its antibacterial efficacy. Mechanistic investigations revealed that the highly active molecule **4** could obstruct pathogenic bacterial biofilm formation and induce excessive ROS production within bacterial membranes, it also had the ability to intercalate into DNA to cause structural denaturation and disruption. These diverse and targeted actions position nitrofuryl bishydrozono nitroimidazole **4** as a promising candidate in the ongoing battle against drug-resistant bacteria, which presents hope for confronting the escalating challenge of antimicrobial resistance. Future studies will focus on time–kill kinetics, post-antibiotic effects, and further optimization of physicochemical and pharmacokinetic properties to advance this class of molecules toward preclinical assessment.

## Figures and Tables

**Table 1:** Combination therapy of the highly active molecule **4** with norfloxacin and oxacillin sodium monohydrate *^b^*.

Bacteria	Norfloxacin		Oxacillin Sodium Monohydrate	
	FICI	Effect	FICI	Effect
MRSA	0.50	Synergism	0.25	Synergism

*^b^* FIC_a_ = MIC of the combination/MIC_a_ alone; FIC_b_ = MIC of the combination/MIC_b_ alone; _a_ = the highly active compound **4**; _b_ = norfloxacin or oxacillin sodium monohydrate; FICI = FIC_a_ + FIC_b_; FICI ≤ 0.5, synergism; 0.5 < FICI ≤ 1, additivity; 1 < FICI ≤ 2, indifference. The experiment was conducted with three independent replicates (*n* = 3).

## Data Availability

The original contributions presented in this study are included in the article/Supplementary Material. Further inquiries can be directed to the corresponding author(s).

## Supplementary Materials

The following supporting information can be downloaded at https://www.mdpi.com/article/10.3390/ijms262411836/s1.
