# Supplementary material for: Heteroaryl Bishydrazono Nitroimidazoles: A Unique Structural Skeleton with Potent Multitargeting Antibacterial Activity
Source: Int J Mol Sci. 2025 Dec 8;26(24):11836. doi: 10.3390/ijms262411836 (PMC12733231; doi:10.3390/ijms262411836)
Supplement: Supplementary file 1 [file ijms-26-11836-s001.zip › ijms-4000502-supplementary.pdf]

Supplementary Information

Title: Heteroaryl Bishydrazono Nitroimidazoles: A Unique Structural Skeleton with Potent Multitargeting Antibacterial Activity

1. Chemistry

The reagents and solvents involved in chemical and biological experiments were commercially available. Thin-layer chromatography (TLC) analysis was applied to reaction monitoring and silica gel (#300–400) was used for column chromatography. Melting points were measured with WRX-4 Melting-point Apparatus. ^{1}H spectra and ^{13}C NMR spectra were recorded on the Bruker AVANCE III 600 MHz or 400 MHz spectrometer. The high resolution mass spectra (HRMS) were performed on an Bruker impact II 10200 spectrometer and Waters Micromass Q-Tof Micro^{TM} mass spectrometers. The HPLC traces of target nitroimidazoles were analyzed using a HITACHI primaide HPLC (eluent A, acetonitrile; eluent B, water; elution ratio, 65.0% (A): 35.0% (B); retention time, 30 min; flow rate, 1 mL/min).

2. Analysis of X-ray diffraction

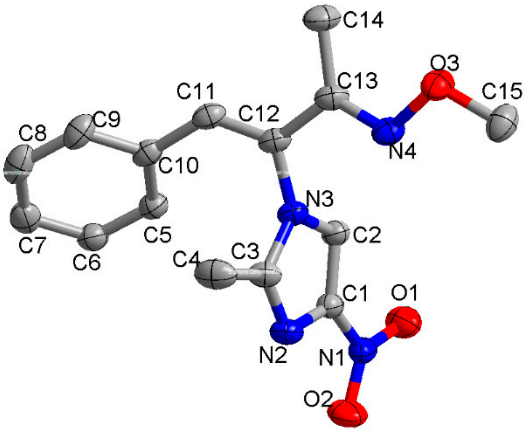

Figure S1. X-ray structure of phenyl-conjugated oxime-nitroimidazole **11b**.

3. Antibacterial activity

Table S1. Antibacterial activity (MIC, μg/mL) for the prepared nitroimidazoles **3–8** and **10–19**.[a,b]

Compounds	Gram-positive bacteria				Gram-negative bacteria					
	MRSA	*E-F*	*S-A* 25923	*S-A* 29213	*K-P*	*E-C*	*E-C* 25922	*P-A*	*P-A* 27853	*A-B*
**3**	>128	>128	>128	>128	>128	>128	>128	>128	128	>128
**4**	1	2	1	1	0.5	2	2	32	32	16

**5a**	64	128	32	>128	32	128	32	64	128	16
**5b**	>128	>128	128	128	>128	>128	>128	>128	>128	>128
**5c**	16	64	>128	128	32	16	128	32	>128	16
**6a**	>128	128	128	32	>128	64	>128	>128	>128	>128
**6b**	128	128	64	128	>128	128	>128	128	128	>128
**6c**	>128	128	>128	64	>128	128	128	128	>128	>128
**7**	128	>128	128	64	128	128	128	128	128	128
**8**	128	>128	>128	>128	>128	>128	>128	128	>128	>128
**10a**	>128	32	>128	>128	128	128	64	64	128	128
**10b**	64	64	32	1	64	32	128	32	32	32
**11a**	64	32	16	128	64	64	16	32	32	16
**11b**	>128	64	128	>128	128	128	128	128	>128	>128
**11c**	16	32	32	16	>128	>128	16	32	8	16
**11d**	64	128	128	1	>128	128	4	8	1	1
**13a**	64	128	2	>128	8	>128	16	128	>128	>128
**13b**	16	16	64	128	64	128	128	16	128	64
**13c**	>128	>128	16	64	2	128	64	>128	128	>128
**14a**	64	64	64	32	64	>128	16	64	128	128
**14b**	>128	128	16	128	32	>128	>128	>128	>128	>128
**14c**	32	16	64	>128	128	64	32	32	128	>128
**14d**	8	16	>128	>128	16	>128	>128	64	>128	>128
**14e**	>128	>128	32	4	64	>128	32	128	8	>128
**14f**	>128	>128	8	32	8	>128	64	128	64	>128
**15**	16	64	>128	128	>128	128	128	>128	128	128
**16**	>128	>128	>128	>128	>128	>128	>128	>128	128	>128
**17**	>128	>128	>128	>128	>128	>128	>128	>128	>128	>128
**18**	>128	>128	>128	>128	>128	>128	>128	>128	128	>128
**19**	>128	>128	>128	>128	>128	>128	>128	>128	128	>128
**Norfloxacin**	8	4	1	2	4	16	8	2	0.5	8

[a] MRSA, methicillin-resistant *Staphylococcus aureus*; *E-F*, *Enterococcus faecalis*; *S-A* 25923, *Staphylococcus aureus* ATCC 25923; *S-A* 29213, *Staphylococcus aureus* ATCC 29213; *K-P*, *Klebsiella pneumonia*; *E-C*, *Escherichia coli*; *E-C* 25922, *Escherichia coli* ATCC 25922; *P-A*, *Pseudomonas aeruginosa*; *P-A* 27853, *Pseudomonas aeruginosa* ATCC 27853; *A-B*, *Acinetobacter baumannii*.

[b] The number ">128" indicates that no inhibition was observed at the highest concentration tested (128 μg/mL).

4. Cytotoxicity assay

To ensure biosafety and assess the potential for clinical application, we also evaluated the cytotoxicity of the highly active nitrofuryl bishydrozono nitroimidazole **4** on the human large cell lung cancer cells H1299 and HeLa cell line by CCK-8 assays (Figure S2). Cell viability of H1299 remained 86% after treatment with preferred derivative **4** at 25 μg/mL, while the viable HeLa cells exceeded 83%. When the concentration was below 6.25 μg/mL, there was no significant toxicity to both cells, suggesting compound **4** showed low cytotoxicity, and made us aware that we could achieve a balance between antibacterial activity and cytotoxicity by controlling drug

concentration, thereby realize higher therapeutic efficacy in subsequent research.

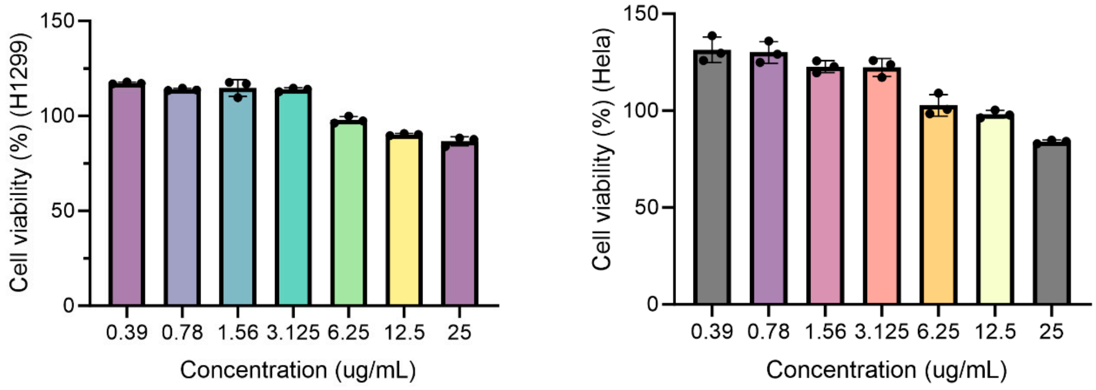

Figure S2. Cytotoxic effects of derivative **4** against human large cell lung cancer cells H1299 (left) and human cervical carcinoma cell line HeLa (right).

5. Molecular docking study

To further elucidate the molecular interaction pattern between compound **4** and DNA, molecular docking studies were performed using DNA isomerase (PDB code: 2XCS). As depicted in Figure S3, compound **4** demonstrated strong binding within the active pocket of DNA isomerase, exhibiting a binding energy of -5.97 kcal/mol. The structural analysis revealed that the nitro group of the nitroimidazole moiety in compound **4** formed two critical hydrogen bonds with amino acid residues Asn-1269 and Arg-1272, each with a distance of 1.8 Å. Furthermore, additional hydrogen bonds were established between the nitro group of the nitrofuran component and the important base pairs DG-18 of DNA. These multiple interaction points likely contributed to the formation of stable DNA-compound **4** supramolecular complexes.

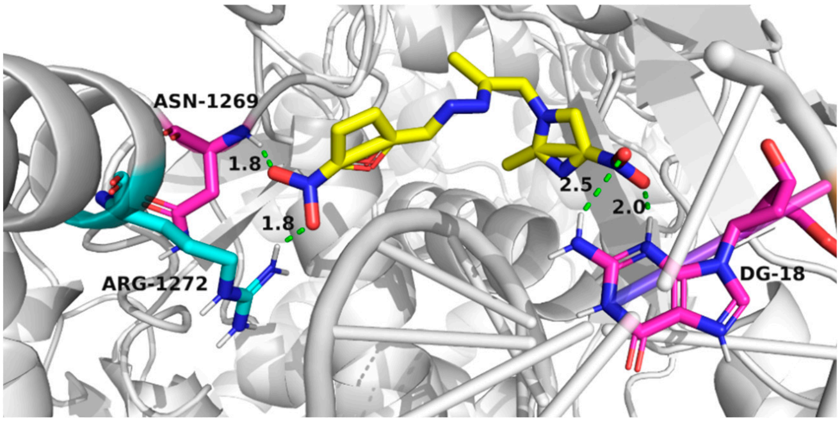

Figure S3. Molecular docking diagram of DNA (PDB: 2XCS) and compound **4**

6. Experimental protocols

6.1. Synthesis of intermediates and target compounds

*6.1.1. General synthesis of desired acetonyl imidazole **2**, phenylenone imidazoles **9a** and **9b**, and indolylenone imidazoles **12a–c***

Acetonyl imidazole **2**, phenylenone imidazoles **9a** and **9b**, and indolylenone imidazoles **12a–c** were prepared according to the previously reported method[1, 2], and the brief details of the methodology are as follows:

A mixture of 2-methyl-4-nitro-1H-imidazole (79 mmol) and potassium carbonate (157 mmol) was stirred in acetonitrile, then the 1-chloropropan-2-one (6.9 mL) was added to the mixture at 80 ℃ for 5 h to get target compound **2**.

A mixture of compound **2** (1.08 mmol), piperidine (0.011 mL) and glacial acetic acid (0.040 mL) was stirred in toluene at 45 ℃ for 0.5 h, then benzaldehyde (**9a**) or 4-chlorobenzaldehyde (**9b**) was added, and the resulting system was stirred at 120 ℃. After the reaction was completed, the mixture was cooled to room temperature and evaporated under reduced pressure, the residue was extracted with chloroform and the combined organic phase was dried over anhydrous sodium sulfate and further purified by silica gel column chromatography (eluent, chlorlform/acetone, 8/1, V/V) to give compound **9a** or **9b**.

A mixture of compound **2** (2.72 mmol), piperidine (0.027 mL) and glacial acetic acid (0.10 mL) was stirred in toluene at 45 ℃ for 0.5 h, then indole-3-carboxaldehyde (**12a**) or 6-chloroindole-3-carboxaldehyde (**12b**) or 6-methylindole-3-carboxaldehyde (**12c**) was added. Upon completion of the reaction, the mixture was evaporated under reduced pressure and cooled to room temperature, the residue was extracted with chloroform, and the combined organic phase was dried over anhydrous sodium sulfate and further purified by silica gel column chromatography (eluent, chlorlform/acetone, 8/1, V/V) to give compound **12a** or **12b** or **12c**.

6.1.2. Synthesis of target compounds

6.1.2.1. Synthesis of (E)-1-(2-hydrazineylidenepropyl)-2-methyl-4-nitro-1H-imidazole (3)

A mixture of acetonyl imidazole **2** (500 mg, 2.73 mmol) and hydrazinium hydroxide solution (683 mg, 13.65 mmol) was stirred in ethanol (15 mL) at room temperature for 8 h. The precipitation was filtered, washed with ethanol (3 mL × 3) and, dried to afford the desired molecule **3** as white solid (414 mg). Yield: 77%; Mp: 121–123 °C; ^{1}H NMR (400 MHz, 25 °C, DMSO-*d6*) δ 8.24 (s, 1H), 6.05 (s, 2H), 4.68 (s, 2H), 2.27 (s, 3H), 1.67 (s, 3H) ppm; ^{13}C NMR (151 MHz, 25 °C, DMSO-*d6*) δ 205.9, 156.8, 146.9, 145.6, 141.9, 133.4, 132.6, 130.4, 129.8, 129.5, 129.3, 123.8, 31.1, 12.8 ppm; HRMS calcd. for $C_7H_{11}N_5O_2$ $[M + H]^+$, 198.0991; found, 198.0989.

6.1.2.2. Synthesis of 2-methyl-4-nitro-1-((E)-2-(((E)-(5-nitrofuran-2-yl) methylene)hydrazineylidene)propyl)-1H-imidazole (4)

A mixture of hydrazineyl imidazole (70 mg, 0.35 mmol) and nitrofurfural (60 mg, 0.43 mmol) in ethanol (10 mL) was refluxed for 1 h with catalytic acetic acid. The solvent was distilled under reduced pressure to give crude product, which was purified by column chromatography on silica gel (Eluent: Dichloromethane/methanol = 30/1, V/V) to afford the desired compound **4** as white solid (70.9 mg). Yield: 63%; Mp: 186–188 °C; 1H NMR (600 MHz, DMSO-d6) δ 8.31 (s, 1H, imidazole-5-H), 8.14 (s, 1H, N=CH-furan), 7.78 (d, J = 3.9 Hz, 1H, furan-3-H), 7.30 (d, J = 3.9 Hz, 1H, furan-4-H), 5.07 (s, 2H, imidazole-1-CH2), 2.33 (s, 3H, imidazole-2-CH3), 2.05 (s, 3H, CH3) ppm. 13C NMR (151 MHz, DMSO-d6) δ 164.29, 152.85, 151.08, 151.06, 146.60, 146.43, 145.66, 123.60,

123.43, 118.63, 114.54, 51.59, 16.27, 13.09 ppm; HRMS calcd. for $C_{12}H_{12}N_6O_5$ $[M + H]^+$, 321.0941; found, 321.0942.

6.1.2.3. Synthesis of 2-methyl-4-nitro-1-((E)-2-(((E)-3-nitrobenzylidene) hydrazineylidene)propyl)-1H-imidazole ***(5a)***

A mixture of hydrazineyl imidazole (70 mg, 0.35 mmol) and 3-nitrobenzaldehyde (60 mg, 0.43 mmol) in ethanol (10 mL) was refluxed for 1 h with catalytic acetic acid. The solvent was distilled under reduced pressure to give crude product, which was purified by column chromatography on silica gel (Eluent: Dichloromethane/methanol = 30/1, V/V) to afford the desired molecule **5a** as white solid (62.4 mg). Yield: 55%; Mp: 205–207 °C; ^{1}H NMR (600 MHz, DMSO-d_6) δ 8.61 (t, J = 2.0 Hz, 1H, CH-Ph-2-*H*), 8.34 (s, 1H, N=C*H*-Ph), 8.33 (s, 1H, imidazole-5-*H*), 8.32 – 8.29 (m, 1H, CH-Ph-4-*H*), 8.24 (d, J = 7.8 Hz, 1H, CH-Ph-6-*H*), 7.77 (t, J = 8.0 Hz, 1H, CH-Ph-5-*H*), 5.07 (s, 2H, imidazole-1-CH_2), 2.35 (s, 3H, imidazole-2-CH_3), 2.08 (s, 3H, CH_3) ppm; ^{13}C NMR (151 MHz, DMSO-d_6) δ 163.53, 156.22, 148.64, 146.42, 145.66, 136.08, 134.51, 130.94, 125.76, 123.64, 122.75, 51.62, 16.15, 13.13 ppm; HRMS calcd. for $C_{14}H_{14}N_6O_4$ $[M + Na]^+$, 353.0974; found, 353.2661.

6.1.2.4. Synthesis of 1-((E)-2-(((E)-4-chlorobenzylidene)hydrazineylidene) propyl)-2-methyl-4-nitro-1H-imidazole ***(5b)***

A mixture of hydrazineyl imidazole (70 mg, 0.35 mmol) and 4-chlorobenzaldehyde (60 mg, 0.43 mmol) in ethanol (10 mL) was refluxed for 1 h with catalytic acetic acid. The solvent was distilled under reduced pressure to give crude product, which was purified by column chromatography on silica gel (Eluent: Dichloromethane/methanol = 30/1, V/V) to afford the target molecule **5b** as white solid (64 mg). Yield: 56%; Mp: 192–194 °C; ^{1}H NMR (400 MHz, 25 °C, DMSO-d_6) δ 8.31 (s, 1H), 8.20 (s, 1H), 7.83 (d, J = 8.5 Hz, 2H), 7.53 (d, J = 8.5 Hz, 2H), 5.04 (s, 2H), 2.34 (s, 3H), 2.07 (s, 3H) ppm; ^{13}C NMR (101 MHz, 25 °C, DMSO-d_6) δ 163.24, 157.23, 146.37, 145.69, 136.10, 133.29, 130.20, 129.39, 123.57, 51.67, 16.02, 13.12 ppm; HRMS calcd. for $C_{14}H_{14}ClN_5O_2$ $[M + H]^+$, 320.0914; found, 320.0918.

6.1.2.5. Synthesis of 2-methyl-4-nitro-1-((E)-2-(((E)-4-nitrobenzylidene) hydrazineylidene)propyl)-1H-imidazole ***(5c)***

A mixture of hydrazineyl imidazole (70 mg, 0.35 mmol) and 4-nitrobenzaldehyde (60 mg, 0.43 mmol) in ethanol (10 mL) was refluxed for 1 h with catalytic acetic acid. The solvent was distilled under reduced pressure to give crude product, which was purified by column chromatography on silica gel (Eluent: Dichloromethane/methanol = 30/1, V/V) to afford the desired compound **5c** as white solid (66 mg). Yield: 58%; Mp: 197–200 °C; ^{1}H NMR (600 MHz, DMSO-d_6) δ 8.32 (s, 1H, imidazole-5-*H*), 8.31 (s, 1H, N=C*H*-Ph), 8.30 (d, J = 4.9 Hz, 2H, CH-Ph-3-*H*, CH-Ph-5-*H*), 8.09 – 8.03 (m, 2H, CH-Ph-2-*H*, CH-Ph-6-*H*), 5.07 (s, 2H, imidazole-1-CH_2), 2.35 (s, 3H, imidazole-2-CH_3), 2.07 (s, 3H, CH_3) ppm; ^{13}C NMR (151 MHz, DMSO-d_6) δ 163.59, 156.12, 149.07, 146.42, 145.66, 140.32, 129.55, 124.45, 123.63, 51.59, 16.19, 13.13 ppm; HRMS calcd. for $C_{14}H_{14}N_6O_4$ $[M + Na]^+$, 353.0974; found, 353.0966.

6.1.2.6. Synthesis of 3-((E)-(((E)-1-(2-methyl-4-nitro-1H-imidazol-1-yl) propan-2-ylidene)hydrazineylidene)methyl)pyridine ***(6a)***

Compound **6a** was produced according the preparative method of derivative **4**, with hydrazineyl imidazole **3** and 3-pyridinecarboxaldehyde as substrates. White solid; Yield: 72%; Mp: >250 °C; ^{1}H NMR (400 MHz, DMSO-d_6) δ 8.94 (d, *J* = 2.1 Hz, 1H), 8.65 (dd, *J* = 4.8, 1.7 Hz, 1H), 8.31 (s, 1H), 8.25 (s, 1H), 8.19 (dt, *J* = 7.9, 2.0 Hz, 1H), 7.49 (dd, *J* = 8.0, 4.8 Hz, 1H), 5.05 (s, 2H), 2.34 (s, 3H), 2.07 (s, 3H) ppm; ^{13}C NMR (101 MHz, DMSO-d_6) δ 163.36, 155.89, 152.04, 150.06, 146.38, 145.70, 135.07, 130.17, 124.41, 123.57, 51.63, 16.06, 13.12 ppm; HRMS calcd. for $C_{18}H_{19}N_5O_3$ $[M + H]^+$, 354.1566; found, 354.1568.

*6.1.2.7. Synthesis of 3-((E)-(((E)-1-(2-methyl-4-nitro-1H-imidazol-1-yl) propan-2-ylidene)hydrazineylidene)methyl)pyridin-2-ol (**6b**)*

Compound **6b** was produced according the preparative method of derivative **4**, with hydrazineyl imidazole **3** and 2-hydroxynicotinaldehyde as substrates. Yellow solid; Yield: 93%; Mp: >250 °C; ^{1}H NMR (400 MHz, DMSO-d_6) δ 12.06 (s, 1H), 8.29 (s, 1H), 8.17 (s, 1H), 8.09 (d, *J* = 7.2, 1H), 7.58 (d, *J* = 7.2, 1H), 6.31 (t, *J* = 6.8 Hz, 1H), 5.00 (s, 2H), 2.32 (s, 3H), 2.02 (s, 3H) ppm; ^{13}C NMR (101 MHz, DMSO-d_6) δ 162.49, 161.89, 153.68, 146.35, 145.68, 139.36, 138.72, 123.67, 123.52, 105.93, 51.73, 15.88, 13.10 ppm; HRMS calcd. for $C_{18}H_{19}N_5O_3$ $[M + H]^+$, 354.1566; found, 354.1567.

*6.1.2.8. Synthesis of 5-((E)-(((E)-1-(2-methyl-4-nitro-1H-imidazol-1-yl) propan-2-ylidene) hydrazineylidene)methyl)pyridin-2-ol (**6c**)*

Compound **6c** was produced according the preparative method of derivative **4**, with hydrazineyl imidazole **3** and 6-hydroxynicotinaldehyde as substrates. White solid; Yield: 86%; Mp: >250 °C; ^{1}H NMR (400 MHz, DMSO-d_6) δ 11.96 (s, 1H), 8.28 (s, 1H), 8.06 (s, 1H), 7.92 (dd, *J* = 9.7, 2.5 Hz, 1H), 7.82 (d, *J* = 2.5 Hz, 1H), 6.41 (d, *J* = 9.6 Hz, 1H), 4.99 (s, 2H), 2.30 (s, 3H), 2.06 (s, 3H) ppm; ^{13}C NMR (101 MHz, DMSO-d_6) δ 162.94, 162.86, 156.12, 146.35, 145.63, 140.35, 137.49, 123.58, 121.14, 113.61, 51.69, 15.84, 13.10 ppm; HRMS calcd. for $C_{18}H_{19}N_5O_3$ $[M + H]^+$, 354.1566; found, 354.1566.

6.1.2.9. Synthesis of 5-((E)-(((E)-1-(2-methyl-4-nitro-1H-imidazol-1-yl) propan-2-ylidene) hydrazineylidene)methyl)-2-(methylthio)pyrimidin-4-amine (7)

Compound **7** was produced according the preparative method of derivative **4**, with hydrazineyl imidazole **3** and 4-amino-2-(methylthio)pyrimidine-5-carbaldehyde as substrates. White solid; Yield: 60%; Mp: >250 °C; ^{1}H NMR (400 MHz, 25 °C, DMSO-d_6) δ 8.35 (s, 1H), 8.31 (s, 1H), 8.30 (s, 1H), 8.13 (d, *J* = 23.0 Hz, 2H), 5.05 (s, 2H), 2.47 (s, 3H), 2.32 (s, 3H), 2.07 (s, 3H) ppm; ^{13}C NMR (101 MHz, 25 °C, DMSO-d_6) δ 172.68, 163.95, 160.49, 160.19, 159.46, 146.40, 145.67, 123.57, 106.18, 51.67, 16.31, 13.83, 13.12 ppm; HRMS calcd. for $C_{13}H_{16}N_8O_2S$ $[M + H]^+$, 349.1195; found, 349.1188.

*6.1.2.10. Synthesis of 5-chloro-1,3-dimethyl-4-((E)-(((E)-1-(2-methyl-4-nitro-1H-imidazol-1-yl) propan-2-ylidene)hydrazineylidene)methyl)-1H-pyrazole (**8**)*

Compound **8** was produced according the preparative method of derivative **4**, with hydrazineyl imidazole **3** and 5-chloro-1,3-dimethyl-1H-pyrazole-4-carbaldehyde as substrates. White solid; Yield: 39%; Mp: 171–173 °C; ^{1}H NMR (400 MHz, 25 °C, DMSO-d_6) δ 8.29 (s, 1H), 8.01 (s, 1H), 5.01 (s, 2H), 3.76 (s, 3H), 2.34 (s, 3H), 2.32 (s, 3H), 2.06 (s, 3H) ppm; ^{13}C NMR (101 MHz, 25 °C, DMSO-d_6) δ 163.33, 150.96, 148.04, 146.36, 145.68, 129.33,

123.54, 111.37, 51.67, 36.42, 16.02, 14.54, 13.13 ppm; HRMS calcd. for $C_{13}H_{16}ClN_7O_2$ [M + H]$^+$, 338.1132; found, 338.1126.

*6.1.2.11. Synthesis of (E)-2-((Z)-3-(2-methyl-4-nitro-1H-imidazol-1-yl)-4-phenylbut-3-en-2-ylidene) hydrazine-1-carboxamide (**10a**)*

A mixture of phenylenone imidazole **9a** (100 mg, 0.32 mmol) and acetic acid (19 mg, 0.32 mmol) was stirred in ethanol (15 mL) at 45 °C for 1 h. Then, semicarbazide (45 mg, 0.64 mmol) was added and the above reaction system was heated and kept at 80 °C for 3 h. The solvent was distilled under reduced pressure to give crude product, which was purified by column chromatography on silica gel (Eluent: Dichloromethane/methanol = 10/1, V/V) to afford the desired compound **10a** as yellow solid (49 mg). Yield: 41%; Mp: 181–182 °C; ^{1}H NMR (600 MHz, 25 °C, DMSO-*d6*) δ 9.78 (s, 1H), 8.43 (s, 1H), 7.46 (s, 1H), 7.33 (t, *J* = 7.6 Hz, 3H), 6.95 (d, *J* = 6.6 Hz, 2H), 2.22 (s, 3H), 2.01 (s, 3H) ppm; ^{13}C NMR (151 MHz, 25 °C, DMSO-*d6*) δ 205.9, 156.8, 146.9, 145.6, 141.9, 133.4, 132.6, 130.4, 129.8, 129.5, 129.3, 123.8, 31.1, 12.8 ppm; HRMS calcd. for $C_{15}H_{16}N_6O_3$ [M + Na]$^+$, 351.1182; found, 351.1192.

*6.1.2.12. Synthesis of (E)-2-((Z)-4-(4-chlorophenyl)-3-(2-methyl-4-nitro-1H-imidazol-1-yl) but-3-en-2-ylidene)hydrazine-1-carboxamide (**10b**)*

The preparative method of molecule **10a** was slightly altered to produce compound **10b** with phenylenone imidazole **9b** and semicarbazide as substrates. Yellow solid; Yield: 49%; Mp: 169–170 °C; ^{1}H NMR (600 MHz, 25 °C, DMSO-*d6*) δ 9.79 (s, 1H), 8.42 (s, 1H), 7.48 (s, 1H), 7.42 (d, *J* = 8.6 Hz, 2H), 6.96 (d, *J* = 8.6 Hz, 2H), 2.21 (s, 3H), 2.01 (s, 3H) ppm; ^{13}C NMR (151 MHz, 25 °C, DMSO-*d6*) δ 206.8, 156.7, 147.0, 145.5, 141.8, 134.3, 133.2, 132.4, 130.9, 129.6, 129.1, 123.7, 31.1, 12.8 ppm; HRMS calcd. for $C_{15}H_{15}ClN_6O_3$ [M + H]$^+$, 363.0972; found, 363.0966.

*6.1.2.13. Synthesis of (2E,3Z)-3-(2-methyl-4-nitro-1H-imidazol-1-yl)-4-phenylbut-3-en-2-one oxime (**11a**)*

The preparative method of **10a** was slightly altered to produce compound **11a** with phenylenone imidazole **9a** and hydroxylamine hydrochloride as substrates. Yellow solid; Yield: 45%; Mp: 198–199 °C; ^{1}H NMR (600 MHz, 25 °C, DMSO-*d6*) δ 11.76 (s, 1H), 8.45 (s, 1H), 7.43 (s, 1H), 7.33 (t, *J* = 6.6 Hz, 3H), 6.93 (d, *J* = 6.4 Hz, 2H), 2.21 (s, 3H), 2.02 (s, 3H) ppm; ^{13}C NMR (151 MHz, 25 °C, DMSO-*d6*) δ 152.6, 147.3, 145.6, 133.7, 131.7, 131.3, 130.2, 129.9, 129.7, 124.3, 31.6, 13.2 ppm; HRMS calcd. for $C_{14}H_{14}N_4O_3$ [M + H]$^+$, 287.1144; found, 287.1145.

*6.1.2.14. Synthesis of (2E,3Z)-3-(2-methyl-4-nitro-1H-imidazol-1-yl)-4-phenylbut-3-en-2-one O-methyl oxime (**11b**)*

The preparative method of **10a** was slightly altered to produce compound **11b** with phenylenone imidazole **9a** and methoxyammonium chloride as substrates. Yellow solid; Yield: 44%; Mp: >250 °C; ^{1}H NMR (600 MHz, 25 °C, DMSO-*d6*) δ 8.41 (s, 1H), 7.50 (s, 1H), 7.36–7.33 (m, 3H), 6.91 (d, *J* = 5.5 Hz, 2H), 3.76 (s, 3H), 2.22 (s, 3H), 2.04 (s, 3H) ppm; ^{13}C NMR (151 MHz, 25 °C, DMSO-*d6*) δ 153.0, 146.9, 145.3, 133.1, 132.4, 130.1, 129.5, 129.4, 123.6, 62.8, 31.1, 12.9 ppm; HRMS calcd. for $C_{17}H_{16}N_4O_3$ [M + Na]$^+$, 323.1120; found, 323.1119.

*6.1.2.15. Synthesis of (2E,3Z)-4-(4-chlorophenyl)-3-(2-methyl-4-nitro-1H-imidazol-1-yl) but-3-en-2-one oxime **(11c)***

The preparative method of **10a** was slightly altered to produce compound **11c** with phenylenone imidazole **9b** and hydroxylamine hydrochloride as substrates. Yellow solid; Yield: 48%; Mp: >250 °C; ^{1}H NMR (600 MHz, 25 °C, DMSO-*d6*) δ 11.78 (s, 1H), 8.35 (s, 1H), 7.76 (s, 1H), 7.39 (d, *J* = 8.4 Hz, 2H), 7.23 (d, *J* = 8.4 Hz, 2H), 2.47 (s, 3H), 2.19 (s, 3H) ppm; ^{13}C NMR (151 MHz, 25 °C, $CDCl_3$) δ 167.6, 156.9, 146.7, 137.6, 129.9, 127.9, 124.5, 120.5, 112.5, 30.8, 13.1 ppm; HRMS calcd. for $C_{14}H_{13}ClN_4O_3$ $[M+H]^+$, 321.0754; found, 321.0752.

*6.1.2.16. Synthesis of (2E,3Z)-4-(4-chlorophenyl)-3-(2-methyl-4-nitro-1H-imidazol-1-yl) but-3-en-2-one O-methyl oxime **(11d)***

The preparative method of **10a** was slightly altered to produce compound **11d** with phenylenone imidazole **9b** and methoxyammonium chloride as substrates. Yellow solid; Yield: 47%; Mp: >250 °C; ^{1}H NMR (600 MHz, 25 °C, DMSO-*d6*) δ 8.37 (s, 1H), 8.29 (s, 1H), 7.81 (d, *J* = 8.1 Hz, 1H), 7.16 (s, 1H), 7.08 (d, *J* = 8.2 Hz, 1H), 6.42 (s, 1H), 2.57 (s, 3H), 2.41 (s, 3H), 2.04 (s, 3H) ppm; ^{13}C NMR (151 MHz, 25 °C, DMSO-*d6*) δ 150.9, 147.4, 145.2, 136.2, 130.9, 130.4, 130.1, 129.7, 120.6, 62.9, 12.9, 10.9 ppm; HRMS calcd. for $C_{15}H_{15}ClN_4O_3$ $[M+H]^+$, 335.0911; found, 335.0911.

*6.1.2.17. Synthesis of (E)-2-((Z)-4-(1H-indol-3-yl)-3-(2-methyl-4-nitro-1H-imidazol-1-yl) but-3-en-2-ylidene)hydrazine-1-carboxamide **(13a)***

The preparative method of **10a** was slightly altered to produce compound **13a** with indolylenone imidazole **12a** and semicarbazide as substrates. Yellow solid; Yield: 54%; Mp: >250 °C; ^{1}H NMR (600 MHz, 25 °C, DMSO-*d6*) δ 10.96 (s, 1H), 7.42 (t, *J* = 8.7 Hz, 2H), 7.31 (s, 1H), 7.22 (d, *J* = 8.0 Hz, 1H), 7.16 (s, 1H), 6.15 (d, *J* = 10.3 Hz, 1H), 5.90 (s, 1H), 2.21 (s, 3H), 2.01 (s, 3H) ppm; ^{13}C NMR (151 MHz, 25 °C, DMSO-*d6*) δ 206.9, 157.0, 147.1, 145.7, 142.7, 136.4, 128.5, 127.8, 126.3, 124.2, 122.3, 121.3, 119.6, 111.2, 110.3, 31.2, 12.4 ppm; HRMS calcd. for $C_{17}H_{17}N_7O_3$ $[M + H]^+$, 368.1471; found, 368.1475.

*6.1.2.18. Synthesis of (E)-2-((Z)-4-(6-chloro-1H-indol-3-yl)-3-(2-methyl-4-nitro-1H-imidazol-1-yl) but-3-en-2-ylidene)hydrazine-1-carboxamide **(13b)***

The preparative method of **10a** was slightly altered to produce compound **13b** with indolylenone imidazole **12b** and semicarbazide as substrates. Yellow solid; Yield: 52%; Mp: 217–218 °C; ^{1}H NMR (600 MHz, 25 °C, DMSO-*d6*) δ 11.54 (s, 1H), 9.62 (s, 1H), 8.39 (s, 1H), 8.03 (d, *J* = 8.5 Hz, 1H), 7.64 (s, 1H), 7.49 (s, 1H), 7.18 (d, *J* = 8.6 Hz, 1H), 6.06 (s, 1H), 3.17 (s, 1H), 2.29 (s, 3H), 2.09 (s, 3H), 2.06 (s, 3H) ppm; ^{13}C NMR (151 MHz, 25 °C, DMSO-*d6*) δ 206.8, 157.0, 147.1, 145.4, 142.7, 136.4, 128.5, 127.7, 126.3, 123.2, 122.3, 121.0, 120.6, 112.2, 109.3, 31.1, 12.6 ppm; HRMS calcd. for $C_{17}H_{16}ClN_7O_3$ $[M + Na]^+$, 424.0901; found, 424.0903.

*6.1.2.19. Synthesis of (E)-2-((Z)-4-(6-methyl-1H-indol-3-yl)-3-(2-methyl-4-nitro-1H-imidazol-1-yl) but-3-en-2-ylidene)hydrazine-1-carboxamide **(13c)***

The preparative method of **10a** was slightly altered to produce compound **13c** with indolylenone imidazole **12c** and semicarbazide as substrates. Yellow solid; Yield: 51%; Mp: >250 °C; ^{1}H NMR (600 MHz, 25 °C, DMSO-*d6*) δ 11.49 (s, 1H), 8.38 (s, 1H), 7.82 (d, *J* = 8.2 Hz, 1H), 7.61 (s, 1H), 7.21 (s, 1H), 6.89 (s, 1H), 5.93 (s, 1H),

2.20 (s, 3H), 2.05 (s, 3H), 2.00 (s, 3H) ppm; ^{13}C NMR (151 MHz, 25 °C, DMSO-*d*$_6$) δ 206.8, 158.2, 136.4, 132.2, 127.6, 124.7, 123.4, 122.9, 122.5, 112.2, 111.7, 31.7, 21.7, 12.6 ppm; HRMS calcd. for $C_{18}H_{19}N_7O_3$ $[M + H]^+$, 382.1628; found, 382.1633.

*6.1.2.20. Synthesis of (2E,3Z)-4-(1H-indol-3-yl)-3-(2-methyl-4-nitro-1H-imidazol-1-yl) but-3-en-2-one oxime (**14a**)*

A mixture of indolylenone imidazole **12a** (100 mg, 0.32 mmol) and acetic acid (19 mg, 0.32 mmol) was stirred in ethanol (15 mL) at 45 °C for 1 h. Hydroxylamine hydrochloride (45 mg, 0.64 mmol) was added and the above reaction system was heated and kept at 80 °C for 3 h. The solvent was distilled under reduced pressure to give crude product, which was purified by column chromatography on silica gel (Eluent: Dichloromethane/methanol = 10/1, V/V) to afford the desired compound **14a** as yellow solid (53 mg). Yield: 51%; Mp: >250 °C; ^{1}H NMR (600 MHz, 25 °C, DMSO-*d*$_6$) δ 11.62 (s, 1H), 11.44 (s, 1H), 8.41 (s, 1H), 7.96 (d, *J* = 7.8 Hz, 1H), 7.60 (s, 1H), 7.43 (d, *J* = 8.0 Hz, 1H), 7.19 (t, *J* = 7.0 Hz, 1H), 7.15 (t, *J* = 7.0 Hz, 1H), 5.94 (s, 1H), 2.28 (s, 3H), 2.08 (s, 3H) ppm; ^{13}C NMR (151 MHz, 25 °C, DMSO-*d*$_6$) δ 152.3, 147.0, 145.1, 135.9, 127.4, 126.3, 125.1, 123.3, 122.9, 120.7, 118.9, 112.5, 108.8, 12.6, 10.7 ppm; HRMS calcd. for $C_{16}H_{15}N_5O_3$ $[M + H]^+$, 326.1253; found, 326.1253.

*6.1.2.21. Synthesis of (2E,3Z)-4-(1H-indol-3-yl)-3-(2-methyl-4-nitro-1H-imidazol-1-yl) but-3-en-2-one O-methyl oxime (**14b**)*

The preparative method of **14a** was slightly altered to produce compound **14b** with indolylenone imidazole **12a** and methoxyammonium chloride as substrates. Yellow solid; Yield: 55%; Mp: >250 °C; ^{1}H NMR (600 MHz, 25 °C, DMSO-*d*$_6$) δ 11.54 (s, 1H), 8.38 (s, 1H), 7.97 (s, 1H), 7.67 (s, 1H), 7.43 (d, *J* = 7.9 Hz, 1H), 7.20 (t, *J* = 7.1 Hz, 1H), 7.16 (t, *J* = 7.4 Hz, 1H), 5.92 (s, 1H), 3.73 (s, 3H), 2.29 (s, 3H), 2.09 (s, 3H) ppm; ^{13}C NMR (151 MHz, 25 °C, DMSO-*d*$_6$) δ 153.1, 147.1, 145.1, 135.9, 127.5, 125.6, 125.0, 124.5, 123.1, 120.8, 118.9, 112.5, 108.8, 62.5, 31.1, 12.6 ppm; HRMS calcd. for $C_{17}H_{17}N_5O_3$ $[M + H]^+$, 340.1410; found, 340.1410.

*6.1.2.22. Synthesis of (2E,3Z)-4-(6-chloro-1H-indol-3-yl)-3-(2-methyl-4-nitro-1H-imidazol-1-yl) but-3-en-2-one oxime (**14c**)*

The preparative method of **14a** was slightly altered to produce compound **14c** with indolylenone imidazole **12b** and hydroxylamine hydrochloride as substrates. Yellow solid; Yield: 56%; Mp: >250 °C; ^{1}H NMR (600 MHz, 25 °C, DMSO-*d*$_6$) δ 12.03 (s, 1H), 11.49 (s, 1H), 8.42 (s, 1H), 8.03 (d, *J* = 8.6 Hz, 1H), 7.59 (s, 1H), 7.50 (s, 1H), 7.17 (d, *J* = 8.6 Hz, 1H), 5.99 (s, 1H), 2.28 (s, 3H), 2.07 (s, 3H) ppm; ^{13}C NMR (151 MHz, 25 °C, DMSO-*d*$_6$) δ 152.3, 147.1, 145.0, 136.3, 127.7, 127.1, 126.2, 126.1, 123.2, 122.5, 121.0, 120.5, 112.2, 109.1, 49.1, 31.1 ppm; HRMS calcd. for $C_{16}H_{14}ClN_5O_3$ $[M + H]^+$, 360.0863; found, 360.0864.

*6.1.2.23. Synthesis of (2E,3Z)-4-(6-chloro-1H-indol-3-yl)-3-(2-methyl-4-nitro-1H-imidazol-1-yl) but-3-en-2-one O-methyl oxime (**14d**)*

The preparative method of **14a** was slightly altered to produce compound **14d** with indolylenone imidazole **12b** and methoxyammonium chloride as substrates. Yellow solid; Yield: 47%; Mp: 127–128 °C; ^{1}H NMR (600 MHz, 25 °C, DMSO-*d*$_6$) δ 11.58 (s, 1H), 8.38 (s, 1H), 8.03 (d, *J* = 8.6 Hz, 1H), 7.66 (s, 1H), 7.50 (d, *J* = 1.6 Hz, 1H), 7.18 (d, *J* = 8.6 Hz, 1H), 5.97 (s, 1H), 3.74 (s, 3H), 2.28 (s, 3H), 2.09 (s, 3H) ppm; ^{13}C NMR (151 MHz,

25 °C, DMSO-d_6) δ 153.1, 147.1, 145.1, 136.3, 127.8, 126.5, 126.3, 125.8, 124.1, 123.1, 121.1, 120.6, 112.2, 109.0, 62.5, 31.1, 12.6 ppm; HRMS calcd. for $C_{17}H_{16}ClN_5O_3$ $[M + Na]^+$, 396.0839; found, 396.0838.

*6.1.2.24. Synthesis of (2E,3Z)-4-(6-methyl-1H-indol-3-yl)-3-(2-methyl-4-nitro-1H-imidazol-1-yl) but-3-en-2-one oxime (**14e**)*

The preparative method of **14a** was slightly altered to produce compound **14e** with indolylenone imidazole **12c** and hydroxylamine hydrochloride as substrates. Yellow solid; Yield: 56%; Mp: >250 °C; ^{1}H NMR (600 MHz, 25 °C, DMSO-d_6) δ 11.85 (s, 1H), 8.41 (s, 1H), 8.34 (s, 1H), 7.80 (d, J = 7.9 Hz, 1H), 7.28 (s, 1H), 7.06 (d, J = 7.9 Hz, 1H), 6.28 (s, 1H), 2.58 (s, 3H), 2.42 (s, 3H), 2.06 (s, 3H) ppm; ^{13}C NMR (151 MHz, 25 °C, DMSO-d_6) δ 206.8, 192.7, 147.2, 145.3, 136.9, 134.6, 133.1, 129.3, 126.7, 125.5, 123.6, 123.0, 118.8, 112.7, 108.3, 79.6, 60.2, 56.5, 49.1, 31.1, 21.7, 12.5 ppm; HRMS calcd. for $C_{17}H_{17}N_5O_3$ $[M + H]^+$, 340.1410; found, 340.1411.

*6.1.2.25. Synthesis of (2E,3Z)-4-(6-methyl-1H-indol-3-yl)-3-(2-methyl-4-nitro-1H-imidazol-1-yl) but-3-en-2-one O-methyl oxime (**14f**)*

The preparative method of **14a** was slightly altered to produce compound **14f** with indolylenone imidazole **12c** and methoxyammonium chloride as substrates. Yellow solid; Yield: 53%; Mp: >250 °C; ^{1}H NMR (600 MHz, 25 °C, DMSO-d_6) δ 11.41 (s, 1H), 8.37 (s, 1H), 7.82 (d, J = 8.2 Hz, 1H), 7.63 (s, 1H), 7.21 (s, 1H), 6.99 (d, J = 8.1 Hz, 1H), 5.85 (s, 1H), 3.73 (s, 3H), 2.40 (s, 3H), 2.28 (s, 3H), 2.08 (s, 3H) ppm; ^{13}C NMR (151 MHz, 25 °C, DMSO-d_6) δ 153.1, 147.0, 145.2, 136.4, 132.4, 125.4, 125.0, 124.8, 124.7, 123.2, 122.6, 118.7, 112.2, 108.8, 62.4, 31.1, 21.7, 12.6 ppm; HRMS calcd. for $C_{18}H_{19}N_5O_3$ $[M + H]^+$, 354.1566; found, 354.1566.

*6.1.2.26. Synthesis of (Z)-4-(6-hydroxypyridin-3-yl)-3-(2-methyl-4-nitro-1H-imidazol-1-yl) but-3-en-2-one (**15**)*

A mixture of acetonyl imidazole **2** (50 mg, 0.27 mmol) and 6-hydroxynicotinaldehyde (23 mg, 0.27 mmol) in ethanol (10 mL) was refluxed for 8 h with catalytic piperidine. The solvent was distilled under reduced pressure to give crude product, which was purified by column chromatography on silica gel (Eluent: Dichloromethane/methanol = 30/1, V/V) to afford the desired compound **15** as white solid (40 mg). Yield: 50%; Mp: >250 °C; ^{1}H NMR (400 MHz, DMSO-d_6) δ 12.24 (s, 1H), 8.32 (s, 1H), 8.03 (s, 1H), 7.84 (d, J = 2.7 Hz, 1H), 6.35 (d, J = 2.6 Hz, 1H), 6.32 (s, 1H), 2.46 (s, 3H), 2.07 (s, 3H) ppm; ^{13}C NMR (101 MHz, DMSO-d_6) δ 193.30, 161.77, 147.16, 145.63, 144.07, 138.23, 138.01, 128.19, 123.18, 121.65, 110.71, 25.78, 12.70 ppm; HRMS calcd. for $C_{15}H_{16}N_6O_3$ $[M + Na]^+$, 351.1182; found, 351.1192.

*6.1.2.27. Synthesis of (E)-4-(dimethylamino)-1-(2-methyl-4-nitro-1H-imidazol-1-yl) but-3-en-2-one (**16**)*

A mixture of acetonyl imidazole **2** (200 mg, 1.09 mmol) and *N,N*-dimethylformamide dimethyl acetal (260 mg, 2.18 mmol) in ethanol (10 mL) was refluxed for 1 h with catalytic triethylamine. The precipitation was filtered, washed with ethanol (3 mL × 3) and, dried to afford the desired compound **16** as white solid (119 mg). Yield: 46%; Mp: 178–180 °C; ^{1}H NMR (400 MHz, 25 °C, DMSO-d_6) δ 8.16 (s, 1H), 7.77 (s, 1H), 3.13 (s, 3H), 2.44–1.71 (m, 9H) ppm; ^{13}C NMR (101 MHz, 25 °C, DMSO-d_6) δ 205.9, 156.8, 146.9, 145.6, 141.9, 133.4, 132.6, 130.4, 129.8, 129.5, 129.3, 123.8, 31.1, 12.8 ppm; HRMS calcd. for $C_{10}H_{14}N_4O_3$ $[M + H]^+$, 239.1144; found, 239.1142.

*6.1.2.28. Synthesis of 1-(4-hydroxyphenyl)-2-(2-methyl-4-nitro-1H-imidazol-1-yl)ethan-1-one (**17**)*

A mixture of 2-methyl-5-nitro-1*H*-imidazole **1** (500 mg, 3.93 mmol), 2-bromo-4'-hydroxyacetophenone (846 mg, 3.93 mmol), and potassium carbonate (816 mg, 5.90 mmol) in acetonitrile (20 mL) was stirred at 80 °C for 10 h. Most of the solvent was distilled under reduced pressure, and the residue was poured into water (40 mL). Acetic acid was used to adjust the pH value to about 6 under stirring. The precipitation was filtered, washed with ethanol (2 mL × 3) and, dried to afford the desired compound **17** as yellow solid (960 mg). Yield: 93%; Mp: 165–167 °C. ^{1}H NMR (400 MHz, 25 °C, DMSO-d_6) δ 10.60 (s, 1H), 8.22 (s, 1H), 7.95–7.91 (m, 2H), 6.97–6.91 (m, 2H), 5.77 (d, J = 17.6 Hz, 2H), 2.24 (s, 3H) ppm.

*6.1.2.29. Synthesis of 4-(1-hydroxy-2-(2-methyl-4-nitro-1H-imidazol-1-yl)ethyl)phenol (**18**)*

Sodium borohydride (29 mg, 0.77 mmol) was slowly added into the mixture of methanol (10 mL) and compound **17** (80 mg, 0.31 mmol) in the ice bath. The reaction system was stirred at room temperature for 10 h. Afterwards, hydrochloric acid (2 mol/L) was used to adjust the pH value to about 3 under stirring, and then sodium hydroxide solution (1 mol/L) was used to neutralize the residual hydrochloric acid. Methanol was distilled under reduced pressure, and the precipitation was filtered, washed with ethanol (1 mL × 3) and, dried to afford the desired compound **18** as brown solid (59 mg). Yield: 73%; Mp: 232–234 °C; ^{1}H NMR (400 MHz, 25 °C, DMSO-d_6) δ 9.36 (s, 1H), 8.18 (s, 1H), 7.13 (d, J = 8.1 Hz, 2H), 6.73 (d, J = 8.0 Hz, 2H), 5.63 (s, 1H), 4.82–4.78 (m, 1H), 4.07 (m, 2H), 2.19 (s, 3H) ppm; ^{13}C NMR (151 MHz, 25 °C, DMSO-d_6) δ 205.9, 156.8, 146.9, 145.6, 141.9, 133.4, 132.6, 130.4, 129.8, 129.5, 129.3, 123.8, 31.1, 12.8 ppm; HRMS calcd. for $C_{12}H_{13}N_3O_4$ $[M + H]^+$, 264.0984; found, 264.0976.

*6.1.2.30. Synthesis of (E)-1-(4-hydroxyphenyl)-2-(2-methyl-4-nitro-1H-imidazol-1-yl)ethan-1-one O-methyl oxime (**19**)*

A mixture of compound **17** (60 mg, 0.23 mmol) and methoxyammonium chloride (23 mg, 0.28 mmol) in ethanol (10 mL) was stirred at 60 °C for 5 h with catalytic pyridine. The solvent was distilled under reduced pressure to give crude product, which was purified by column chromatography on silica gel (Eluent: Dichloromethane/methanol = 50/1, V/V) to afford the desired compound **19** as white solid (46 mg). Yield: 69%; Mp: 215–217 °C; ^{1}H NMR (600 MHz, 25 °C, DMSO-d_6) δ 9.78 (s, 1H), 8.43 (s, 1H), 7.46 (s, 1H), 7.33 (t, J = 7.6 Hz, 3H), 6.95 (d, J = 6.6 Hz, 2H), 2.22 (s, 3H), 2.01 (s, 3H) ppm; ^{13}C NMR (151 MHz, 25 °C, DMSO-d_6) δ 205.9, 156.8, 146.9, 145.6, 141.9, 133.4, 132.6, 130.4, 129.8, 129.5, 129.3, 123.8, 31.1, 12.8 ppm; HRMS calcd. for $C_{13}H_{14}N_4O_4$ $[M + H]^+$, 291.1093; found, 291.1090.

7. Spectra of ^{1}H NMR, ^{13}C NMR, HRMS, and HPLC

*7.1. Spectra of compound **3***

^{1}H NMR spectrum (400 MHz, 25 °C, DMSO-d_6)

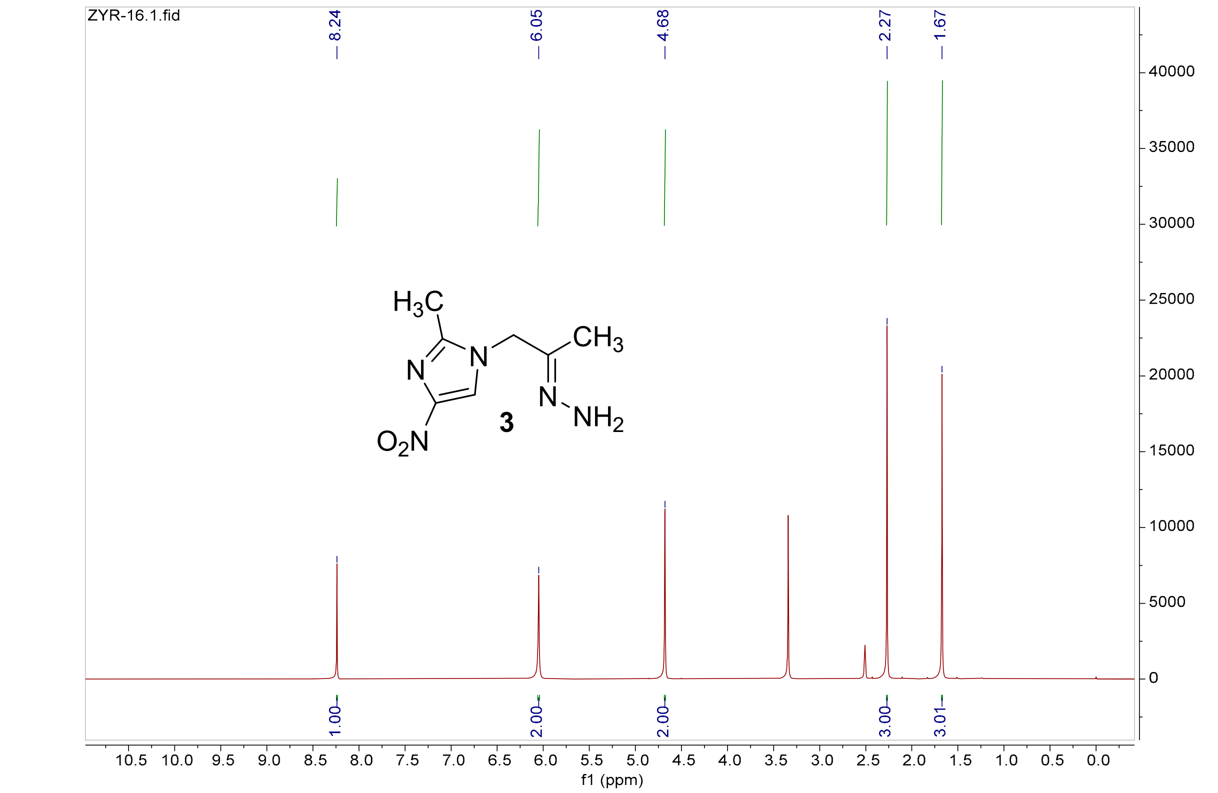

^{13}C NMR spectrum (101 MHz, 25 °C, DMSO-d_6)

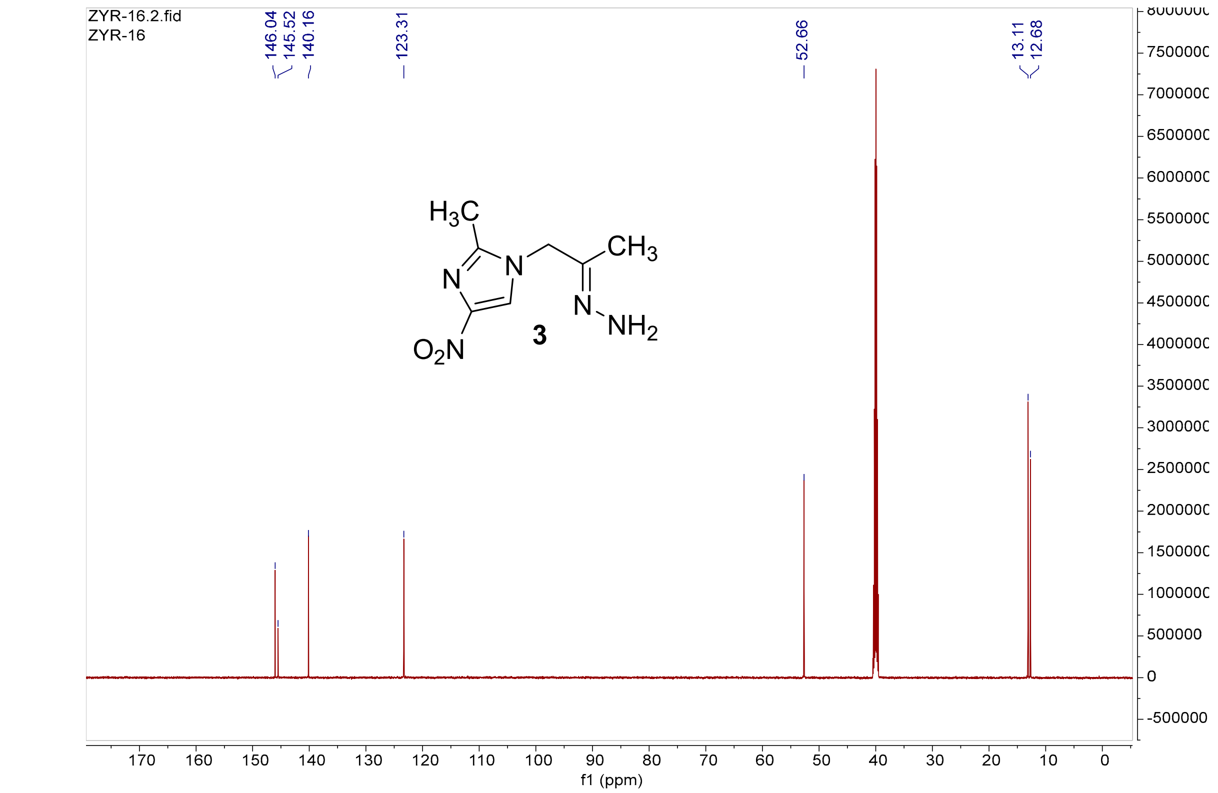

HRMS spectrum

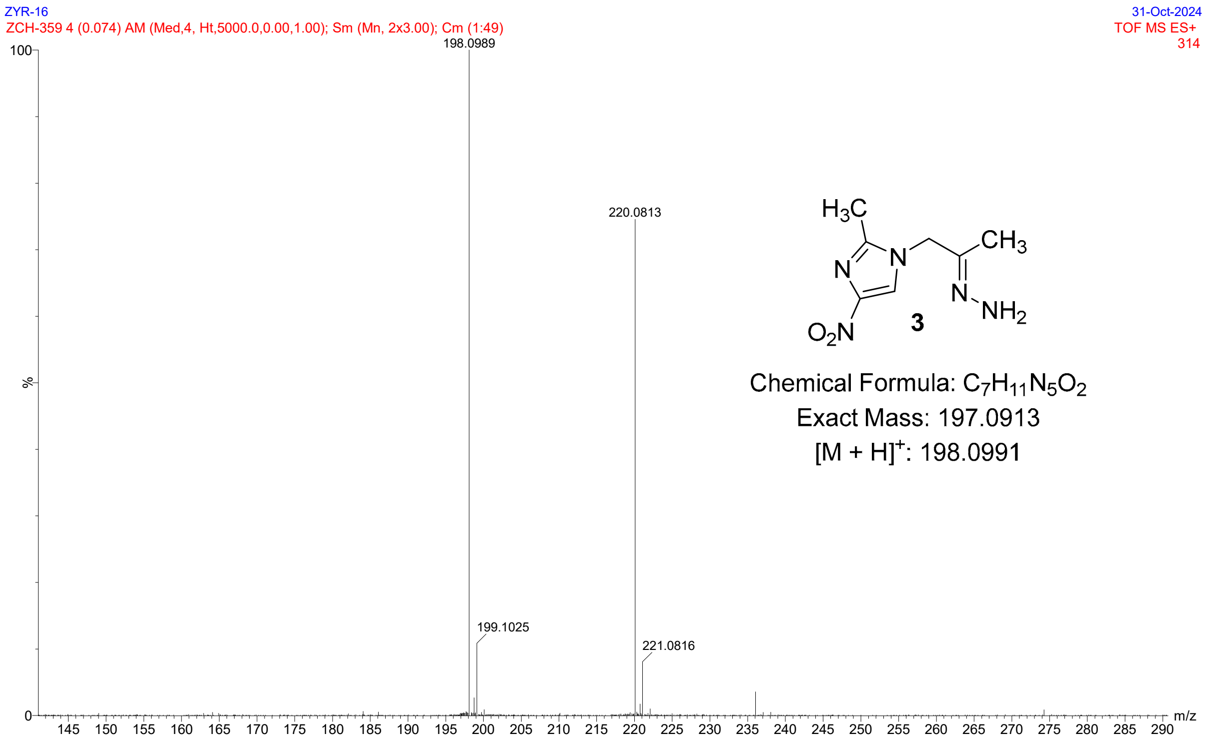

*7.2. Spectra of compound **4***

^{1}H NMR spectrum (400 MHz, 25 °C, DMSO-d_6)

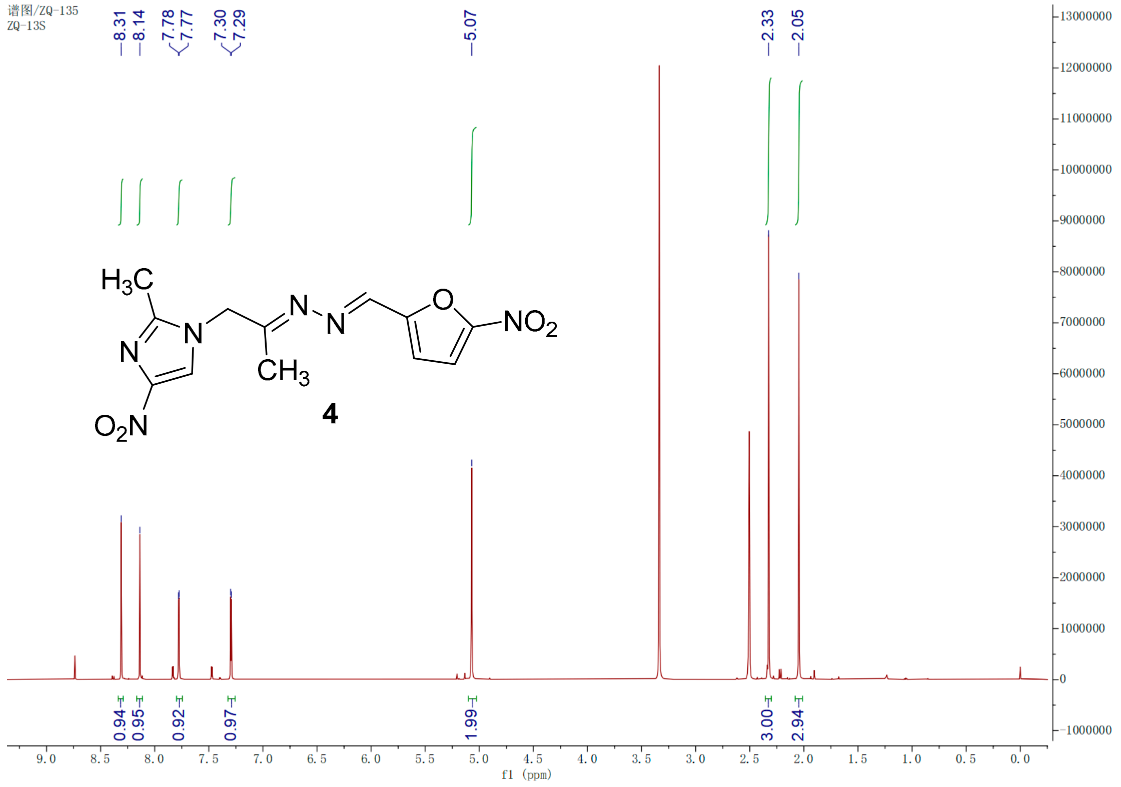

^{13}C NMR spectrum (101 MHz, 25 °C, DMSO-d_6)

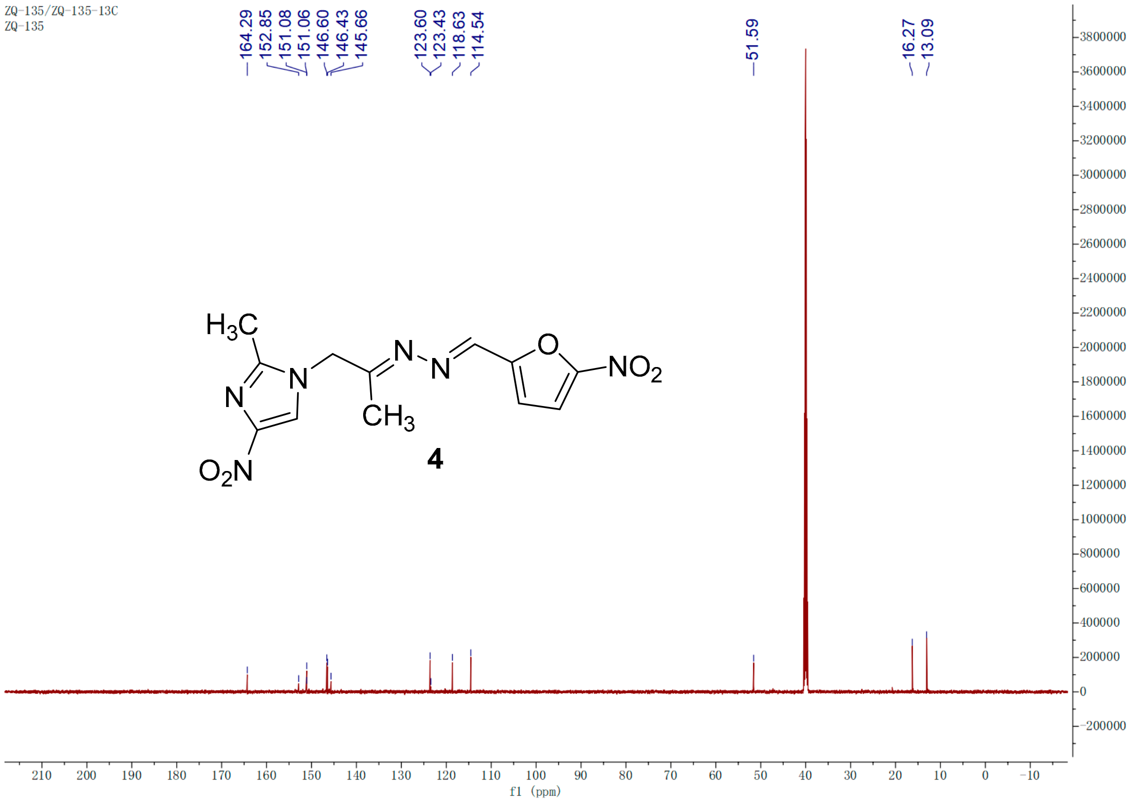

HRMS spectrum

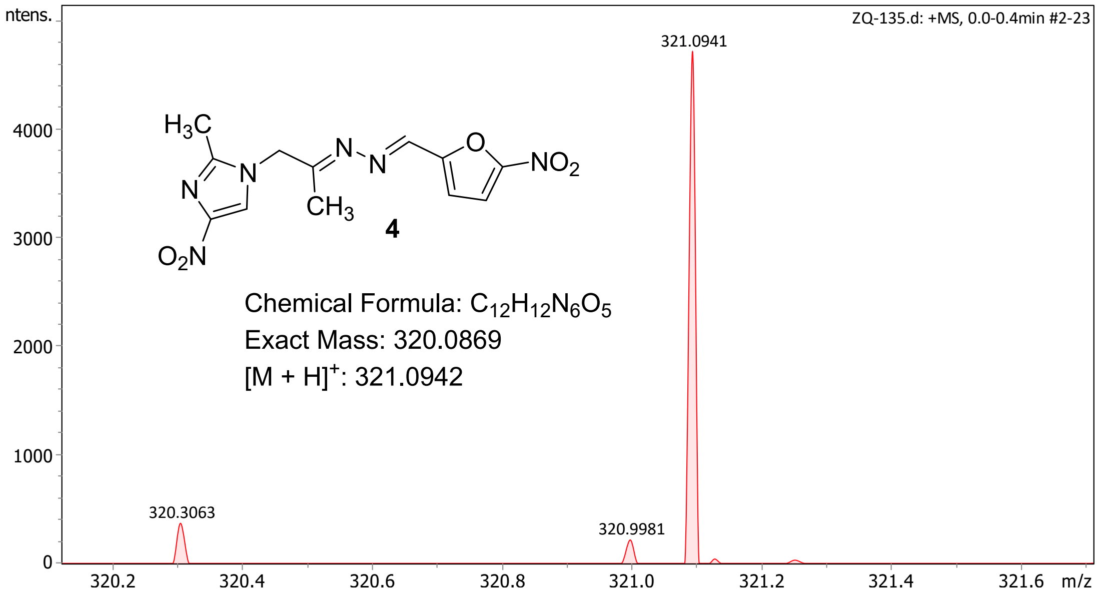

HPLC trace

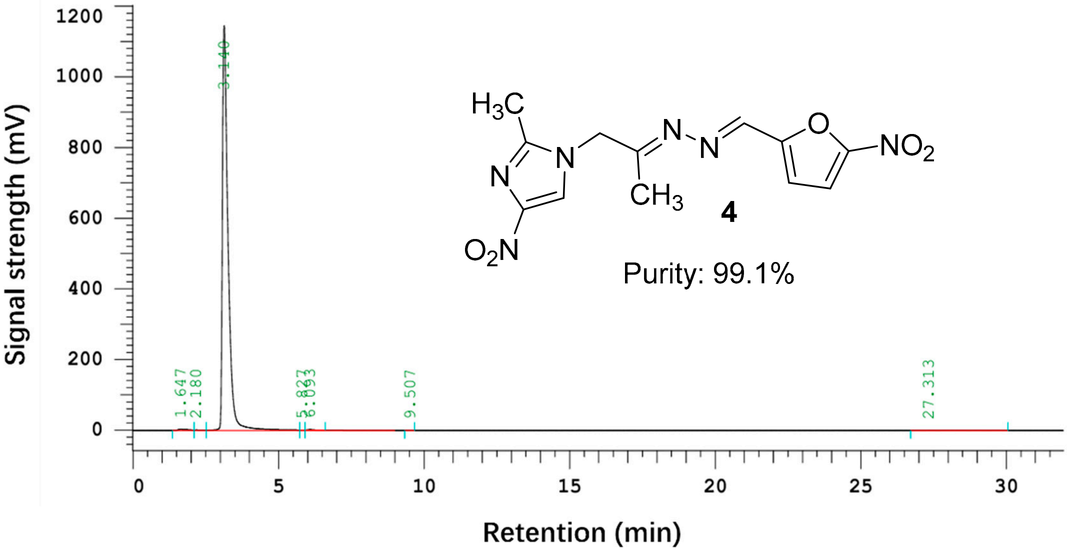

No.	RT	Area	Concentration	BC
1	1. 647	81824	0. 528	BV
2	2. 180	29445	0. 190	VV
3	3. 140	15350560	99. 146	VV
4	5. 827	922	0. 006	TBV
5	6. 093	16972	0. 110	TVB
6	9. 507	766	0. 005	BB
7	27. 313	2252	0. 015	BB
		15482741	100. 000	

*7.3. Spectra of compound **5a***

^{1}H NMR spectrum (400 MHz, 25 °C, DMSO-d_6)

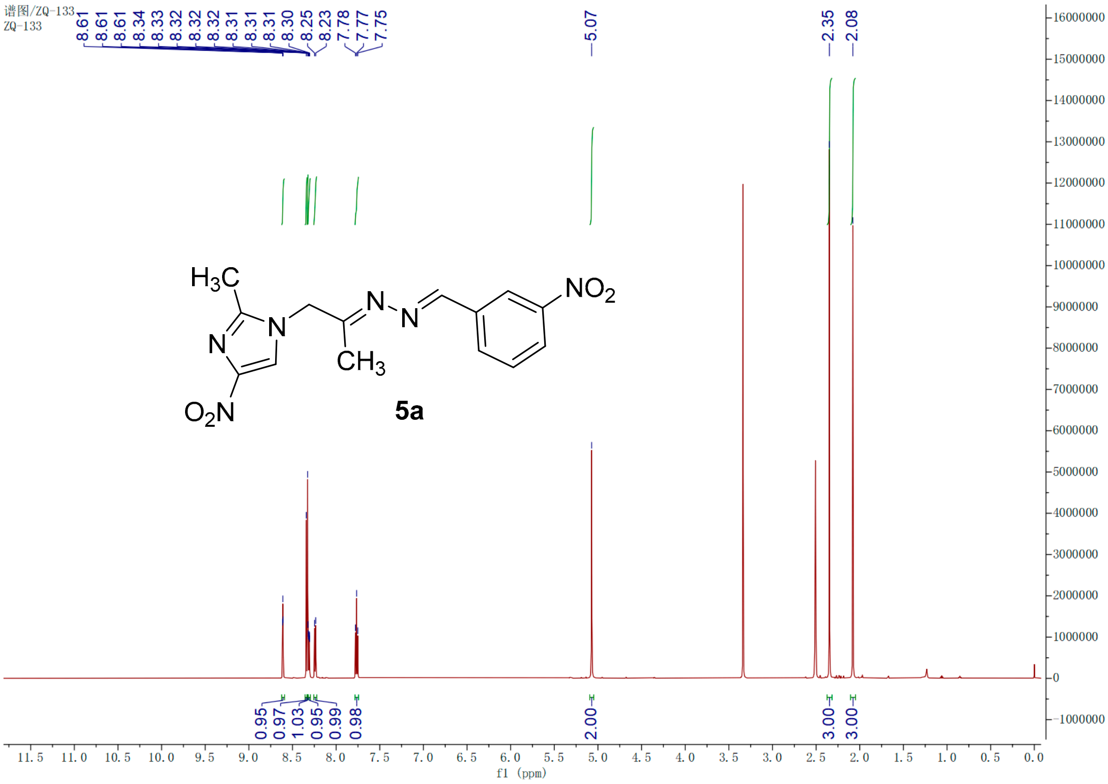

^{13}C NMR spectrum (101 MHz, 25 °C, DMSO-d_6)

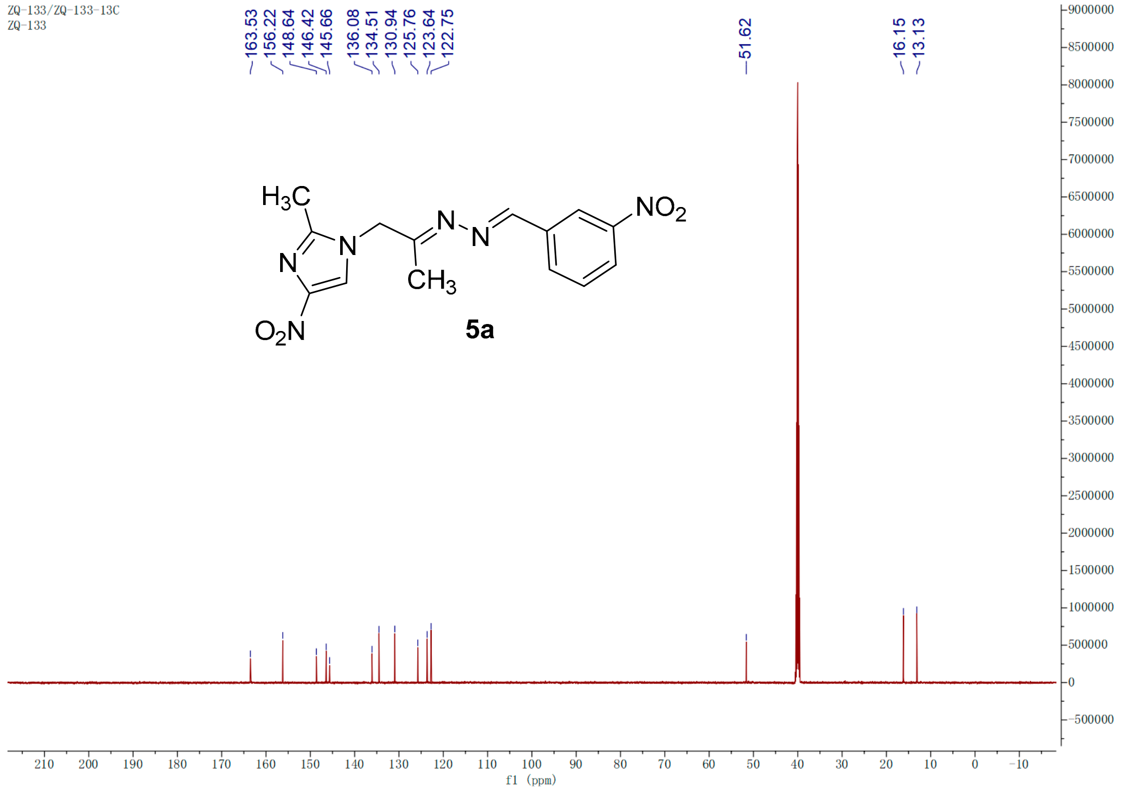

HRMS spectrum

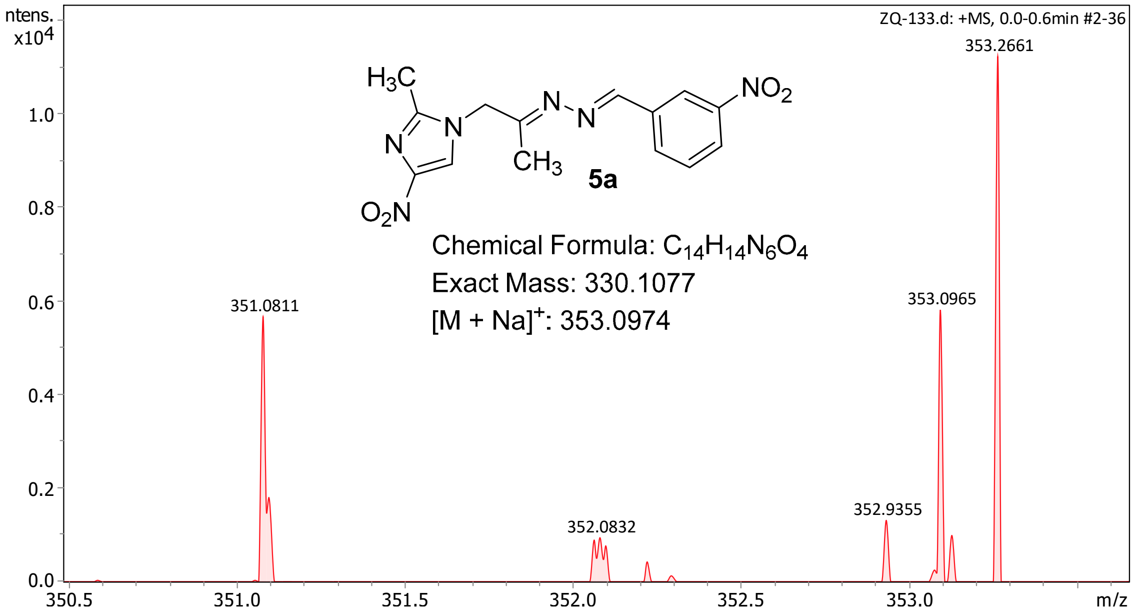

*7.4. Spectra of compound **5b***

^{1}H NMR spectrum (400 MHz, 25 °C, DMSO-d_6)

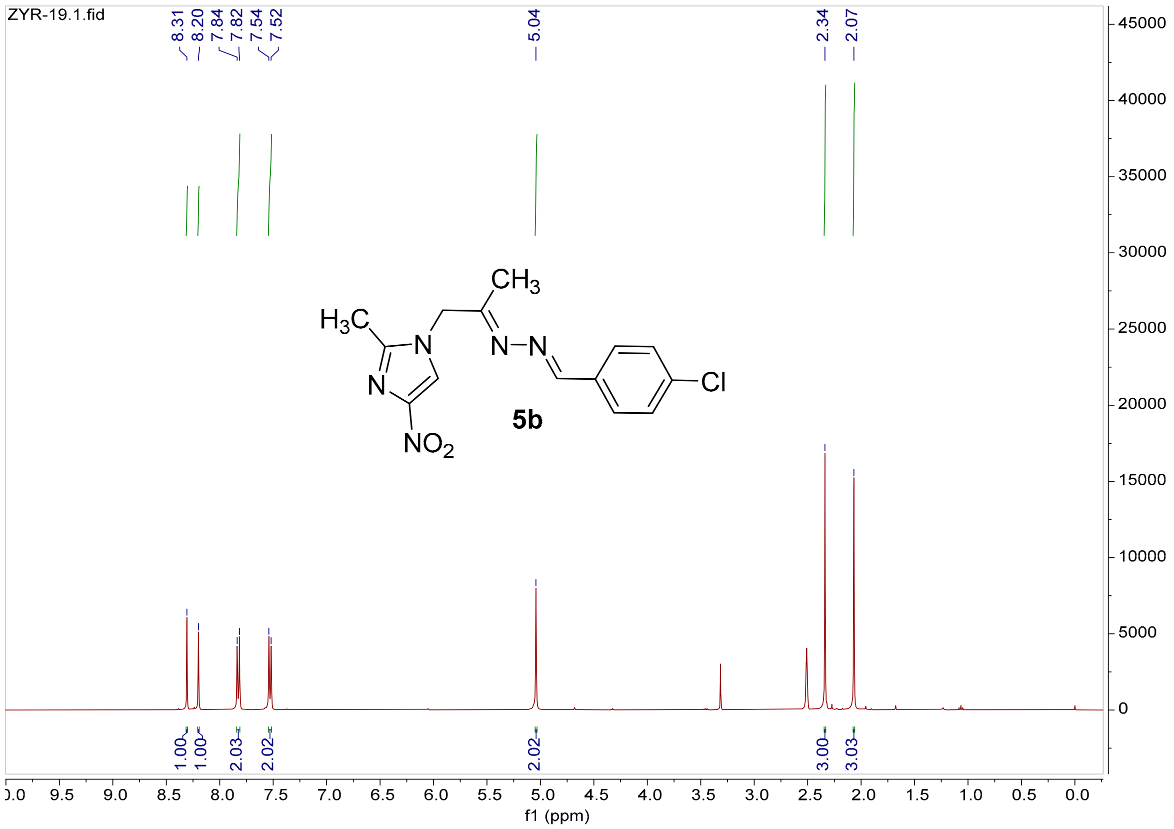

^{13}C NMR spectrum (101 MHz, 25 °C, DMSO-d_6)

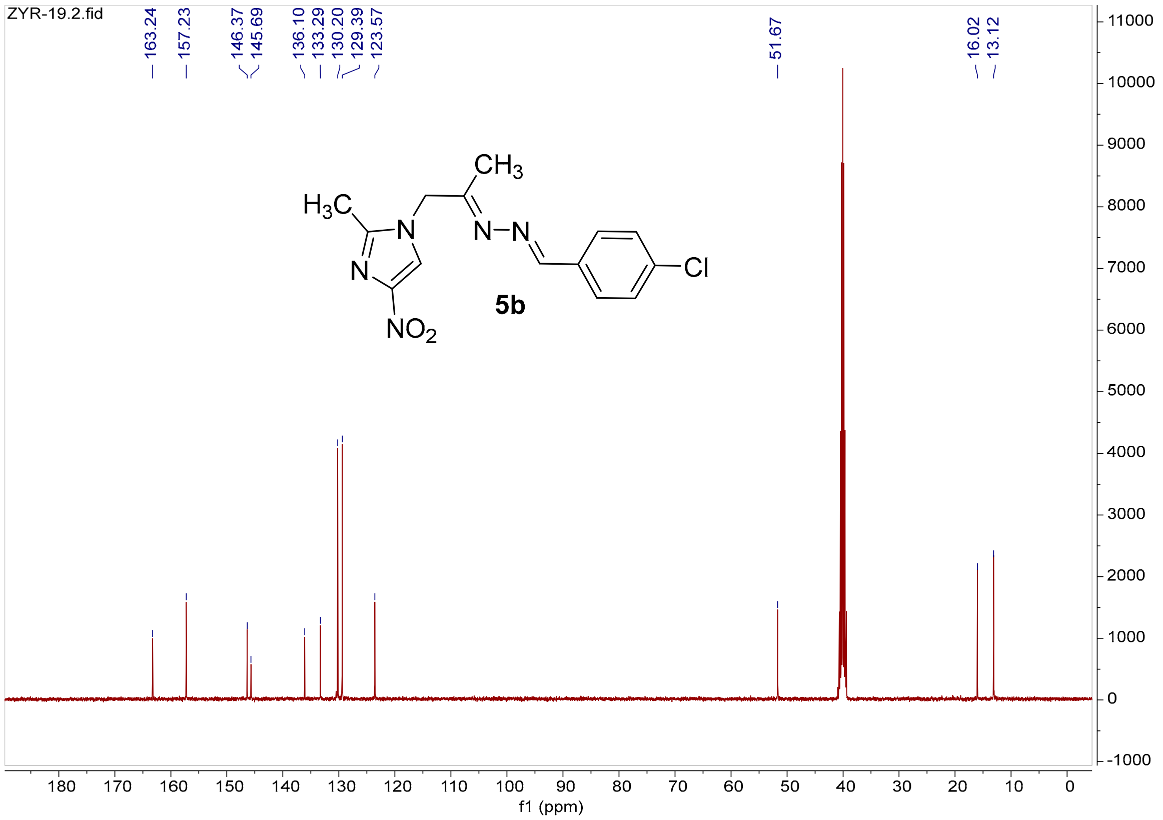

HRMS spectrum

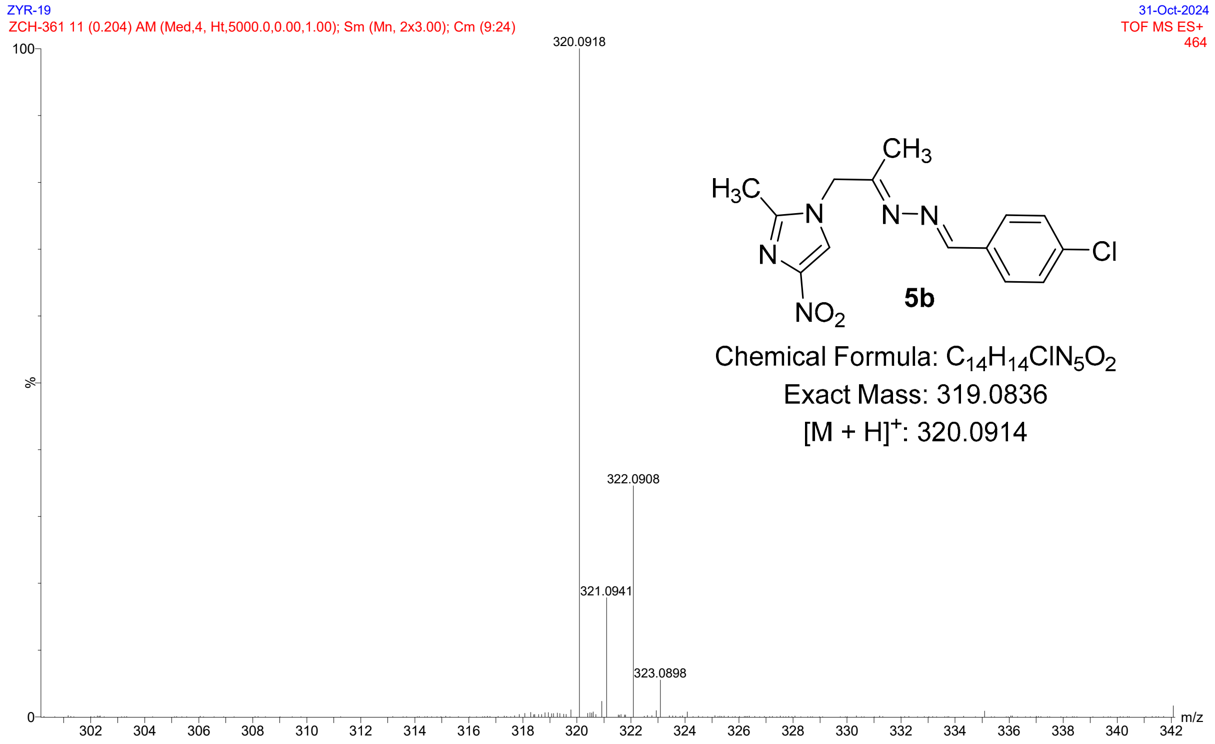

7.5. Spectra of compound ***5c***

^{1}H NMR spectrum (400 MHz, 25 °C, DMSO-d_6)

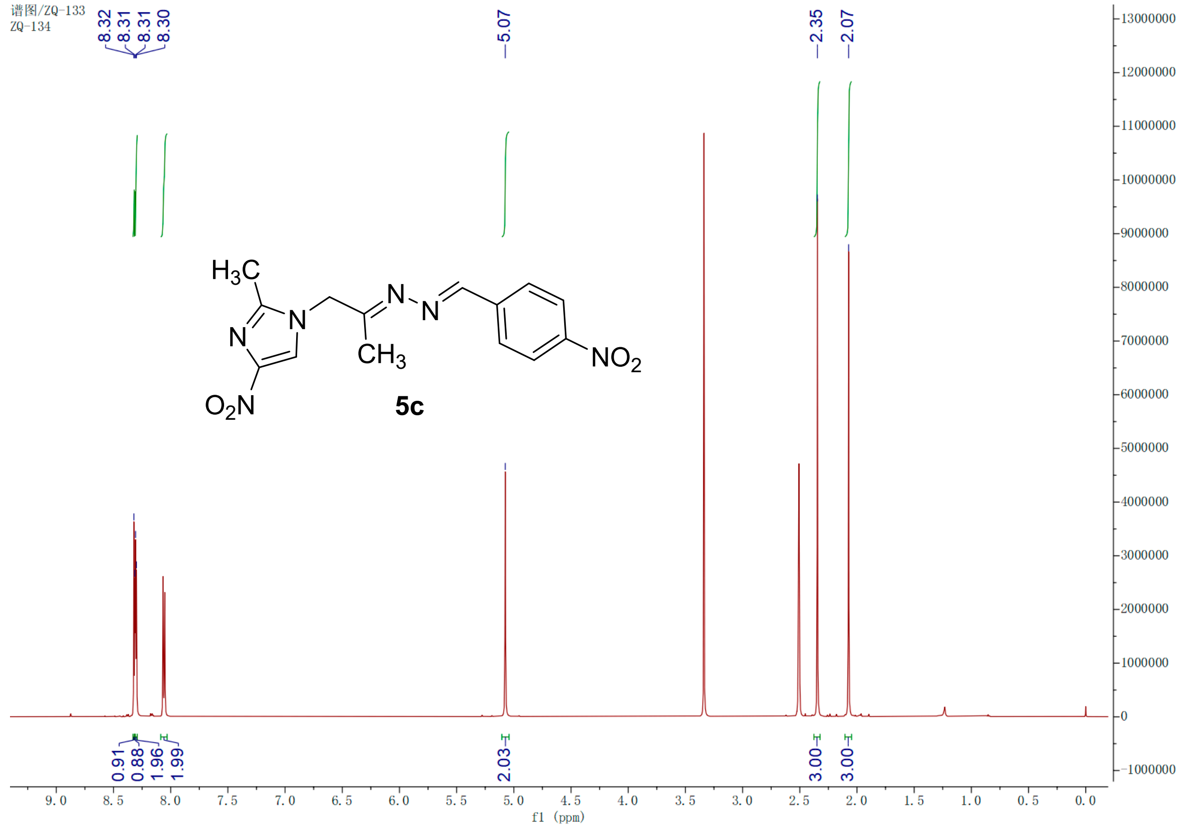

^{13}C NMR spectrum (101 MHz, 25 °C, DMSO-d_6)

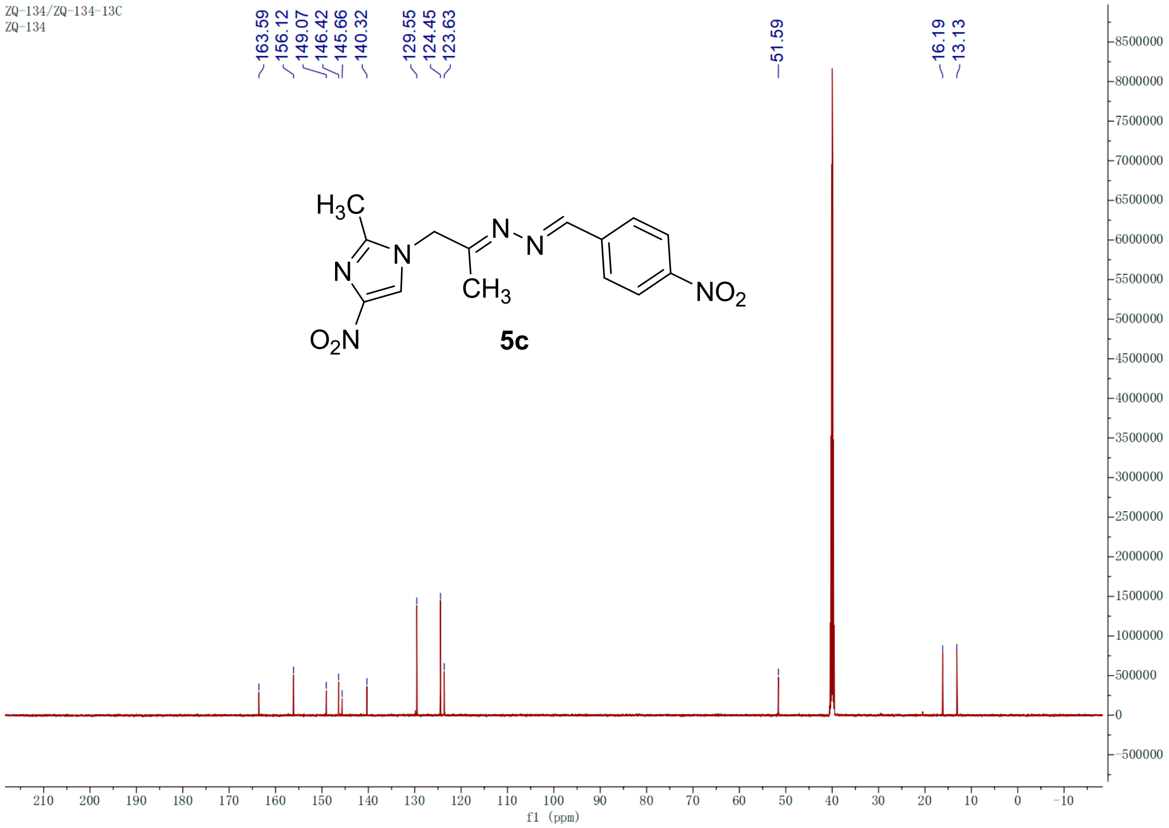

HRMS spectrum

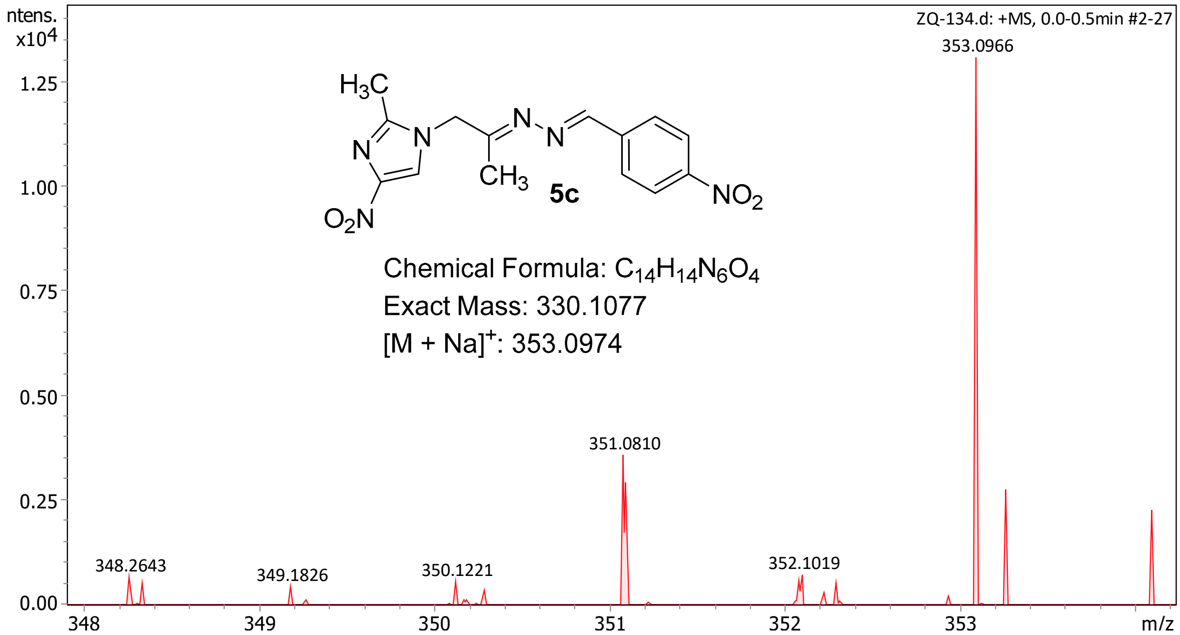

*7.6. Spectra of compound **7***

^{1}H NMR spectrum (400 MHz, 25 °C, DMSO-d_6)

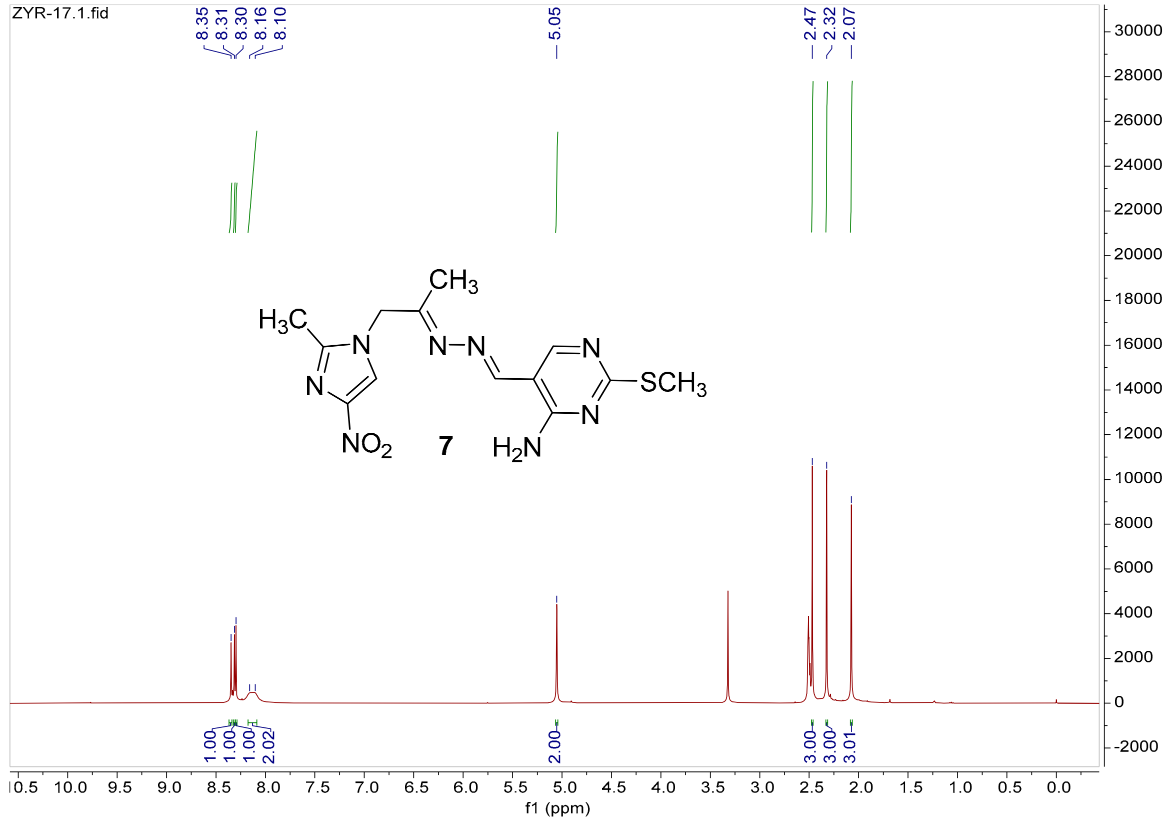

^{13}C NMR spectrum (101 MHz, 25 °C, DMSO-d_6)

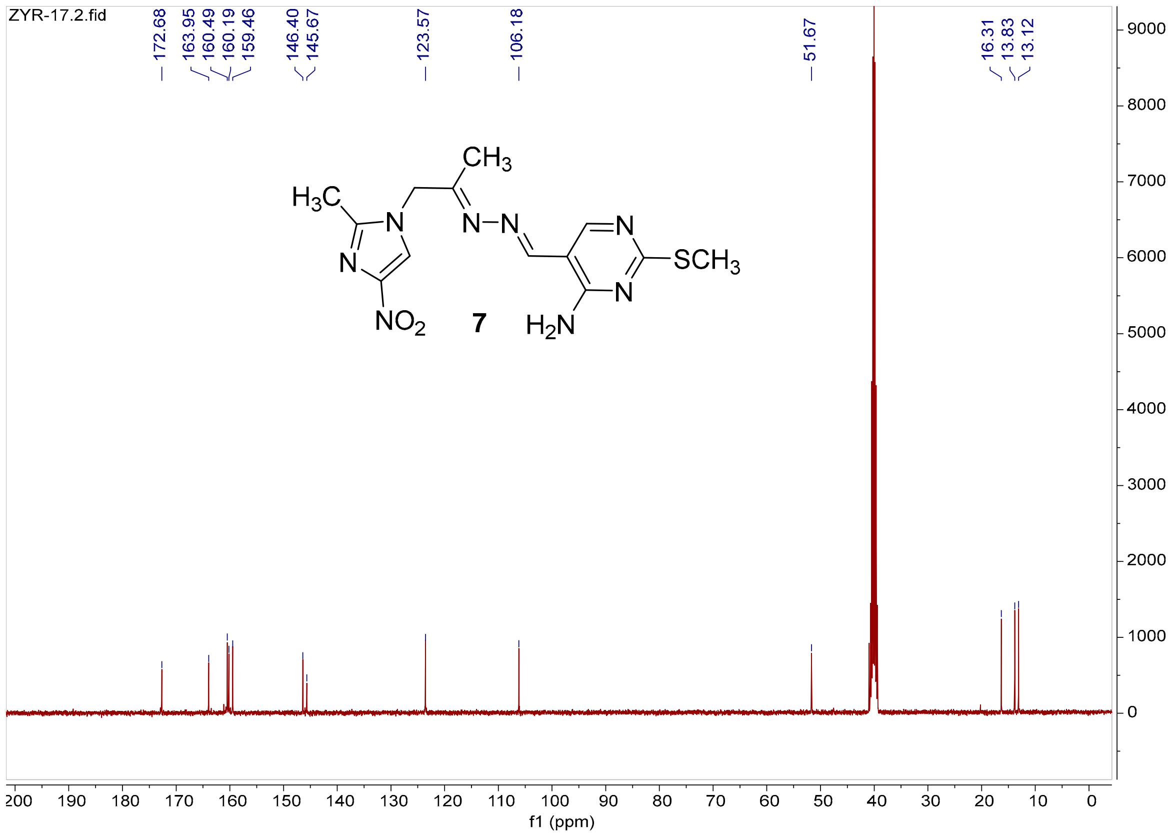

HRMS spectrum

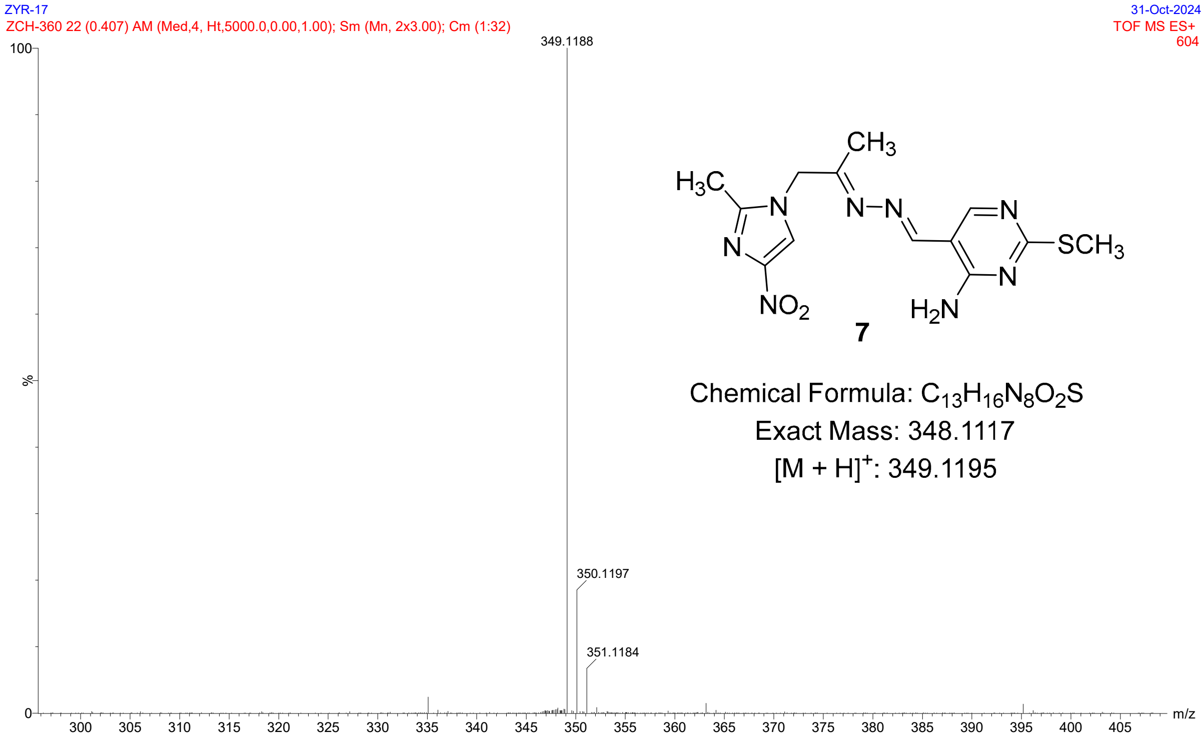

*7.7. Spectra of compound **8***

^{1}H NMR spectrum (400 MHz, 25 °C, DMSO-d_6)

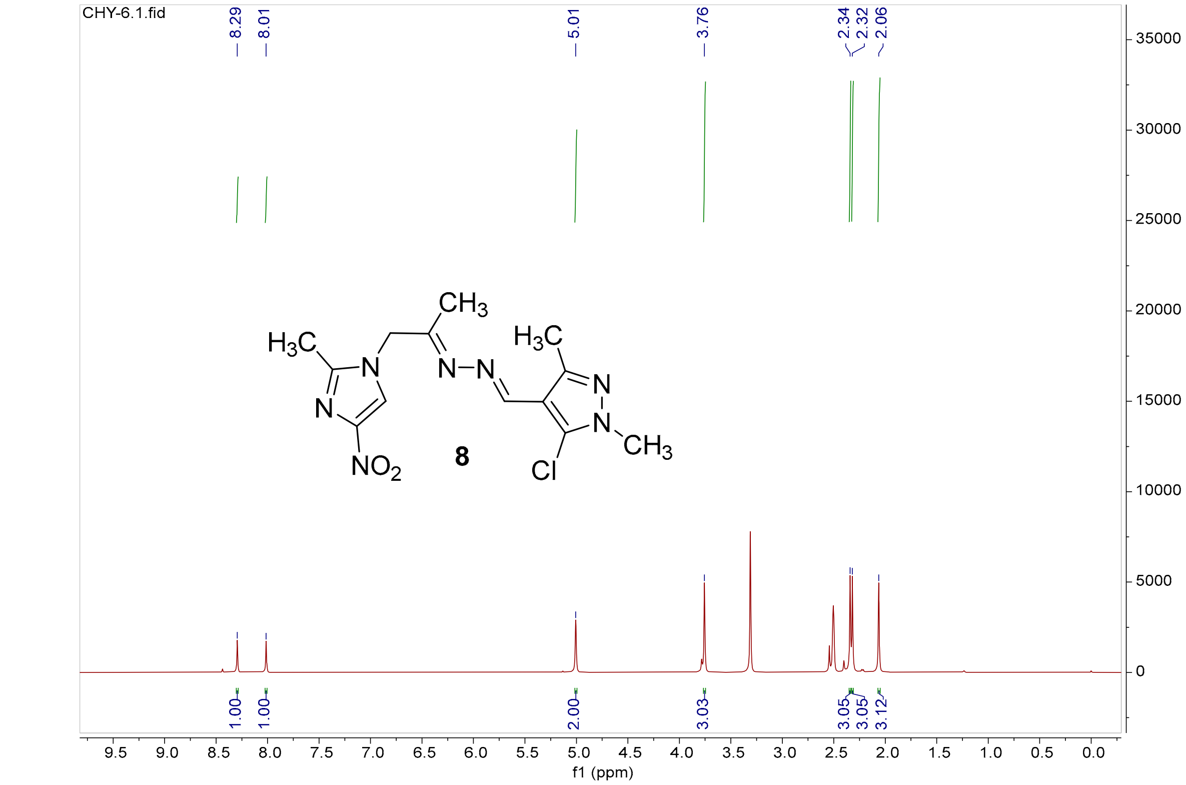

^{13}C NMR spectrum (101 MHz, 25 °C, DMSO-d_6)

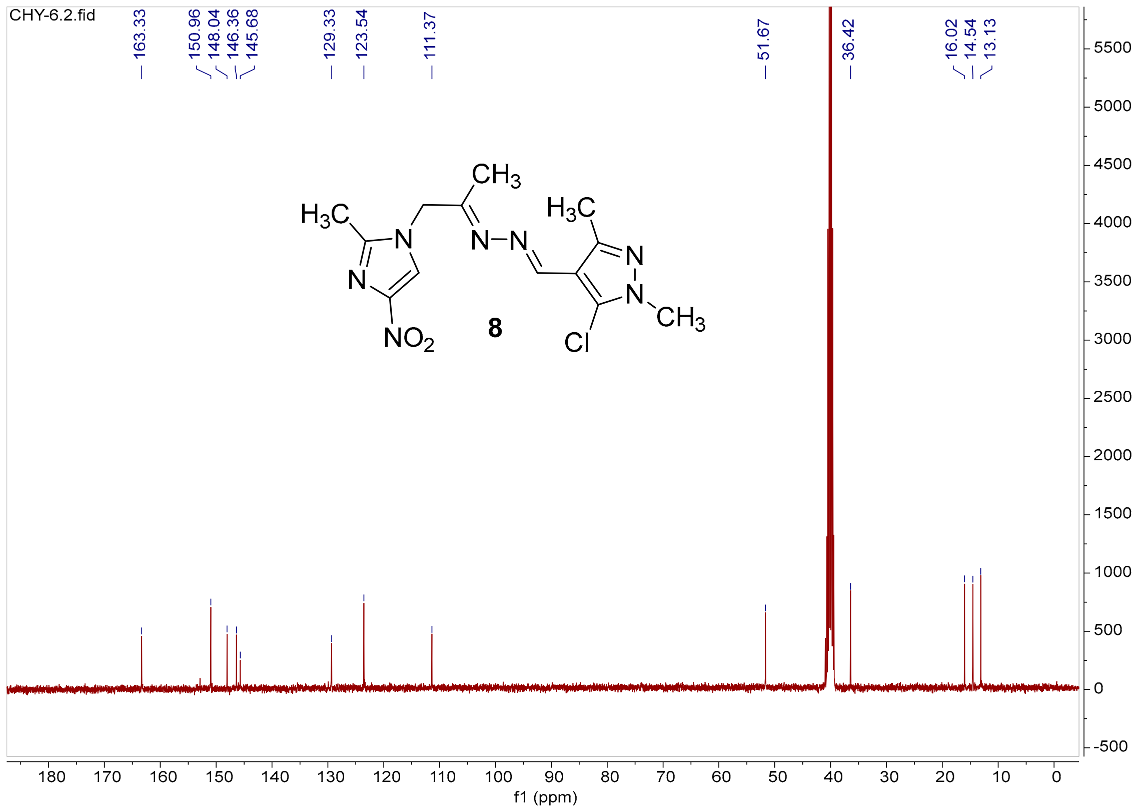

HRMS spectrum

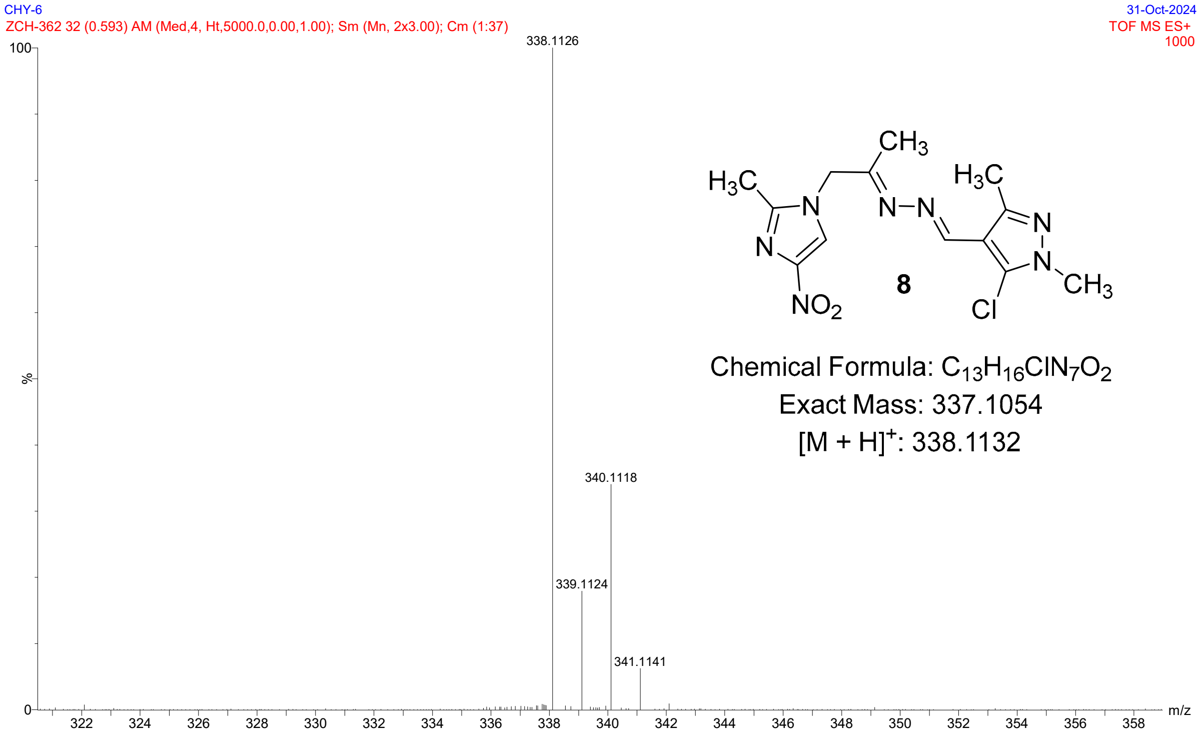

*7.8. Spectra of compound **10a***

^{1}H NMR spectrum (600 MHz, 25 °C, DMSO-d_6)

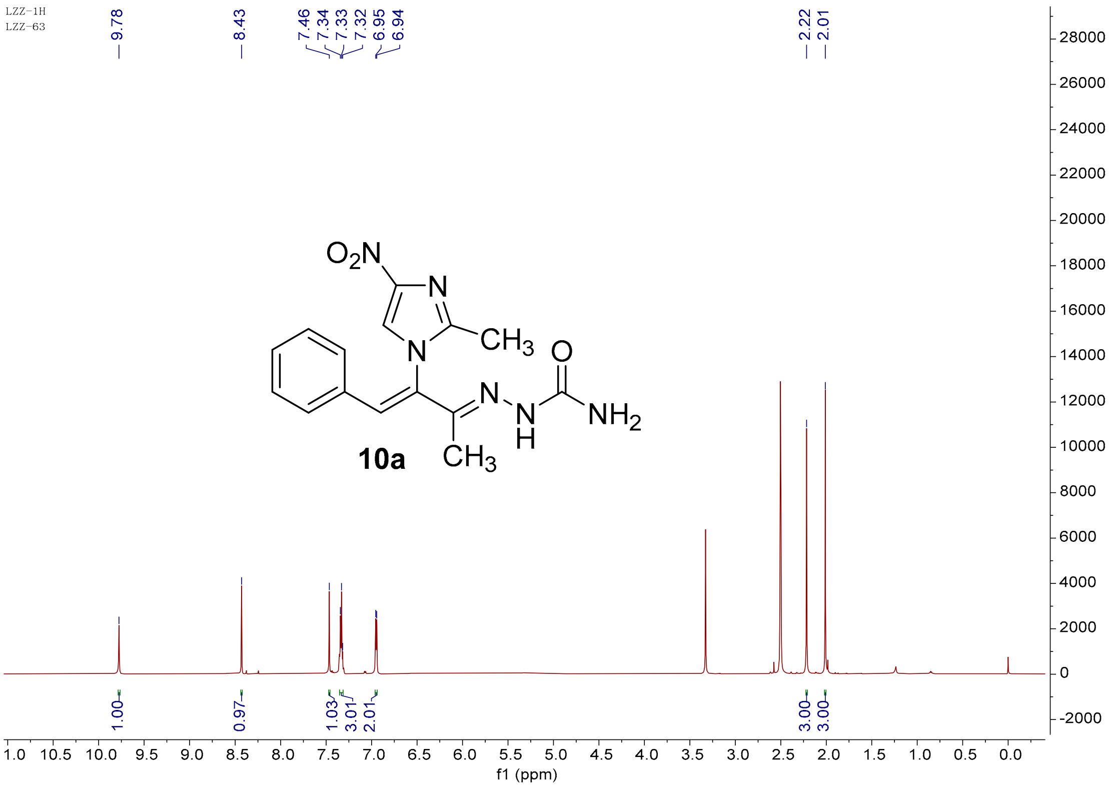

^{13}C NMR spectrum (151 MHz, 25 °C, DMSO-d_6)

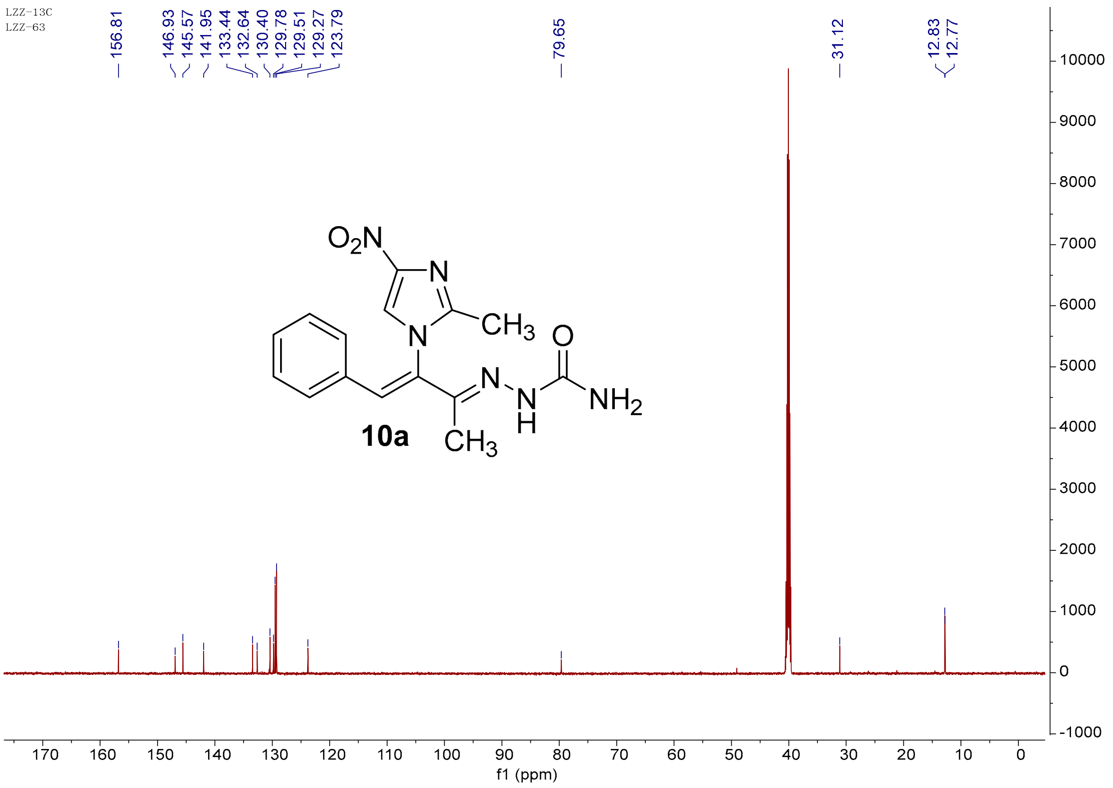

HRMS spectrum

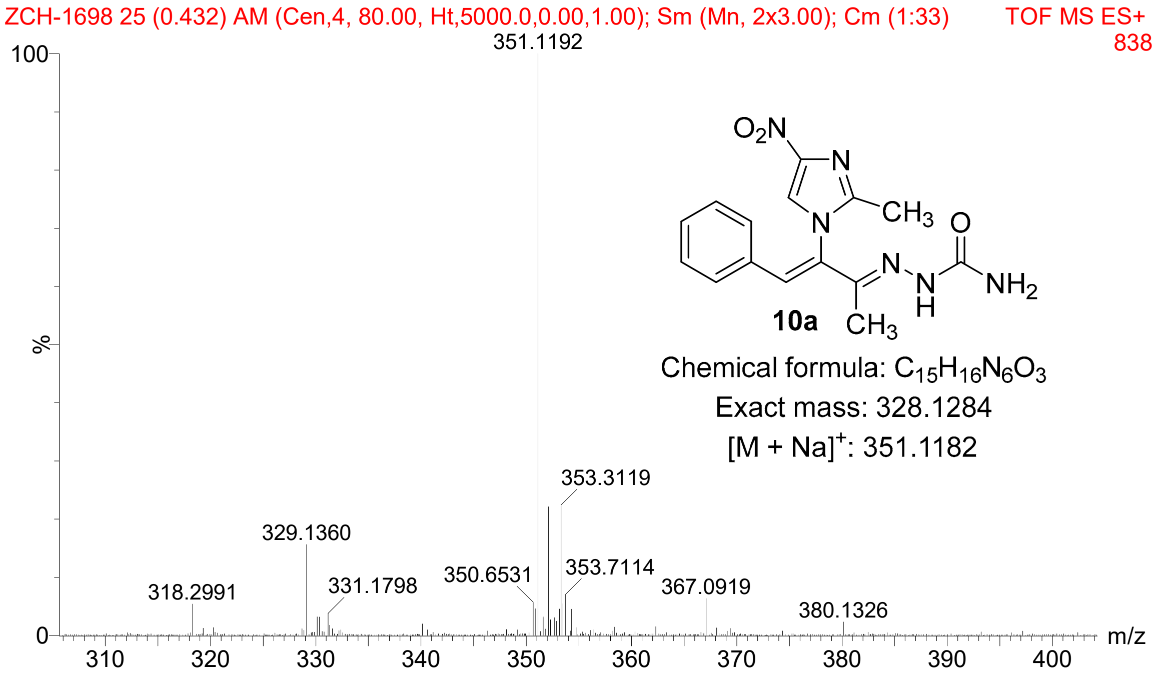

*7.9. Spectra of compound **10b***

^{1}H NMR spectrum (600 MHz, 25 °C, DMSO-d_6)

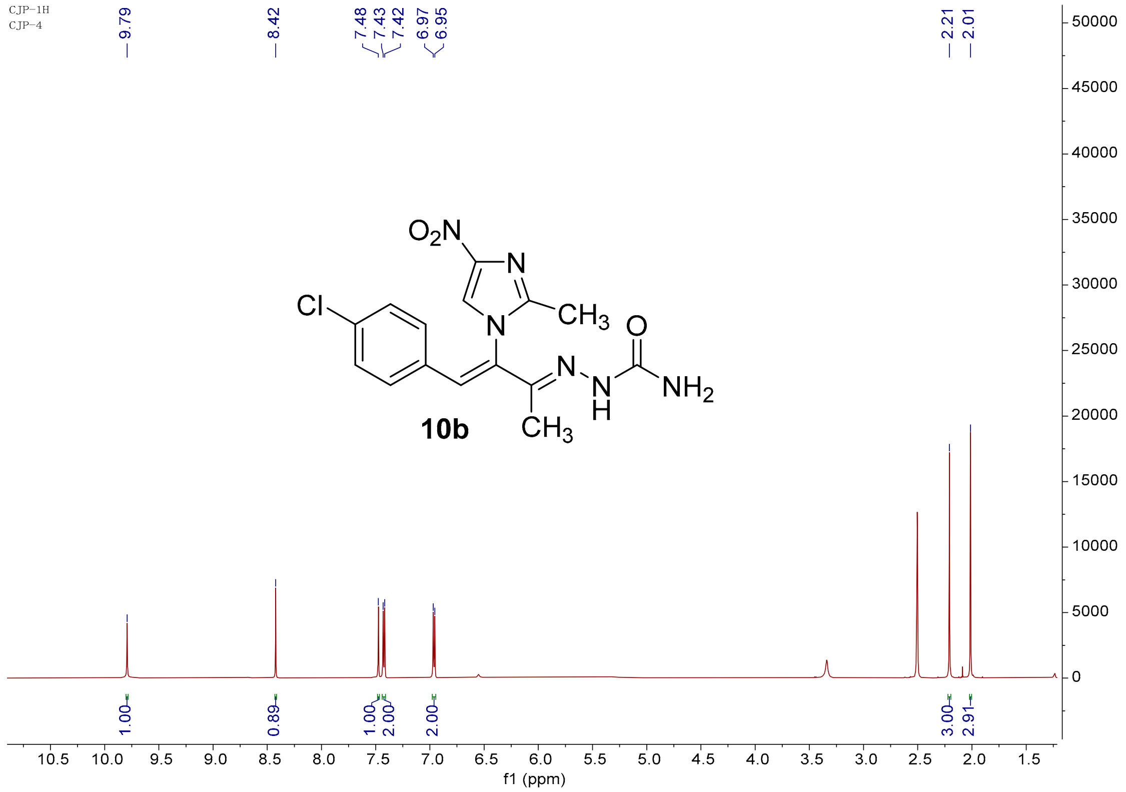

^{13}C NMR spectrum (151 MHz, 25 °C, DMSO-d_6)

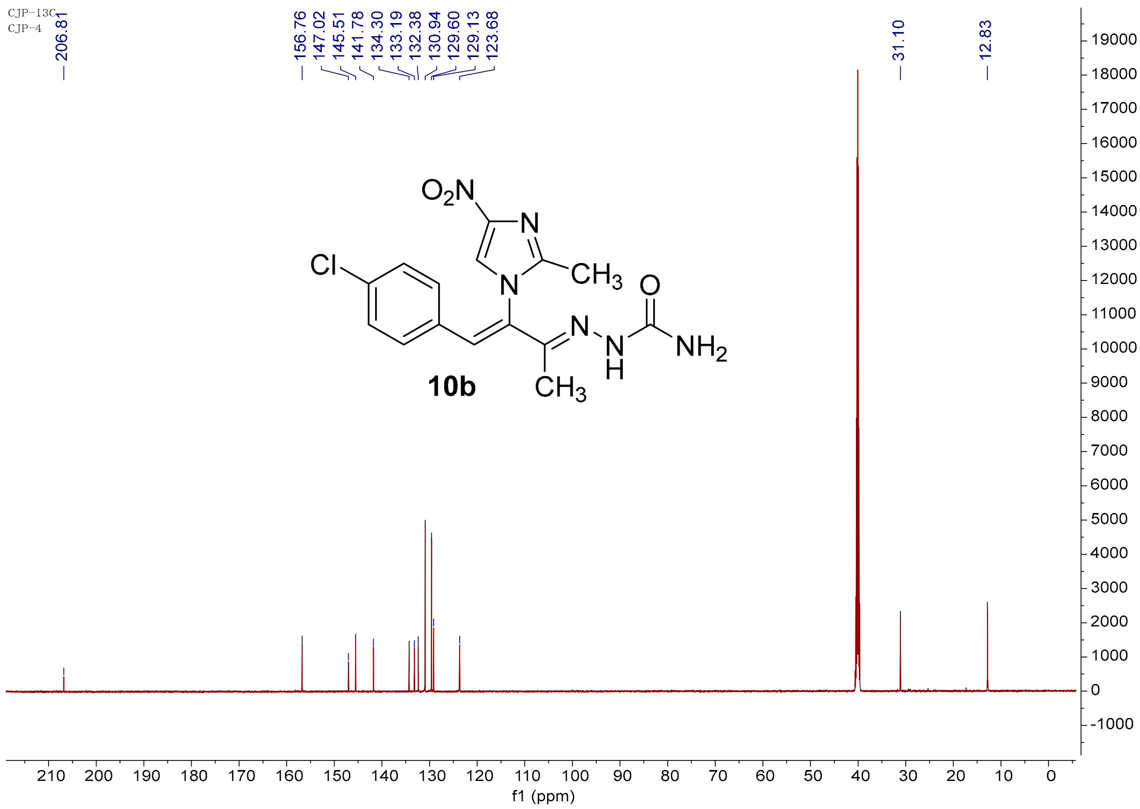

HRMS spectrum

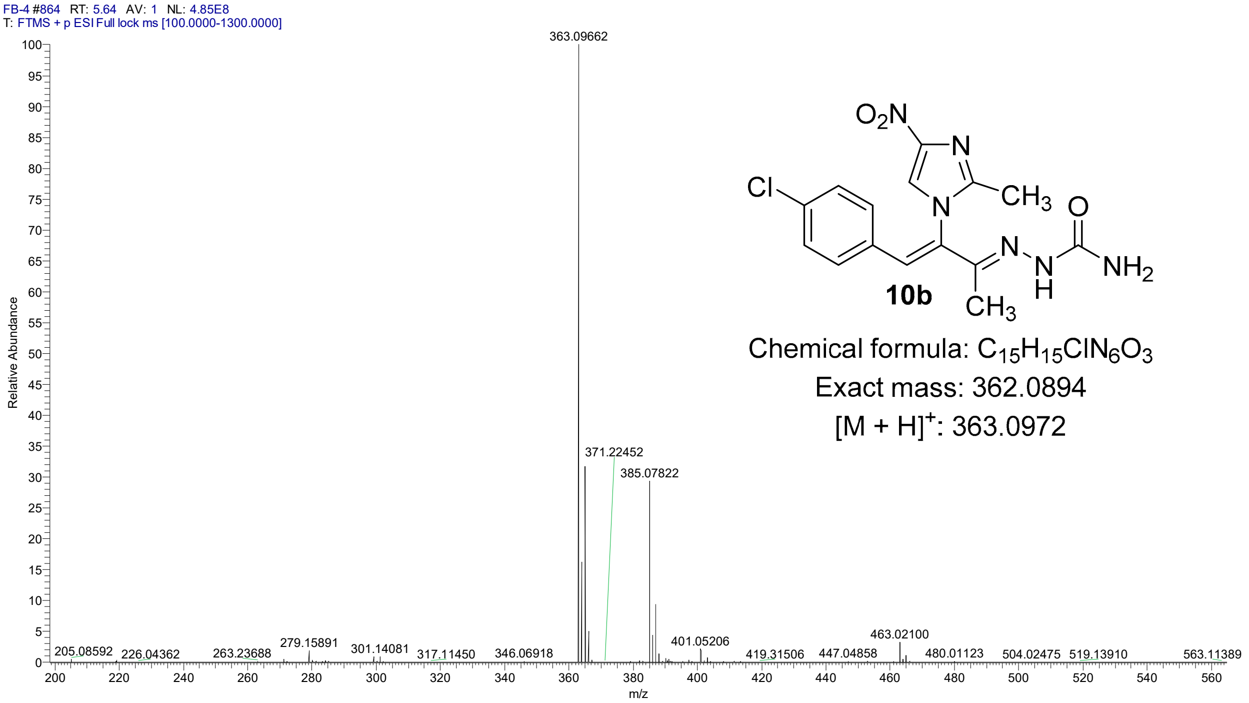

*7.10. Spectra of compound **11b***

^{1}H NMR spectrum (600 MHz, 25 °C, DMSO-d_6)

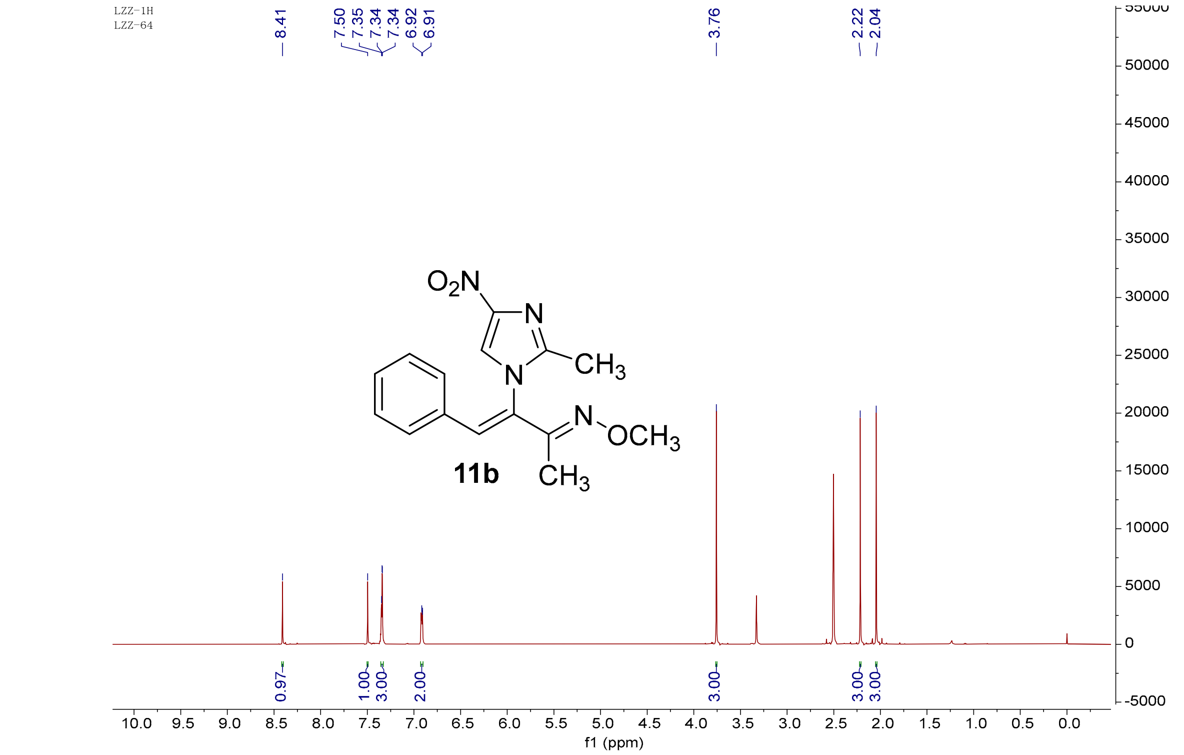

^{13}C NMR spectrum (151 MHz, 25 °C, DMSO-d_6)

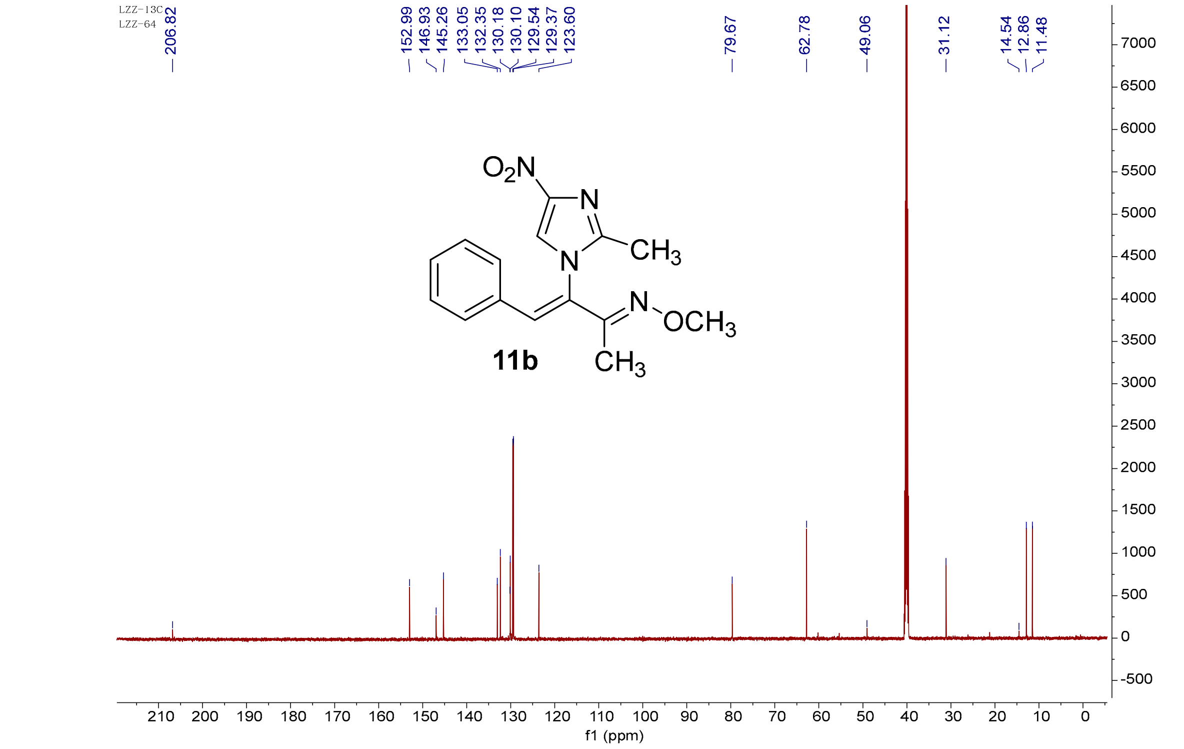

HRMS spectrum

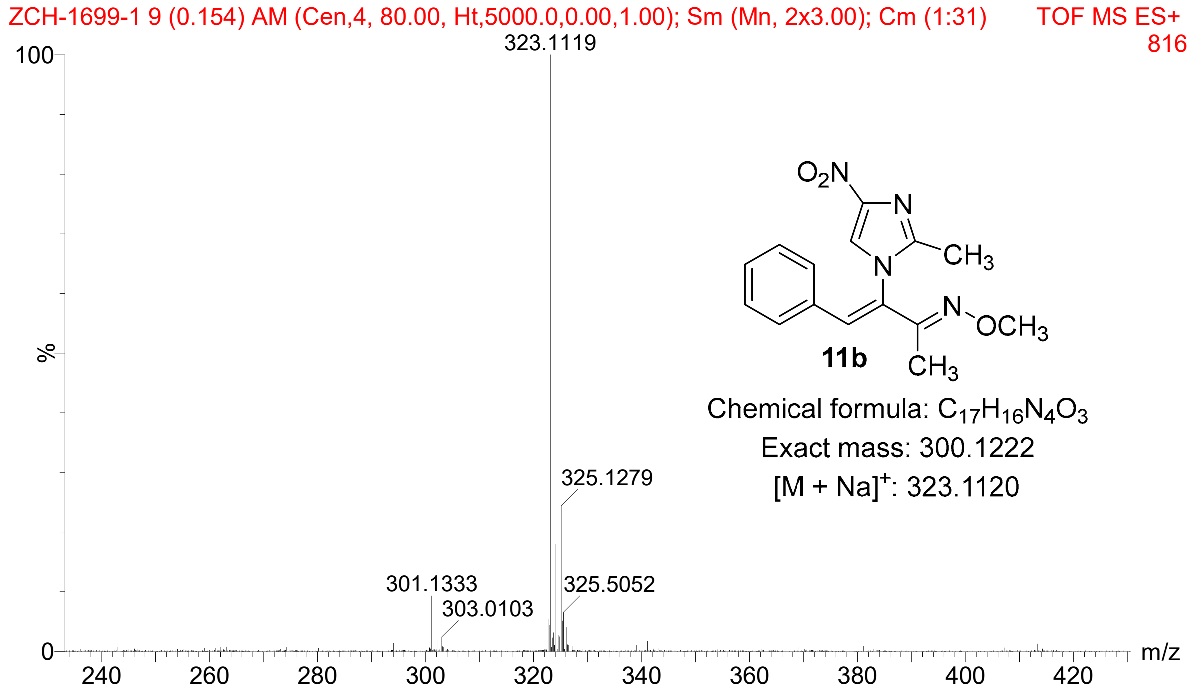

7.11. Spectra of compound **13b**

^{1}H NMR spectrum (600 MHz, 25 °C, DMSO-d_6)

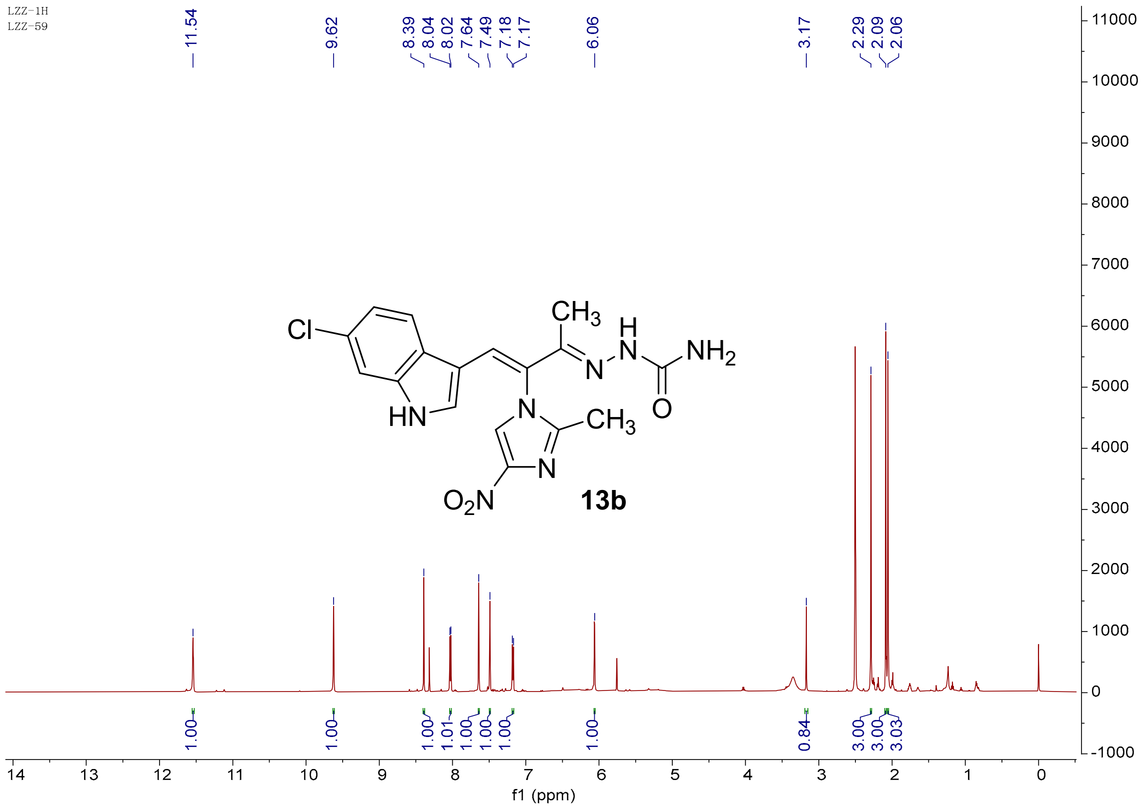

^{13}C NMR spectrum (151 MHz, 25 °C, DMSO-d_6)

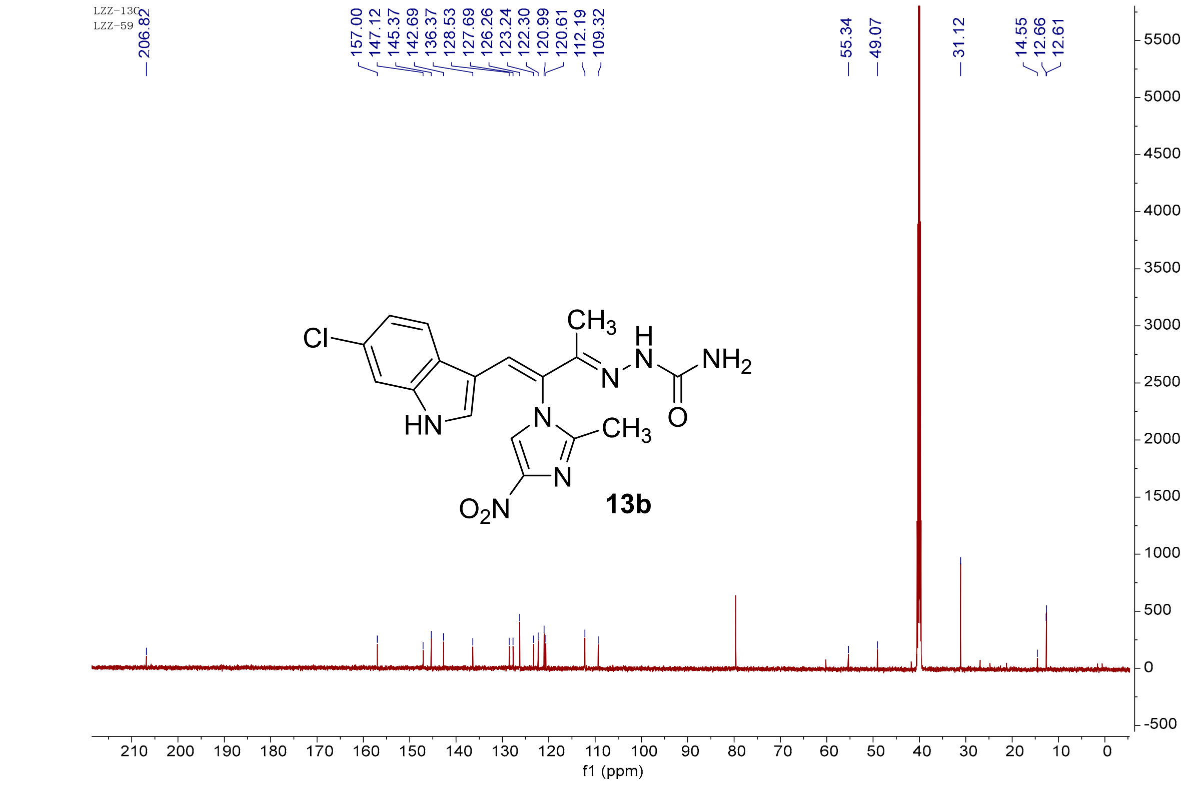

HRMS spectrum

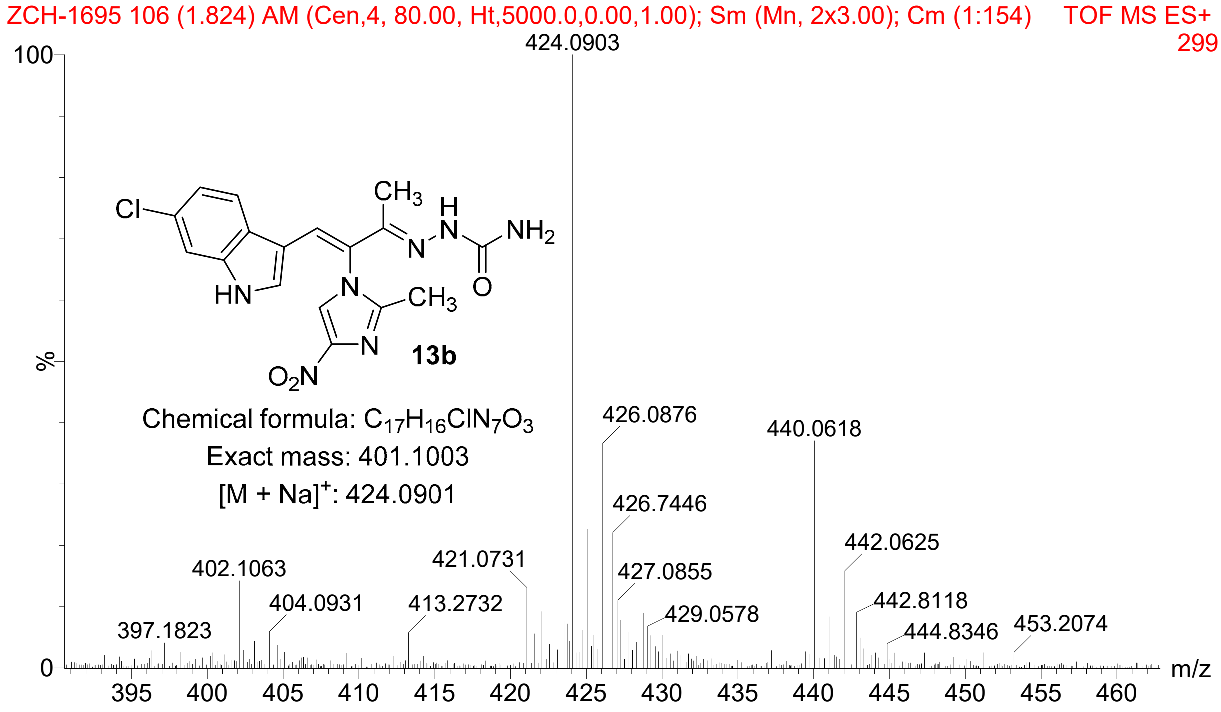

*7.12. Spectra of compound **14a***

^{1}H NMR spectrum (600 MHz, 25 °C, DMSO-d_6)

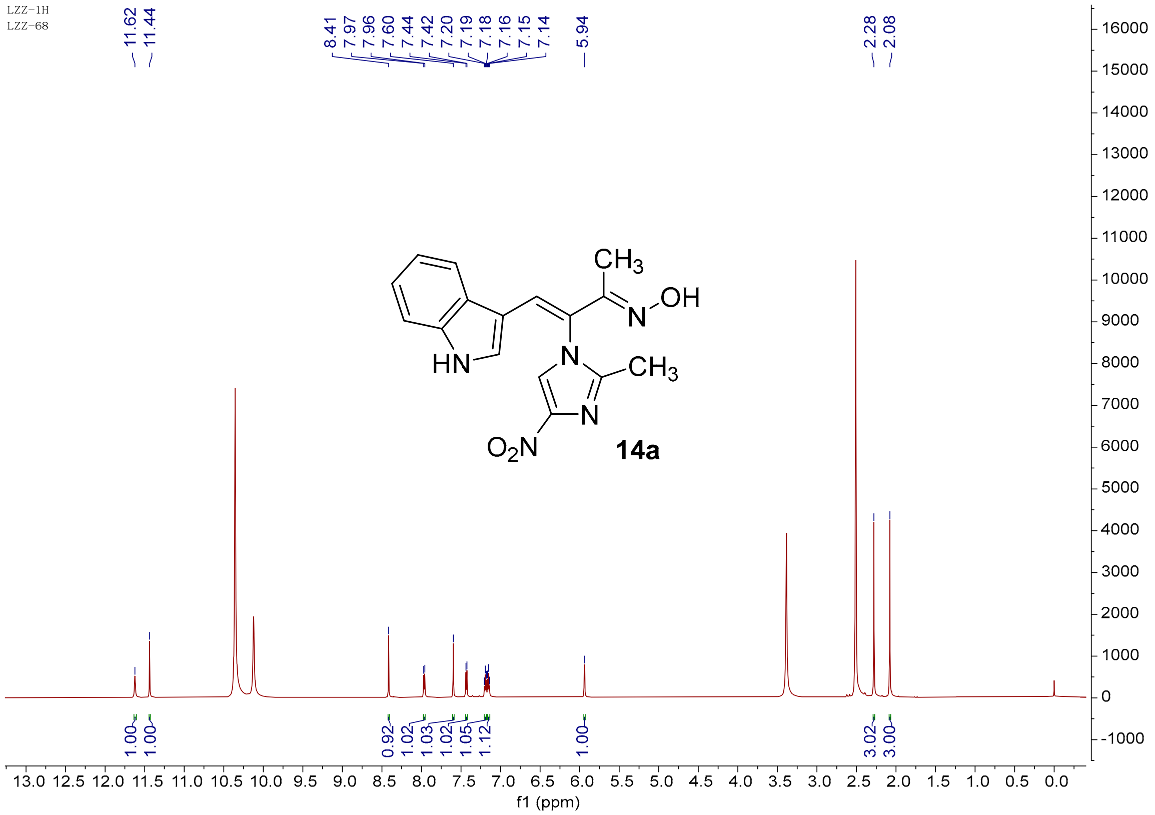

^{13}C NMR spectrum (101 MHz, 25 °C, DMSO-d_6)

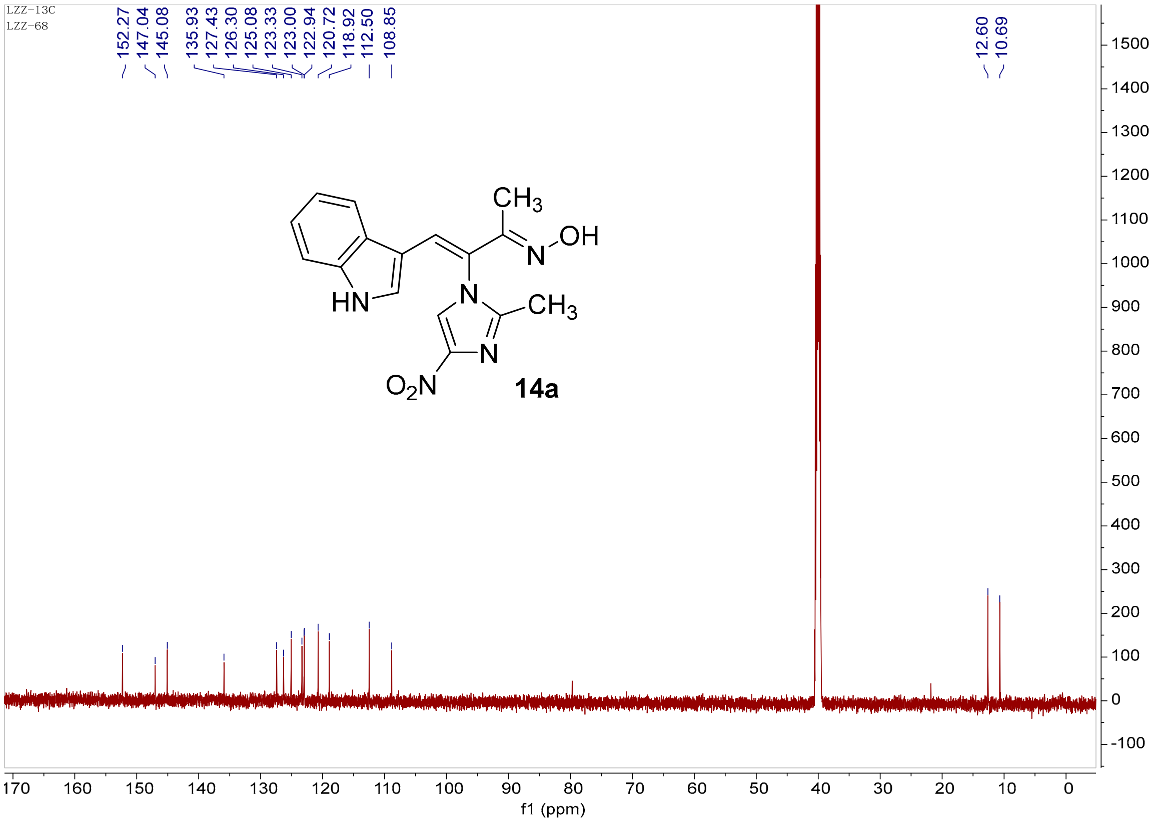

HRMS spectrum

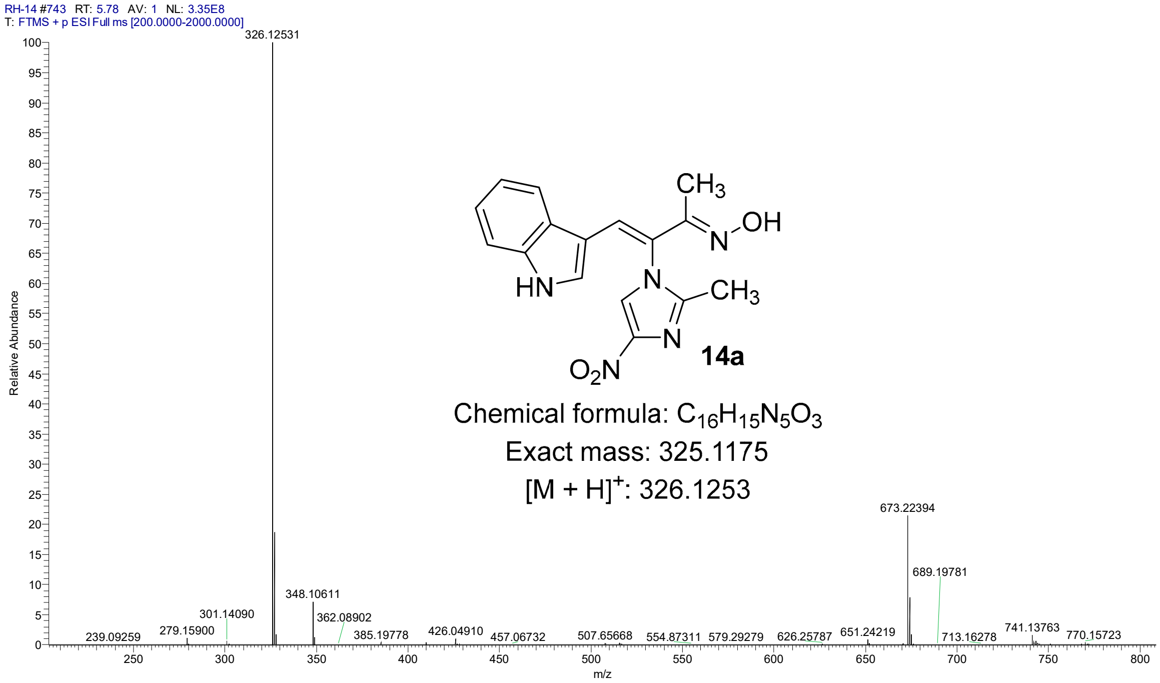

7.13. Spectra of compound **14b**

^{1}H NMR spectrum (600 MHz, 25 °C, DMSO-d_6)

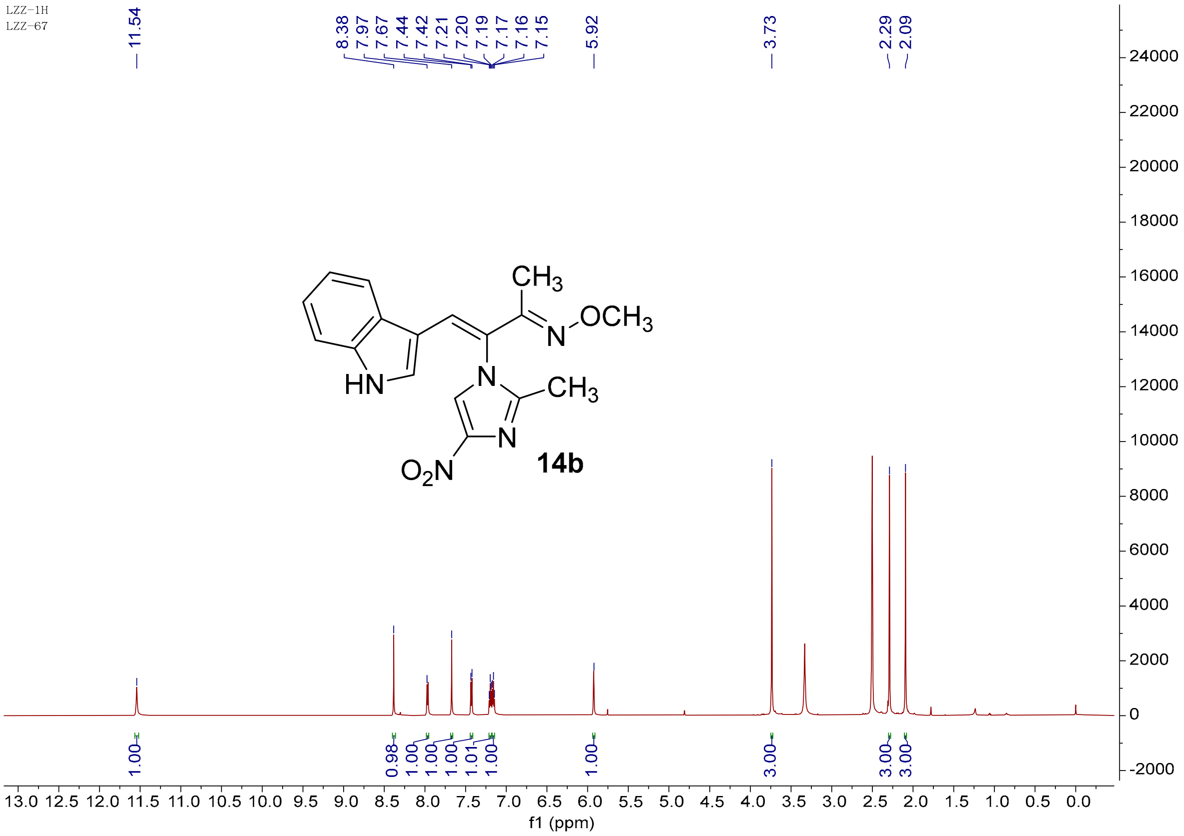

^{13}C NMR spectrum (151 MHz, 25 °C, DMSO-d_6)

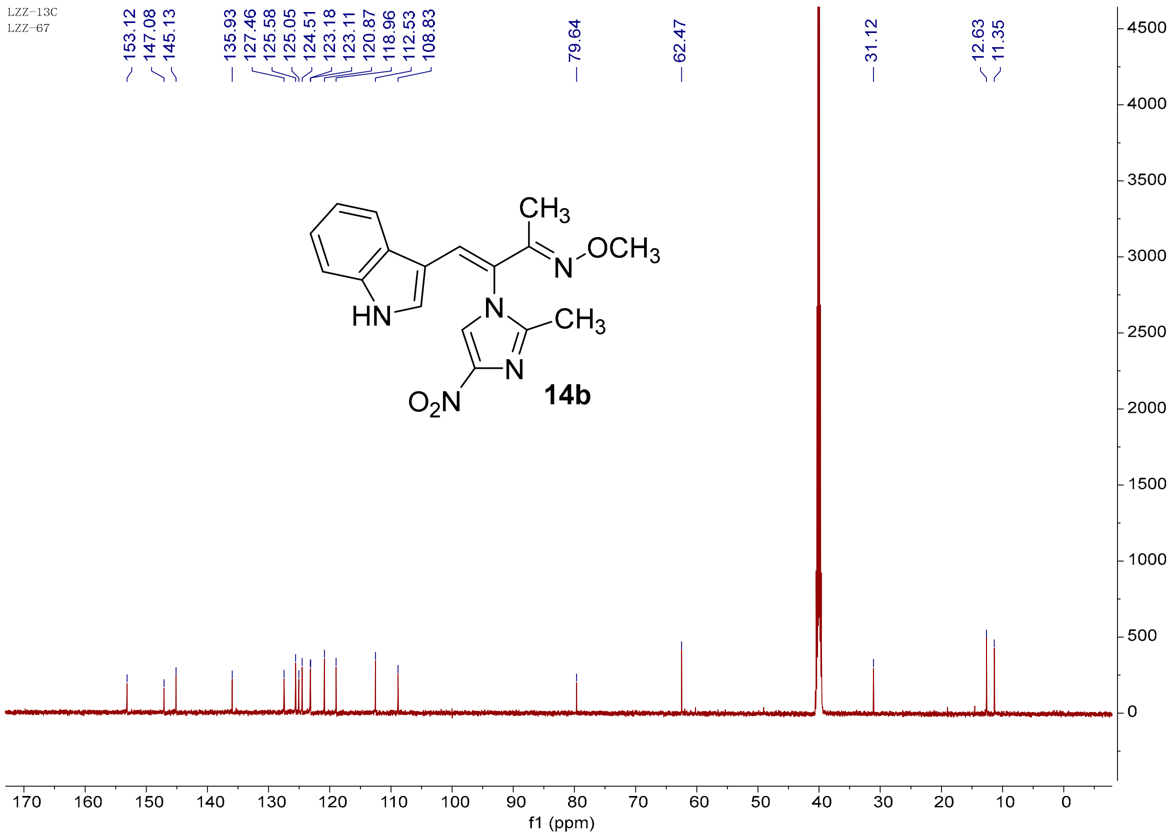

HRMS spectrum

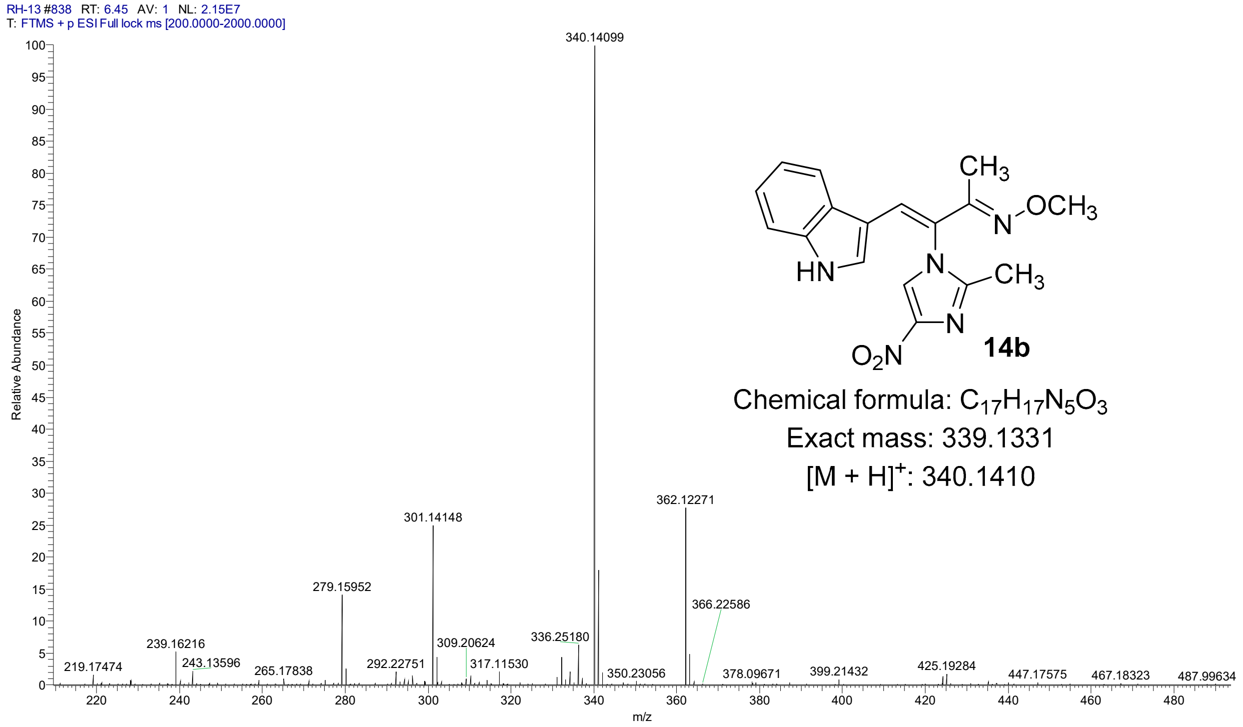

*7.14. Spectra of compound **14d***

^{1}H NMR spectrum (600 MHz, 25 °C, DMSO-d_6)

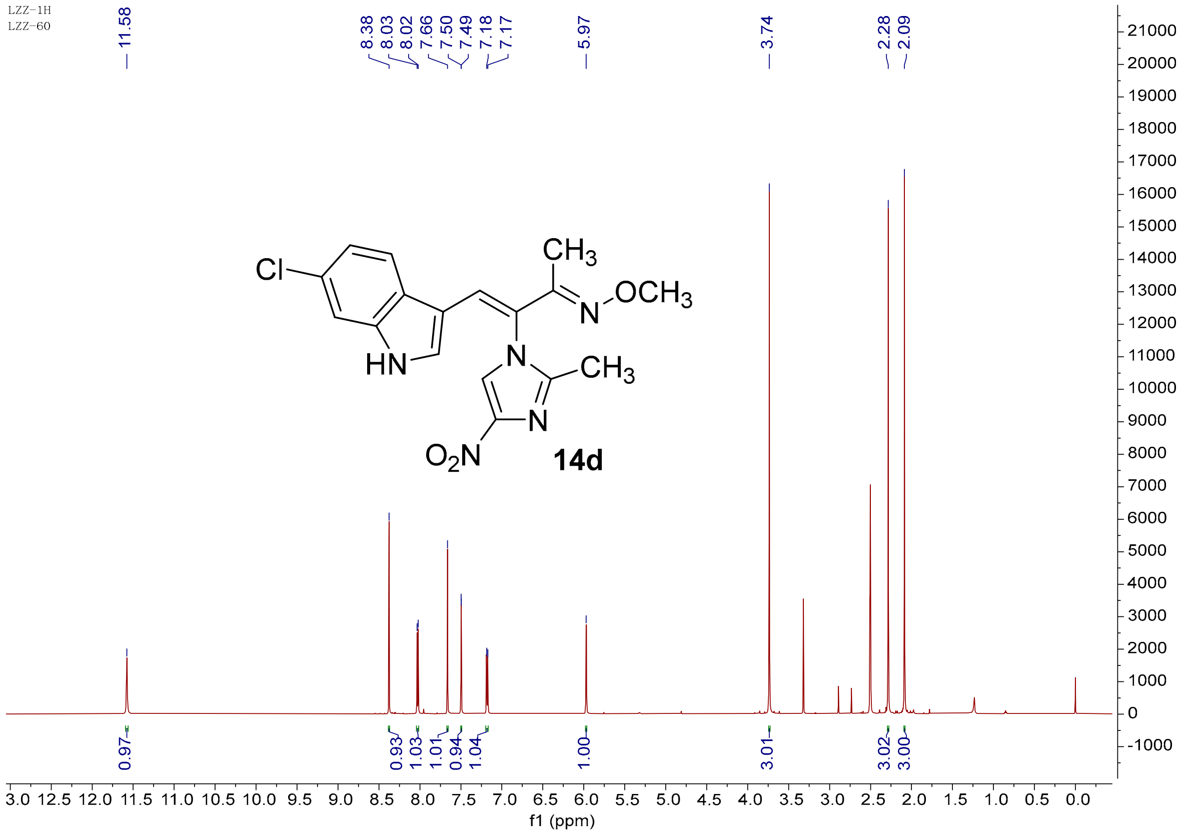

^{13}C NMR spectrum (101 MHz, 25 °C, DMSO-d_6)

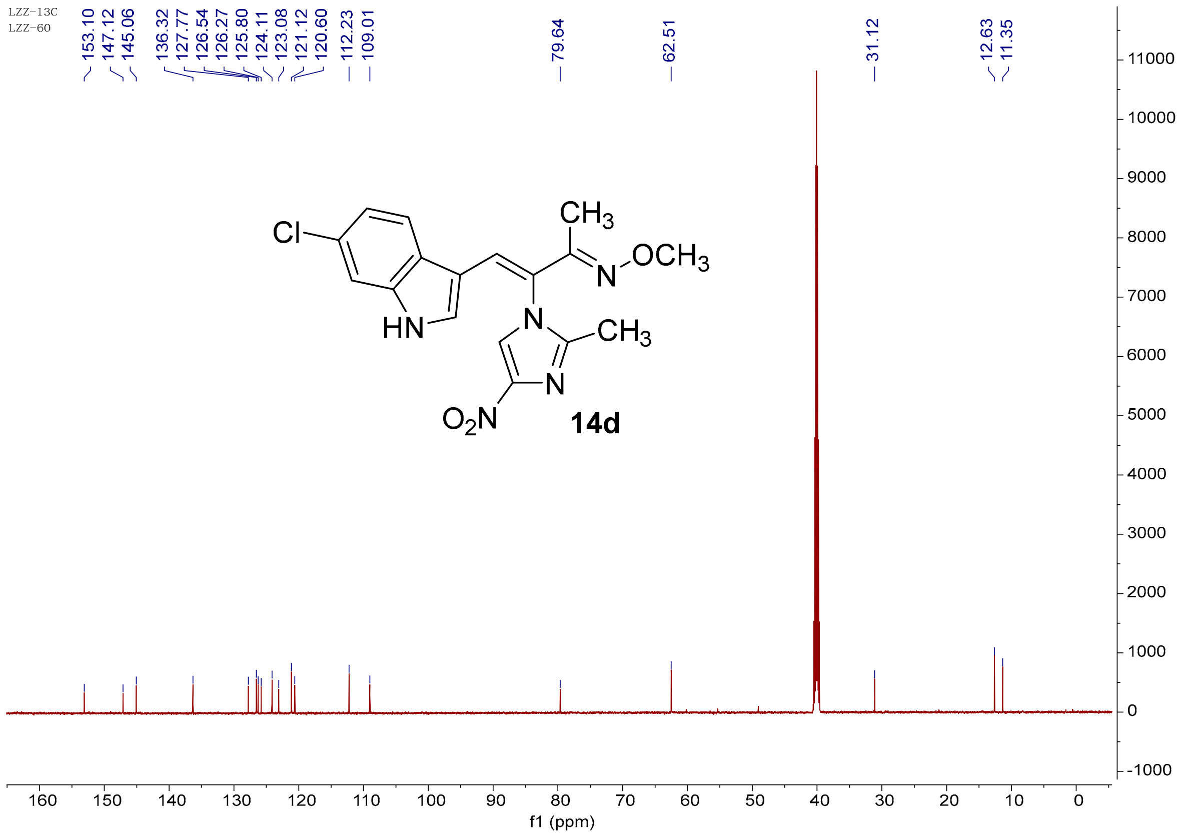

HRMS spectrum

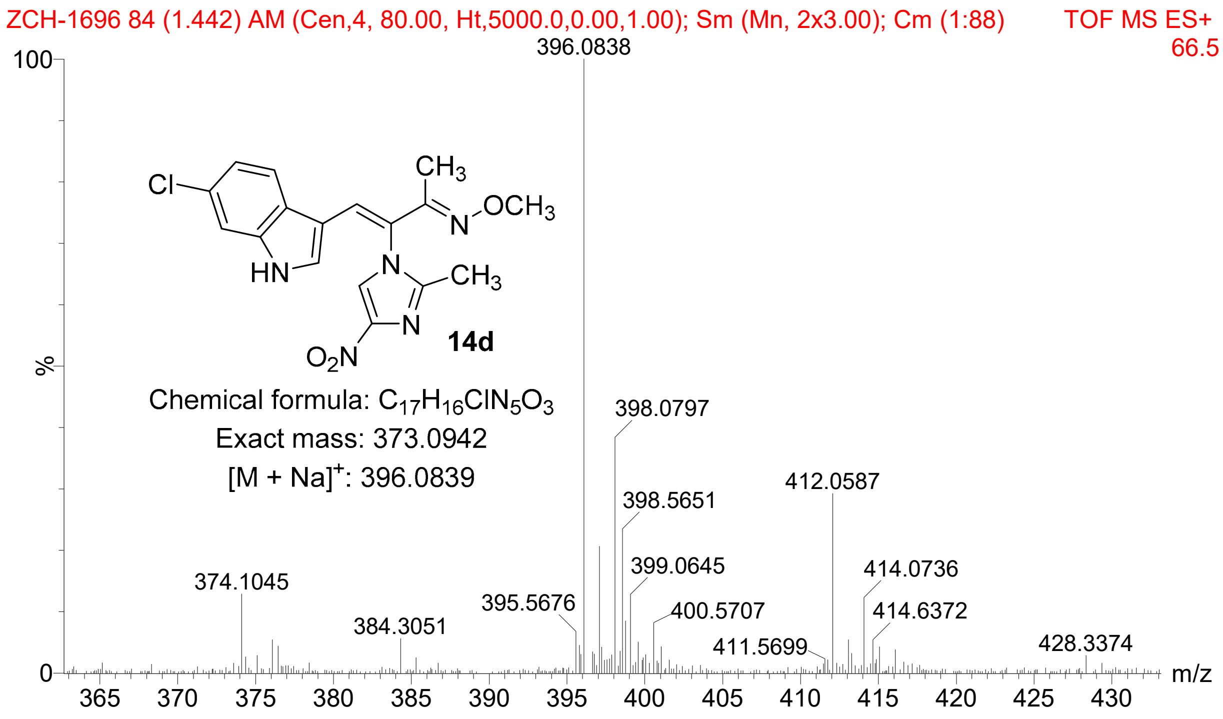

*7.15. Spectra of compound **14f***

^{1}H NMR spectrum (600 MHz, 25 °C, DMSO-d_6)

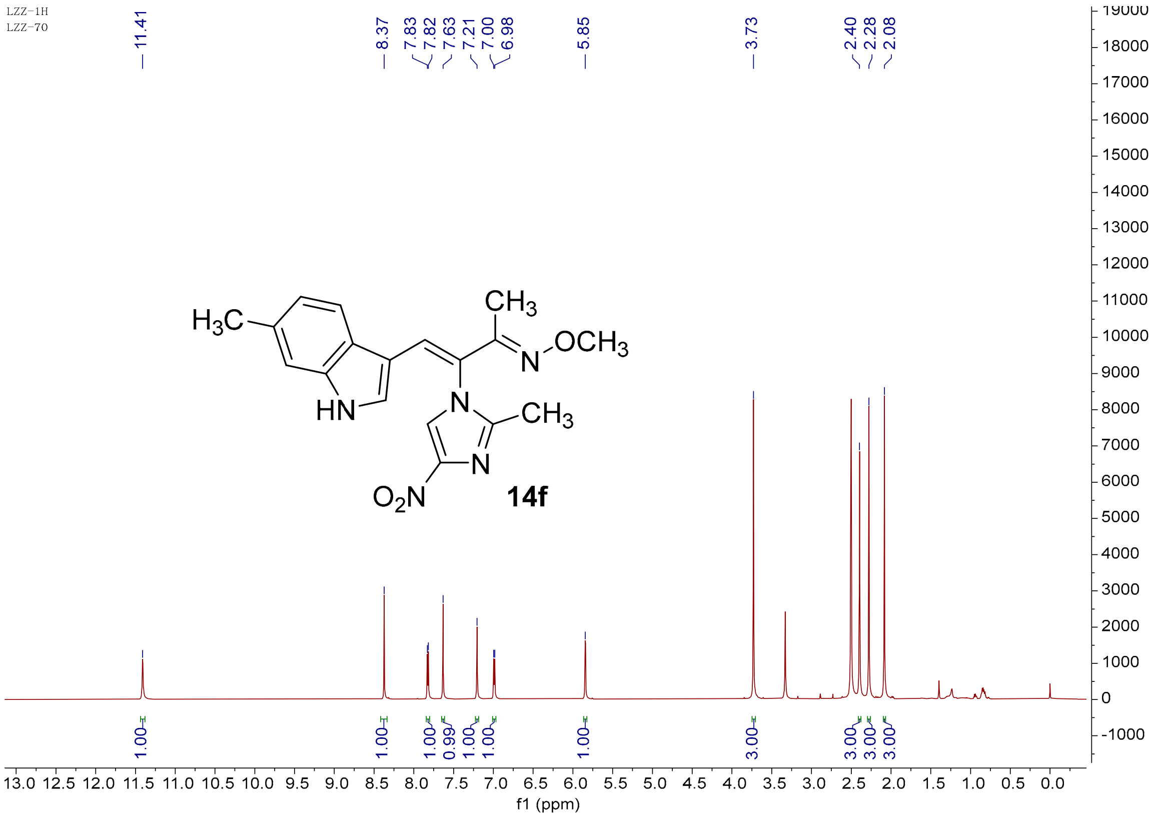

^{13}C NMR spectrum (151 MHz, 25 °C, DMSO-d_6)

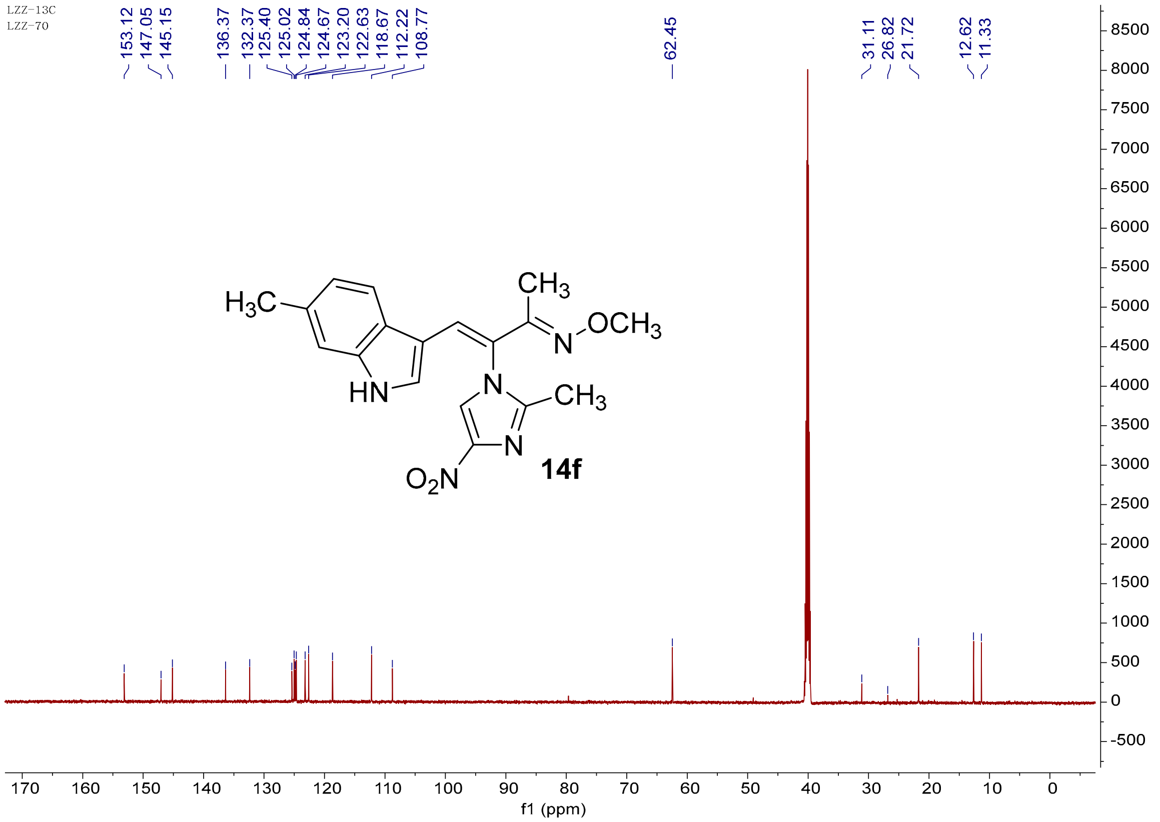

HRMS spectrum

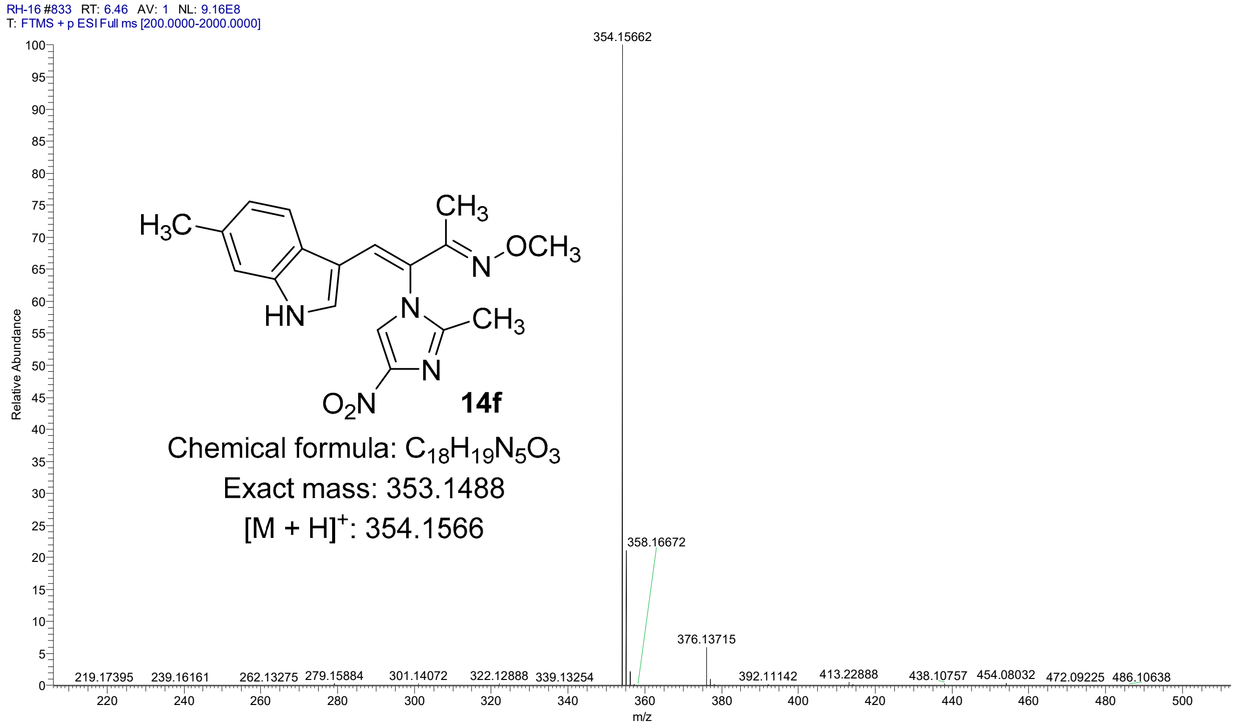

*7.16. Spectra of compound **16***

^{1}H NMR spectrum (400 MHz, 25 °C, DMSO-d_6)

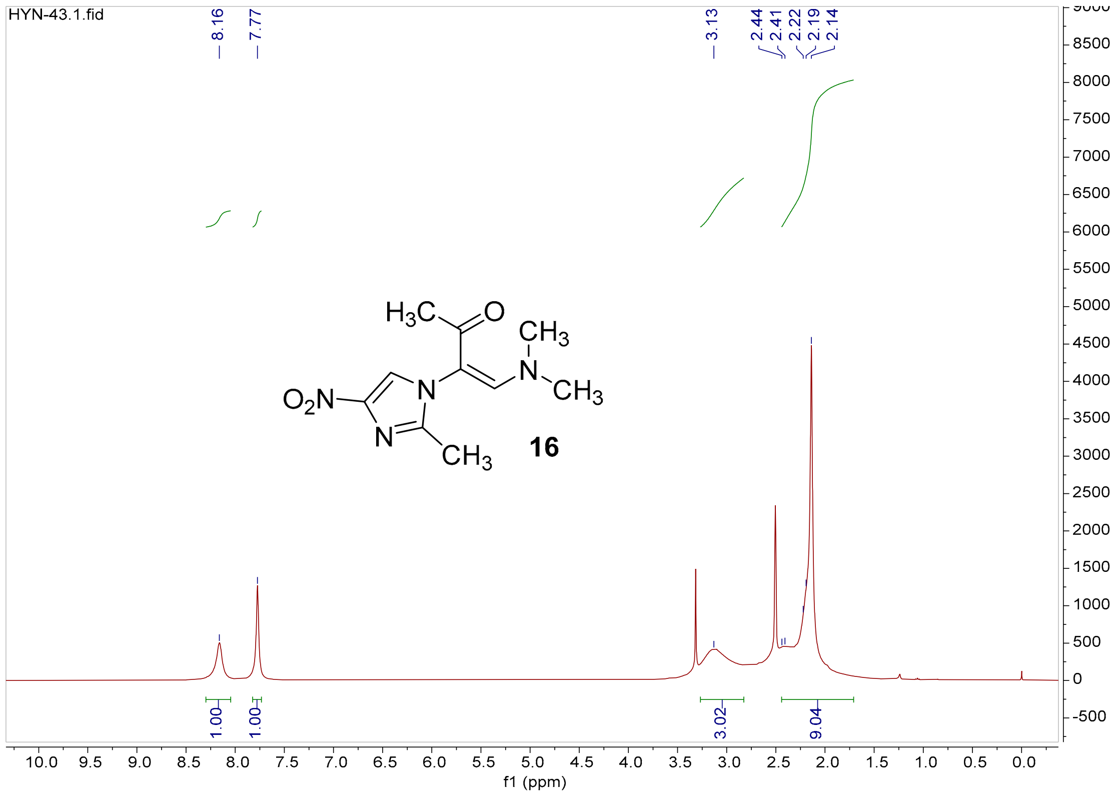

^{13}C NMR spectrum (101 MHz, 25 °C, DMSO-d_6)

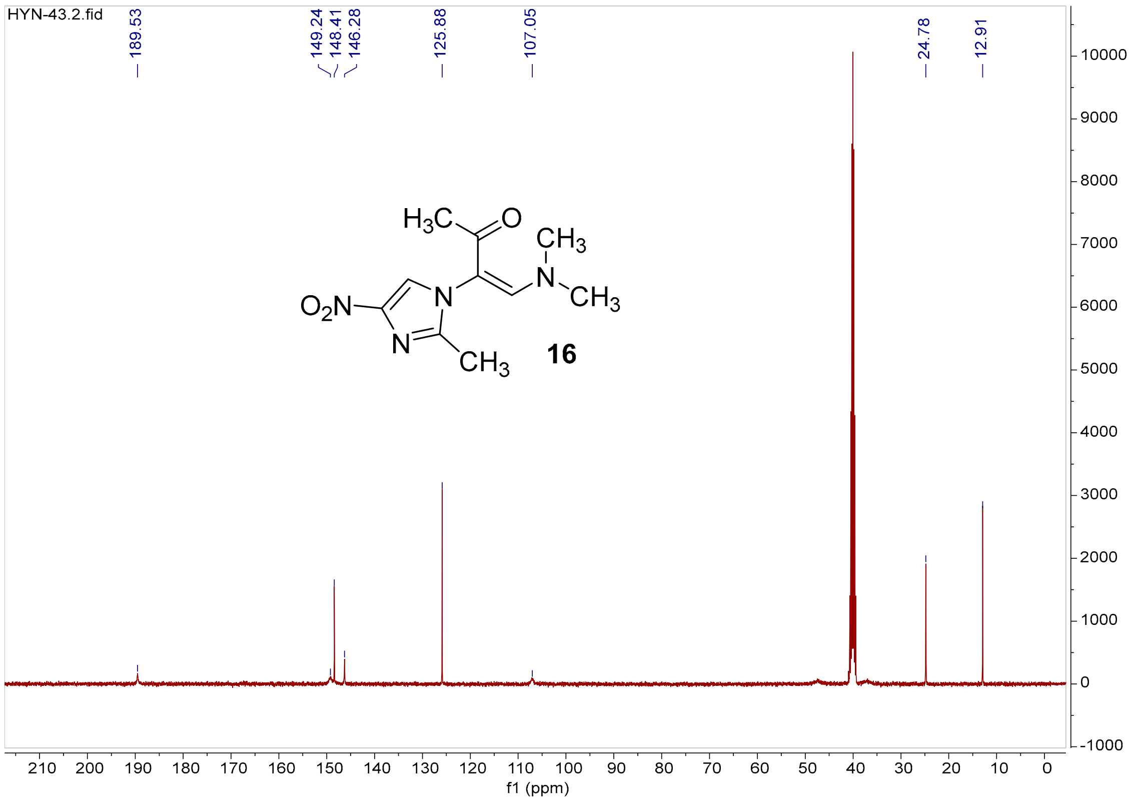

HRMS spectrum

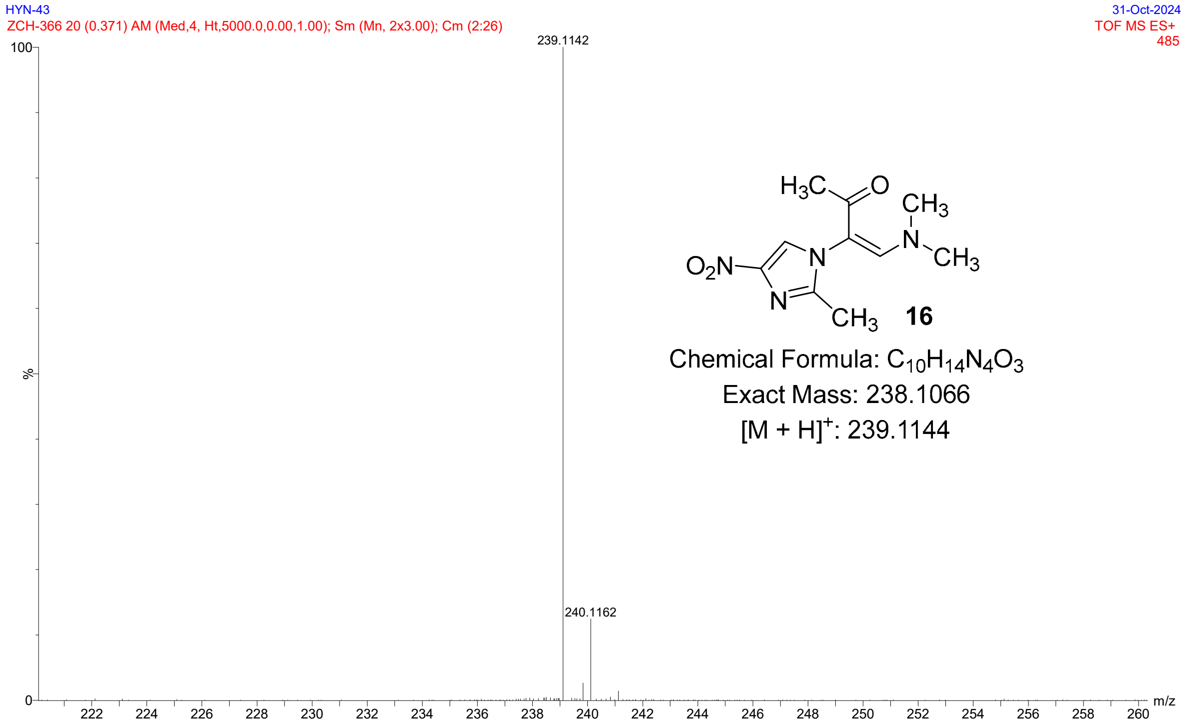

*7.17. Spectra of compound **18***

^{1}H NMR spectrum (400 MHz, 25 °C, DMSO-d_6)

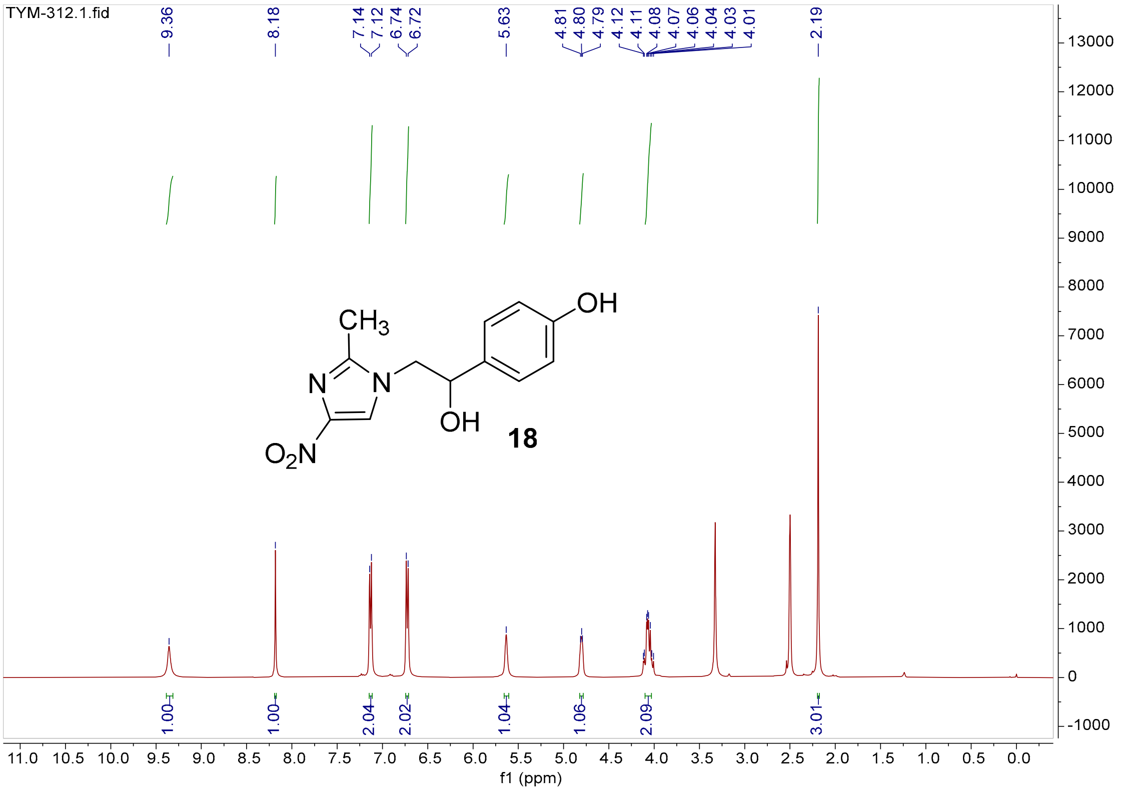

^{13}C NMR spectrum (101 MHz, 25 °C, DMSO-d_6)

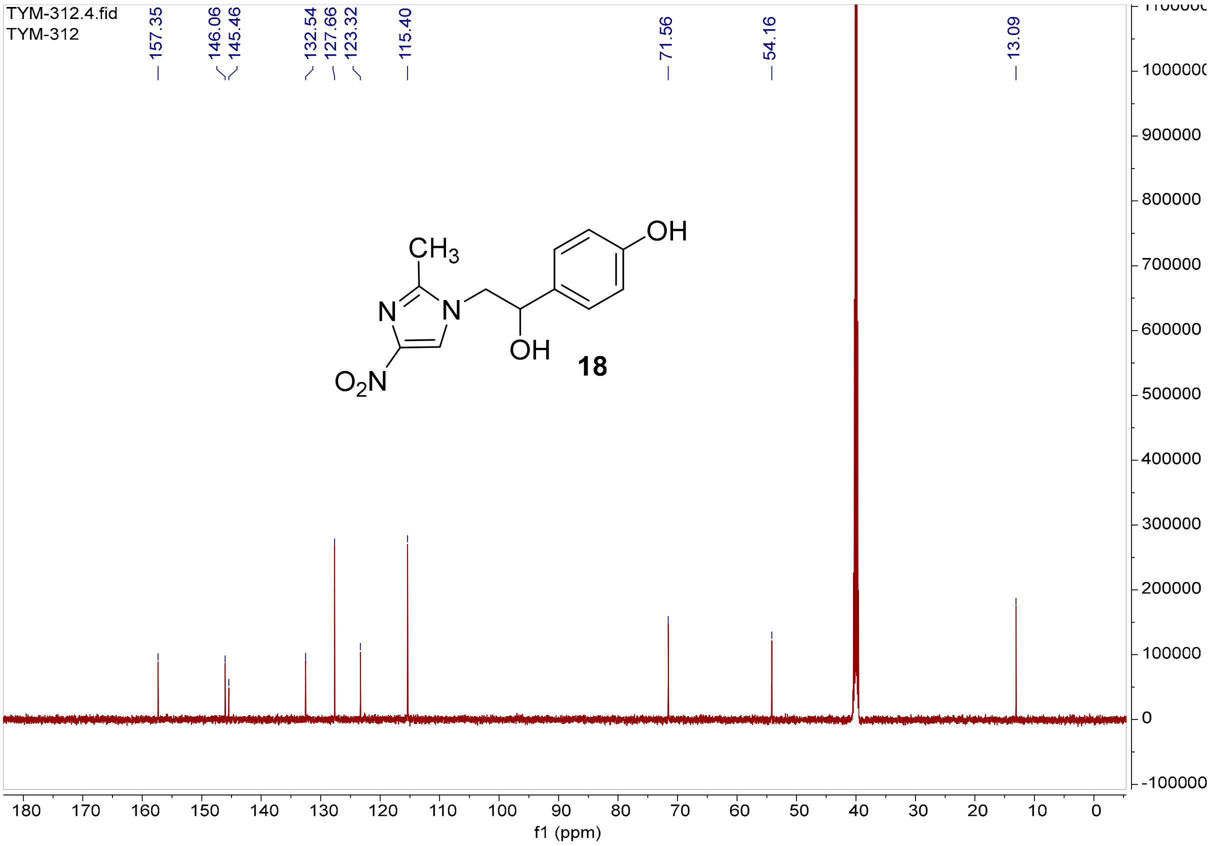

HRMS spectrum

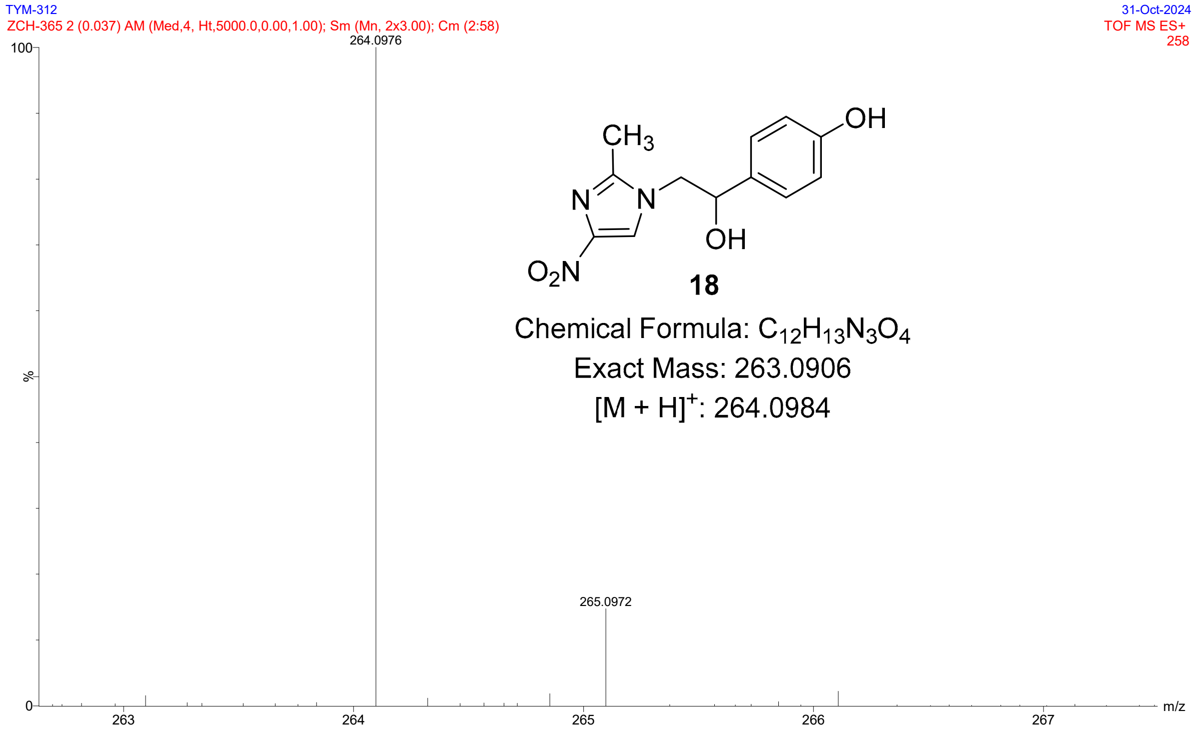

8. Analysis of X-ray diffraction

Table S2 Crystal data and structure refinement for compound 4.

Identification code	**compound 4**
Empirical formula	$C_{15}H_{16}N_4O_3$
Formula weight	300.32
Temperature/K	293(2)
Crystal system	monoclinic
Space group	$P2_1/n$
a/Å	10.2567(3)
b/Å	10.2134(2)
c/Å	15.5140(4)
α/°	90
β/°	109.042(3)
γ/°	90
Volume/Å^3	1536.26(7)
Z	4
ρ_{calc}g/cm^3	1.298
μ/mm^{-1}	0.771
F(000)	632.0
Crystal size/mm^3	0.25 × 0.11 × 0.06
Radiation	CuKα (λ = 1.54184)
2Θ range for data collection/°	9.148 to 134.144
Index ranges	$-9 \le h \le 12$, $-12 \le k \le 11$, $-18 \le l \le 17$
Reflections collected	5653
Independent reflections	2748 [R_{int} = 0.0183, R_{sigma} = 0.0226]
Data/restraints/parameters	2748/0/202
Goodness-of-fit on F^2	1.049
Final R indexes [I>=2σ (I)]	R_1 = 0.0454, wR_2 = 0.1258
Final R indexes [all data]	R_1 = 0.0536, wR_2 = 0.1345
Largest diff. peak/hole / e Å^{-3}	0.23/-0.16

Table S3 Fractional Atomic Coordinates (×10^4) and Equivalent Isotropic Displacement Parameters (Å^2×10^3) for compound 4. U_{eq} is defined as 1/3 of of the trace of the orthogonalised U_{IJ} tensor.

**Atom**	***x***	***y***	***z***	**U(eq)**
O1	4781(2)	3024.8(18)	4397.7(11)	97.4(6)
O2	6692(2)	3953(2)	5140.7(10)	101.3(6)
O3	4134.3(17)	3031.7(15)	56.8(9)	78.6(4)
N1	5738.7(18)	3772.5(15)	4445.8(10)	65.6(4)

N2	6738.4(15)	5288.9(17)	3602.8(10)	65.3(4)
N3	4980.8(14)	5354.7(15)	2313.1(9)	55.8(4)
N4	4484.6(17)	3822.1(17)	840.4(10)	64.1(4)
C1	5696.1(18)	4484.4(17)	3639.0(11)	56.7(4)
C2	4606.2(18)	4494.0(17)	2858.0(11)	56.5(4)
C3	6279.1(18)	5810(2)	2785.8(12)	62.3(5)
C4	7039(2)	6767(3)	2399.3(16)	88.8(7)
C5	3964.7(18)	7809.0(19)	2988.9(12)	61.0(4)
C6	4149(2)	8828(2)	3594.5(14)	69.6(5)
C7	4157(2)	10111(2)	3304.7(17)	76.8(6)
C8	3946(2)	10354(2)	2395.2(19)	84.4(7)
C9	3740(2)	9341(2)	1785.3(16)	74.4(6)
C10	3778.1(17)	8047.4(18)	2069.1(12)	58.8(4)
C11	3570.5(18)	7027(2)	1372.2(12)	61.2(4)
C12	4073.6(16)	5814.0(19)	1446.7(11)	56.1(4)
C13	3771.5(18)	4877(2)	685.4(11)	60.1(4)
C14	2679(2)	5185(3)	-205.2(13)	78.4(6)
C15	4859(3)	1820(2)	271.0(16)	86.1(7)

Table S4 Anisotropic Displacement Parameters ($Å^2 \times 10^3$) for compound 4. The Anisotropic displacement factor exponent takes the form: $-2\pi^2[h^2a^{*2}U_{11}+2hka^*b^*U_{12}+\ldots]$.

Atom	U_{11}	U_{22}	U_{33}	U_{23}	U_{13}	U_{12}
O1	108.5(13)	91.2(11)	76.8(10)	23.6(8)	9.0(9)	-26.6(10)
O2	111.3(13)	115.6(13)	53.1(8)	10.9(8)	-6.0(8)	-20.0(11)
O3	98.0(10)	81.4(9)	56.2(7)	2.4(7)	24.8(7)	14.8(8)
N1	79.1(10)	57.0(8)	50.2(8)	1.7(6)	6.8(7)	3.6(8)
N2	54.0(8)	80.4(10)	53.9(8)	1.5(7)	7.6(6)	-0.3(7)
N3	46.3(7)	68.3(9)	50.8(7)	5.7(6)	13.0(6)	5.4(6)
N4	66.7(9)	76.8(10)	51.3(8)	7.0(7)	22.7(7)	8.8(8)
C1	59.1(9)	56.8(9)	48.6(8)	0.8(7)	9.9(7)	5.5(8)
C2	52.9(9)	58.6(9)	53.7(9)	4.9(7)	11.6(7)	1.9(7)
C3	49.2(9)	80.5(12)	55.0(9)	1.0(8)	13.7(7)	0.3(8)
C4	63.5(12)	128(2)	73.3(13)	15.4(13)	20.4(10)	-16.0(13)
C5	61.4(10)	61.0(10)	58.2(9)	8.9(8)	16.2(7)	4.6(8)
C6	64.3(11)	74.3(12)	63.2(11)	3.9(9)	11.4(8)	6.3(9)
C7	57.7(11)	70.4(12)	100.0(16)	-9.9(11)	22.8(10)	2.3(9)
C8	83.5(14)	63.3(12)	120.2(19)	21.0(12)	52.1(14)	11.4(10)
C9	81.8(13)	70.6(12)	82.8(13)	22.9(10)	43.4(11)	20.2(10)
C10	51.5(9)	66.0(10)	61.8(9)	14.2(8)	22.2(7)	9.2(7)

C11	57.1(9)	78.4(12)	49.7(8)	15.1(8)	20.0(7)	10.2(8)
C12	45.3(8)	74.4(11)	48.1(8)	10.0(7)	14.5(7)	6.3(7)
C13	50.2(9)	82.4(12)	48.9(9)	6.0(8)	17.8(7)	6.1(8)
C14	69.2(12)	104.9(17)	52.6(10)	1.4(10)	8.3(8)	18.5(11)
C15	114.8(18)	75.1(13)	79.1(14)	15.2(11)	46.2(13)	18.8(13)

Table S5 Bond Lengths for compound 4.

Atom	Atom	Length/Å	Atom	Atom	Length/Å
O1	N1	1.227(2)	C3	C4	1.493(3)
O2	N1	1.210(2)	C5	C6	1.373(3)
O3	N4	1.405(2)	C5	C10	1.398(3)
O3	C15	1.426(3)	C6	C7	1.386(3)
N1	C1	1.436(2)	C7	C8	1.378(4)
N2	C1	1.364(2)	C8	C9	1.371(3)
N2	C3	1.312(2)	C9	C10	1.389(3)
N3	C2	1.359(2)	C10	C11	1.466(3)
N3	C3	1.375(2)	C11	C12	1.333(3)
N3	C12	1.441(2)	C12	C13	1.472(3)
N4	C13	1.280(2)	C13	C14	1.501(2)
C1	C2	1.354(2)			

Table S6 Bond Angles for compound 4.

Atom	Atom	Atom	Angle/°	Atom	Atom	Atom	Angle/°
N4	O3	C15	109.29(15)	C6	C5	C10	120.58(18)
O1	N1	C1	117.78(15)	C5	C6	C7	120.6(2)
O2	N1	O1	123.18(18)	C8	C7	C6	119.1(2)
O2	N1	C1	119.02(18)	C9	C8	C7	120.6(2)
C3	N2	C1	104.18(15)	C8	C9	C10	121.1(2)
C2	N3	C3	107.95(14)	C5	C10	C11	124.58(17)
C2	N3	C12	124.28(14)	C9	C10	C5	118.00(19)
C3	N3	C12	127.29(15)	C9	C10	C11	117.38(17)
C13	N4	O3	110.61(15)	C12	C11	C10	129.63(16)
N2	C1	N1	122.66(15)	N3	C12	C13	116.08(16)
C2	C1	N1	124.40(17)	C11	C12	N3	119.84(17)
C2	C1	N2	112.84(16)	C11	C12	C13	124.08(16)
C1	C2	N3	104.06(16)	N4	C13	C12	116.00(15)
N2	C3	N3	110.98(17)	N4	C13	C14	123.94(18)

N2	C3	C4	125.71(17)	C12	C13	C14	120.06(17)
N3	C3	C4	123.31(17)				

Table S7 Torsion Angles for compound 4.

A	B	C	D	Angle/°	A	B	C	D	Angle/°
O1	N1	C1	N2	-176.54(19)	C3	N3	C12	C13	114.4(2)
O1	N1	C1	C2	7.4(3)	C5	C6	C7	C8	1.5(3)
O2	N1	C1	N2	4.9(3)	C5	C10	C11	C12	-32.9(3)
O2	N1	C1	C2	-171.2(2)	C6	C5	C10	C9	-2.1(3)
O3	N4	C13	C12	-178.07(15)	C6	C5	C10	C11	-179.81(17)
O3	N4	C13	C14	2.2(3)	C6	C7	C8	C9	-0.4(3)
N1	C1	C2	N3	175.82(16)	C7	C8	C9	C10	-1.9(3)
N2	C1	C2	N3	-0.6(2)	C8	C9	C10	C5	3.1(3)
N3	C12	C13	N4	-8.9(2)	C8	C9	C10	C11	-178.97(19)
N3	C12	C13	C14	170.83(17)	C9	C10	C11	C12	149.4(2)
C1	N2	C3	N3	0.1(2)	C10	C5	C6	C7	-0.2(3)
C1	N2	C3	C4	-179.7(2)	C10	C11	C12	N3	-0.9(3)
C2	N3	C3	N2	-0.5(2)	C10	C11	C12	C13	179.53(17)
C2	N3	C3	C4	179.3(2)	C11	C12	C13	N4	170.63(17)
C2	N3	C12	C11	105.8(2)	C11	C12	C13	C14	-9.6(3)
C2	N3	C12	C13	-74.6(2)	C12	N3	C2	C1	-171.84(16)
C3	N2	C1	N1	-176.17(17)	C12	N3	C3	N2	171.72(17)
C3	N2	C1	C2	0.3(2)	C12	N3	C3	C4	-8.5(3)
C3	N3	C2	C1	0.6(2)	C15	O3	N4	C13	-175.21(17)
C3	N3	C12	C11	-65.2(2)					

Table S8 Hydrogen Atom Coordinates (Å×10^4) and Isotropic Displacement Parameters (Å^2×10^3) for compound 4.

Atom	*x*	*y*	*z*	U(eq)
H2	3790	4021	2725	68
H4A	6744	6677	1748	133
H4B	8012	6600	2647	133
H4C	6850	7641	2554	133
H5	3964	6953	3193	73
H6	4269	8656	4204	83
H7	4302	10799	3718	92
H8	3943	11212	2194	101
H9	3571	9523	1171	89

H11	3003	7262	792	73
H14A	3098	5570	-615	118
H14B	2027	5789	-103	118
H14C	2212	4393	-468	118
H15A	4606	1270	-260	129
H15B	4625	1391	752	129
H15C	5834	1982	465	129

References

1. Li, Z.Z.; Tangadanchu, V.K.R.; Battini, N.; Bheemanaboina, R.R.Y.' Zang, Z.L.' Zhang, S.L.; Zhou, C.H. Indole-nitroimidazole conjugates as efficient manipulators to decrease the genes expression of methicillin-resistant *Staphylococcus aureus*. *Eur J Med Chem* **2019**, *179*, 723–735.

2. Olender, D.; Zwawiak, J.; Lukianchuk, V.; Roman, L.; Kropacz, A.; Fojutowski, A.; Zaprutko, L. Synthesis of some N-substituted nitroimidazole derivatives as potential antioxidant and antifungal agents. *Eur J Med Chem* **2009**, *44*, 645–652.
